# *Zfp57* Exerts Maternal and Sexually Dimorphic Effects on Genomic Imprinting

**DOI:** 10.3389/fcell.2022.784128

**Published:** 2022-02-02

**Authors:** Zhen Xu, Jiajia Shi, Yu Zhang, Yuhan Liu, Junzheng Zhao, Qian Chen, Chenglin Song, Shuhui Geng, Wei Xie, Feizhen Wu, Yun Bai, Yang Yang, Xiajun Li

**Affiliations:** ^1^ School of Life Science and Technology, ShanghaiTech University, Shanghai China; ^2^ Center for Stem Cell Biology and Regenerative Medicine, MOE Key Laboratory of Bioinformatics, Tsinghua-Peking Center for Life Sciences, School of Life Sciences, Tsinghua University, Beijing, China; ^3^ Institutes of Biomedical Sciences, Shanghai Medical College of Fudan University, Shanghai, China

**Keywords:** genomic imprinting, imprinting control region (ICR), imprinted genes, maternal effect, gender, sexually dimorphic, allelic expression, RNA-seq, WGBS, bisulfite sequencing

## Abstract

*Zfp57* has both maternal and zygotic functions in mouse. It maintains genomic imprinting at most known imprinted regions and controls allelic expression of the target imprinted genes in mouse embryos. The DNA methylation imprint at many imprinting control regions (ICRs) is lost when both maternal and zygotic *Zfp57* are absent in *Zfp57* maternal–zygotic mutant mouse embryos. Interestingly, we found that DNA methylation at a few ICRs was partially lost without maternal *Zfp57* in *Zfp57* heterozygous mouse embryos derived from *Zfp57* homozygous female mice. This suggests that maternal *Zfp57* is essential for the maintenance of DNA methylation at a small subset of imprinted regions in mouse embryos. This maternal effect of *Zfp57* was applied to allelic expression switch as well as expression levels of the corresponding imprinted genes. It is rather surprising that DNA methylation imprint was affected differently at *Rasgrf1* and *AK008011* imprinted regions in the female or male *Zfp57* maternal–zygotic mutant embryos, with more significant loss of DNA methylation observed in the male mutant embryos. Loss of ZFP57 resulted in gender-specific differences in allelic expression switch and expression level changes of some imprinted genes in female or male mutant embryos. These results indicate maternal and sexually dimorphic effects of ZFP57 on genomic imprinting in mouse.

## Introduction

Genomic imprinting is a kind of parental effect on the progeny that is established in the female or male germline (Bartolomei and Ferguson-Smith, 2011; Li, 2013; Tucci et al., 2019). It is essential for mammalian embryonic growth and development. Most of approximately 150 known imprinted genes are clustered in over 20 known imprinted regions with each harboring a few imprinted genes (Monk et al., 2019). They are co-regulated by a *cis*-acting imprinting control region (ICR) containing germline-derived differential DNA methylation (Barlow and Bartolomei, 2014; Zeng and Chen, 2019). Based on definition, imprinted genes exhibit parent-of-origin–dependent monoallelic expression, although some are preferentially expressed from one parental allele, and others may be imprinted only in some tissues or organs (Williamson et al., 2004; Marcho et al., 2015; Plasschaert and Bartolomei, 2015; Freschi et al., 2018; Hsiao et al., 2019; Monk et al., 2019; Jiang et al., 2021; Prickett et al., 2021). Imprinting shares some similarities to other monoallelic gene expression phenomena in mammals (Lee and Bartolomei, 2013; Chess, 2016; Khamlichi and Feil, 2018; Bar and Benvenisty, 2019).

ZFP57 and ZFP445 are KRAB zinc finger proteins that play important roles in maintaining genomic imprinting (Hirasawa and Feil, 2008; Juan and Bartolomei, 2019; Tucci et al., 2019; Hanna and Kelsey, 2021). They are partially redundant in the maintenance of genomic imprinting, with ZFP57 being more dominant in mouse embryos (Takahashi et al., 2015; Takahashi et al., 2019). Human ZFP57 has similar functions in genomic imprinting, and mutations in human *ZFP57* result in a number of human diseases, including transient neonatal diabetes (Mackay et al., 2008; Takikawa et al., 2013b; Monteagudo-Sanchez et al., 2020). Mouse *Zfp57* is a maternal–zygotic effect gene (Li et al., 2008; Shamis et al., 2015). It has both maternal and zygotic functions. Loss of both maternal and zygotic *Zfp57* in the maternal–zygotic mutant (M^−^Z^-^) embryos results in loss of DNA methylation imprinting at most ICRs and deregulation of target-imprinted genes at these imprinted regions, whereas loss of just zygotic *Zfp57* in the zygotic mutant (M^+^Z^−^) embryos cause partial loss of DNA methylation imprint at these ICRs (Jiang et al., 2021). ZFP57 binds to almost all known ICRs, with higher binding affinity for the methylated DNA (Li et al., 2008; Quenneville et al., 2011; Liu et al., 2012; Strogantsev et al., 2015; Riso et al., 2016; Takahashi et al., 2019). Allelic expression switch occurs at some target imprinted genes when ZFP57 is lost in M^−^Z^−^ embryos (Jiang et al., 2021).

Sexually dimorphic effect has been reported in many studies. There are gender-dependent phenotypes in cardiovascular diseases (Deegan et al., 2021; Walker et al., 2021). Males and female behave differently in neural behavior and brain disorders (Chen et al., 2019; Pfaff and Barbas, 2019; Simchovitz-Gesher and Soreq, 2020; Serrano-Saiz and Isogai, 2021). Gender-specific differences have been observed in gene expression and immune response (Gal-Oz et al., 2019). Even for COVID-19, males appear to be more susceptible to the viral infection and disease severity (Li and Li, 2020; Takahashi et al., 2020).

Sexual dimorphism has also been observed in genomic imprinting. Loss of the *Peg3* imprinted gene causes more severe defects in the male placentas than the female ones (Tunster et al., 2018). There is an increased risk of type 2 diabetes (T2D) upon reduced expression of the *KLF14* imprinted gene at the *PEG1* imprinted region in females (Small et al., 2018). Cognition in childhood is impacted by the methylation at the *PEG1/MEST* imprinted region, with stronger effect observed in the males (Lorgen-Ritchie et al., 2019). Many miRNAs at the *DLK1-DIO3* imprinted region were reported to show increased expression in male patients with multiple sclerosis (MS) but not in the female MS patients although their expression appeared to be lower in the healthy males compared with the healthy females (Baulina et al., 2019). Gender has an effect on the monoallelic expression of *ATP10A* that is more preferentially maternally expressed in the female human brains (Hogart et al., 2008). There are a few other studies indicating the gender-specific effects on DNA methylation at the imprinted regions or expression of the imprinted genes (Agba et al., 2017; Wang et al., 2020a; Wang et al., 2020b).

We noticed that the DNA methylation level was much higher at the *Rasgrf1* ICR in the two *Zfp57* maternal–zygotic mutant (M^−^Z^-^) embryos than at the *Rasgrfl* ICR in the other two M^−^Z^−^ embryos in our previous study (Jiang et al., 2021). Interestingly, we found that DNA methylation imprint was more susceptible to loss of ZFP57 at the *Rasgrf1* and *AK008011* ICRs in the male M^−^Z^-^ embryos than in the female M^−^Z^−^ embryos. Furthermore, loss of ZFP57 caused sexually dimorphic effects on allelic expression switch or expression levels of some imprinted genes in mouse embryos. This is the first study to show sexually dimorphic effects of ZFP57 on genomic imprinting in mouse embryos. *Zfp57* exhibits maternal–zygotic effect in genomic imprinting and embryonic lethality (Li et al., 2008; Takahashi et al., 2015; Takahashi et al., 2019; Jiang et al., 2021). In this study, we also found that loss of just maternal *Zfp57* caused loss of DNA methylation at a subset of ICRs and loss of parent-of-origin–dependent expression of some imprinted genes in mouse embryos, indicating crucial maternal effect of *Zfp57* on a small subset of imprinted regions.

## Results

### More Severe Loss of DNA Methylation Imprint at the Two Imprinting Control Regions in the Male *Zfp57* Maternal–Zygotic Mutant Embryos

Whole-genome bisulfite sequencing (WGBS) was performed to examine DNA methylation in the 129/DBA hybrid *Zfp57* ^*+/−*^ (M^+^Z^+^), *Zfp57* ^*−/+*^ (M^−^Z^+^), and *Zfp57* ^*−/−*^ (M^−^Z^−^) embryos derived from timed mating with *Zfp57* ^*+/−*^ or *Zfp57* ^*−/−*^ 129 female mice being crossed with *Zfp57* ^*+/−*^ (DBA*) male mice mainly on the DBA/2J genetic background as reported in the previous study (Jiang et al., 2021). We found that the two M^−^Z^-^ mutant embryos had much higher levels of DNA methylation at the *Rasgrf1* imprinted region than two other M^−^Z^-^ mutant embryos (Jiang et al., 2021). We tested these embryo samples to find out what may cause the differences of DNA methylation at this imprinted region when ZFP57 was absent.

**FIGURE 1:**
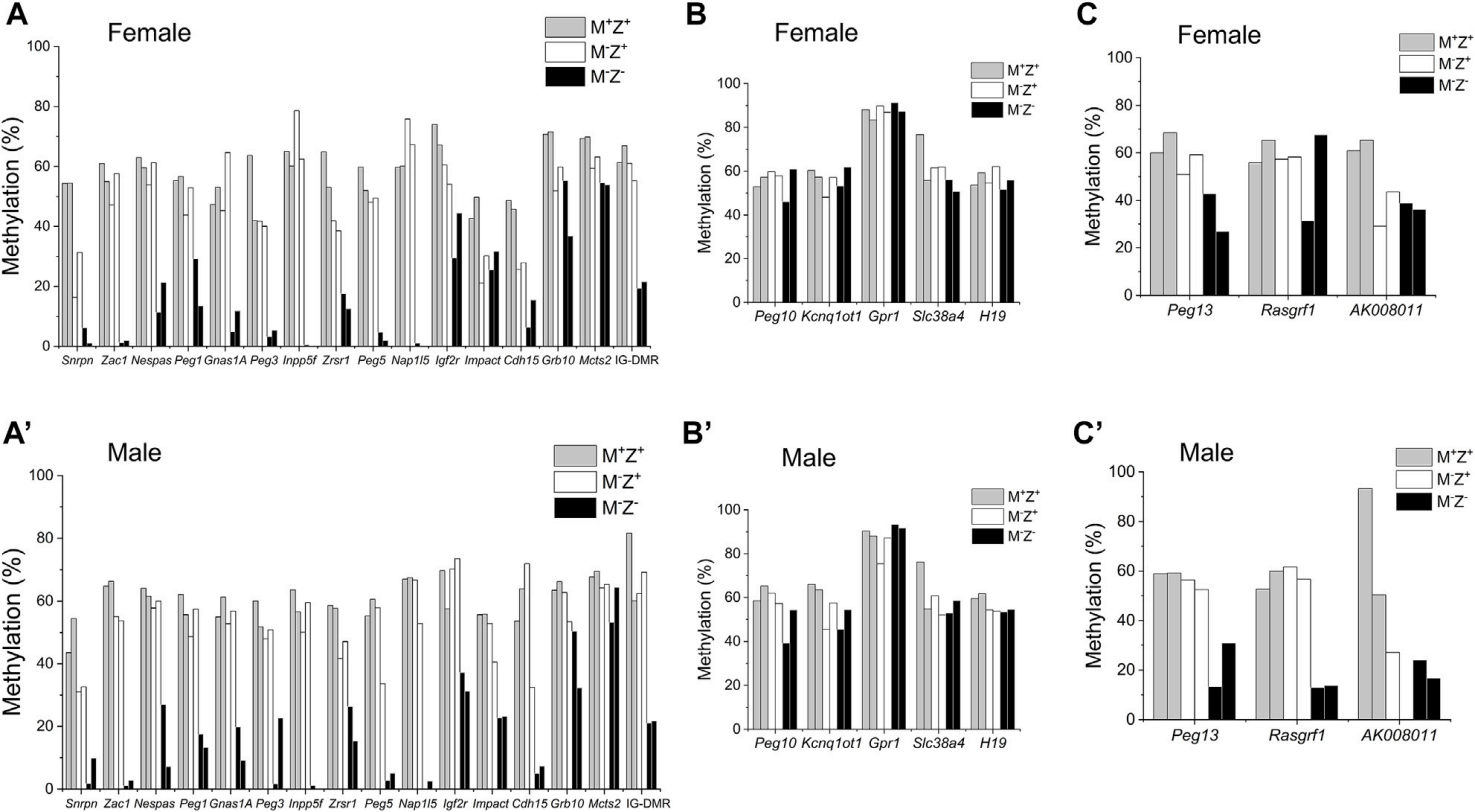
DNA methylation at three imprinted regions appeared to be more severely lost in male *Zfp57* maternal–zygotic mutant embryos than in the female ones. Genomic DNA samples were isolated from the *Zfp57* ^*+/−*^ (M^+^Z^+^), *Zfp57* ^*−/+*^ (M^−^Z^+^), and *Zfp57* ^*−/−*^ (M^−^Z^-^) hybrid E13.5 embryos from the timed mating between *Zfp57* ^*+/−*^ (or *Zfp57* ^*−/−*^) 129 female mice and *Zfp57* ^*+/−*^ (DBA*) male mice mainly on the DBA/2J genetic background as reported in the previous study (Jiang et al., 2021). Two female embryos of each genotype and two male embryos of each genotype were used in this study. Whole-genome bisulfite sequencing (WGBS) was carried out to examine DNA methylation at previously known 21 maternally methylated and three paternally methylated ICRs of the imprinted regions. **(A–C)** ICR methylation in the female embryos. **(A’–C’)** ICR methylation in the male embryos. **(A,A’)** DNA methylation imprint appeared to be similarly affected at 16 ICRs in the female or male maternal–zygotic mutant (M^−^Z^-^) embryos. These include the *Snrpn*, *Zac1*, *Nespas*, *Peg1*, *Gnas1A*, *Peg3*, *Inpp5f*, *Zrsr1*, *Peg5*, *Nap1l5*, *Igf2r*, *Impact*, *Cdh15*, *Grb10*, and *Mcts2* ICRs as well as the IG-DMR. **(B,B’)** DNA methylation imprint was mostly retained at the *Peg10*, *Kcnq1ot1*, *Gpr1*, *Slc38A4*, and *H19* ICRs in the female or male maternal–zygotic mutant (M^−^Z^-^) embryos. **(C,C’)** DNA methylation imprint appeared to be more severely lost at the *Peg13*, *Rasgrf1*, and *AK008011* ICRs in the male maternal–zygotic mutant (M^−^Z^-^) embryos compared with the female maternal–zygotic mutant (M^−^Z^-^) embryos. There were no sequence reads at the *AK008011* ICR in the second male M^−^Z^+^ embryo in **(C’)**.

**FIGURE 2:**
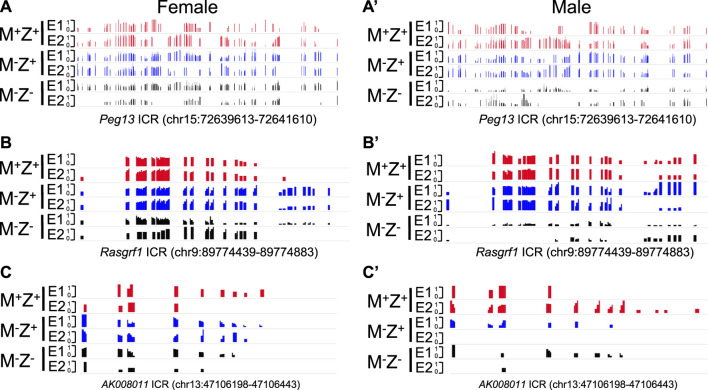
Methylation level was lower at the *Peg13*, *Rasgrf1*, and *AK008011* ICRs in male *Zfp57* maternal–zygotic mutant embryos than that in the female ones on the methylation IGV plot. Genomic DNA samples were isolated from *Zfp57* ^*+/+*^ (M^+^Z^+^), *Zfp57* ^*−/+*^ (M^−^Z^+^), and *Zfp57* ^*−/−*^ (M^−^Z^-^) hybrid 129/DBA embryos that were generated from the timed mating as reported in the previous study (Jiang et al., 2021). They were subjected to whole-genome bisulfite sequencing (WGBS) (Jiang et al., 2021). The methylation IGV plot with the scale of 0–1 is shown for the ICRs of the *Peg13*, *Rasgrf1*, and *AK008011* imprinted regions in two female embryos (E1 and E2) or two male embryos (E1 and E2) of the M^+^Z^+^, M^−^Z^+^, and M^−^Z^-^ genotypes. **(A,A’)** Methylation IGV plot of *Peg13* ICR (mm9, chr15:72,639,613–72,641,610) in the female **(A)** or male **(A’)** embryos. **(B,B’)** Methylation IGV plot of *Rasgrf1* ICR (mm9, chr9:89,774,439–89,774,883) in the female **(B)** or male **(B’)** embryos. **(C,C’)** Methylation IGV plot of *AK008011* ICR (mm9, chr13:47,106,198–47,106,443) in the female **(C)** or male **(C’)** embryos. There were no sequence reads at the *AK008011* ICR in the E2 sample of male M^−^Z^+^ in **(C’)**. There were only six CpG sites with at least three unique reads in the E2 sample of female M^−^Z^-^ in **(C)** and just two CpG sites with at least three unique reads in the E2 sample of male M^−^Z^-^ in **(C’)**.

We wonder if gender might contribute to the effects of ZFP57 on DNA methylation at the *Rasgrf1* ICR as well as other ICRs. This may have resulted in the substantial differences of DNA methylation observed at the *Rasgrf1* ICR among four M^−^Z^−^ embryos in the previous WGBS study. There was no significant difference in DNA methylation at all 24 known ICRs comparing female M^+^Z^+^ embryos with the male M^+^Z^+^ embryos, except that DNA methylation was slightly significantly increased at the *Nap1I5* ICR but decreased at the *Grb10* ICR in the male M^+^Z^+^ embryos compared with the female M^+^Z^+^ embryos (Supplementary Figure S1A). DNA methylation was similar at all ICRs except for the slight increase of DNA methylation at the *Peg3* ICR in the comparison of male M^−^Z^+^ embryos with the female ones (Supplementary Figure S1B). DNA methylation was significantly reduced at the *AK008011* ICR in the comparison of male M^−^Z^−^ embryos with the female ones (Supplementary Figure S1C). It was dramatically reduced at the *Rasgrf1* ICR in the male M^−^Z^-^ embryos compared with the female ones, although it was not statistically significant, which will be discussed later (Supplementary Figure S1C). Therefore, we examined DNA methylation at the ICRs in individual embryos subjected to WGBS in our previous study (Jiang et al., 2021). As expected, DNA methylation was similarly lost at most ICRs in the female or male M^−^Z^−^ mutant embryos (Figures 1A, 1A’). It was largely retained at five imprinted regions (*Peg10*, *Kcnq1ot1*, *Gpr1*, *Slc38a4*, and *H19*) in both female or male M^−^Z^-^ mutant embryos (Figures 1B, 1B’). Intriguingly, DNA methylation appeared to be more severely lost at the *Peg13*, *Rasgrf1*, and *AK008011* ICRs in the male M^−^Z^-^ mutant embryos than in the female M^−^Z^-^ mutant embryos (Figures 1C, 1C’)*.* This finding can be easily visualized on the methylation IGV plots for three ICRs in these M^+^Z^+^, M^−^Z^+^, and M^−^Z^-^ embryos (Figure 2). One of two male M^−^Z^−^ embryos displayed more severe loss of methylation at the *Peg13* ICR, whereas DNA methylation was more severely lost at the *Rasgrf1* and *AK008011* ICRs in both male M^−^Z^−^ embryos (Figures 1, 2). We also found that one female M^−^Z^−^ mutant embryo had reduced DNA methylation at the *Rasgrf1* ICR, whereas DNA methylation did not appear to be lost at the *Rasgrf1* ICR in the other female M^−^Z^−^ mutant embryos (Figures 1C, 2B).

DNA methylation was significantly reduced at the *Peg13* ICR in the male M^−^Z^−^ embryos compared with male M^+^Z^+^ and M^-^Z^+^ embryos, whereas it was close to being significantly reduced at the *Peg13* ICR in the female M^−^Z^−^ embryos compared with female M^+^Z^+^ embryos (Supplementary Figures S1D, S1D’). Indeed, DNA methylation was significantly lost at the *Rasgrf1* ICR in the male M^−^Z^−^ embryos compared with male M^+^Z^+^ and M^−^Z^+^ embryos, whereas it was not much different at the *Rasgrf1* ICR comparing female M^−^Z^−^ embryos with female M^+^Z^+^ or M^−^Z^+^ embryos (Supplementary Figures S1D, S1D’). DNA methylation at the *AK008011* ICR was significantly reduced in both female M^−^Z^+^ and M^−^Z^−^ embryos compared with the female M^+^Z^+^ embryos (Supplementary Figure S1D). It was close to being significantly reduced in both male M^−^Z^+^ and M^−^Z^−^ embryos compared with the male M^+^Z^+^ embryos (Supplementary Figure S1D’). It was even more significantly lost at the *AK008011* ICR in the male M^−^Z^−^ embryos than in the female M^−^Z^−^ embryos (Supplementary Figure S1C). We performed similar statistical comparisons for DNA methylation at the *Snrpn*, *Impact*, and *Cdh15* ICRs that will be discussed for the maternal effect of *Zfp57* on their DNA methylation below (Supplementary Figure S1E, S1E’).

**FIGURE 3:**
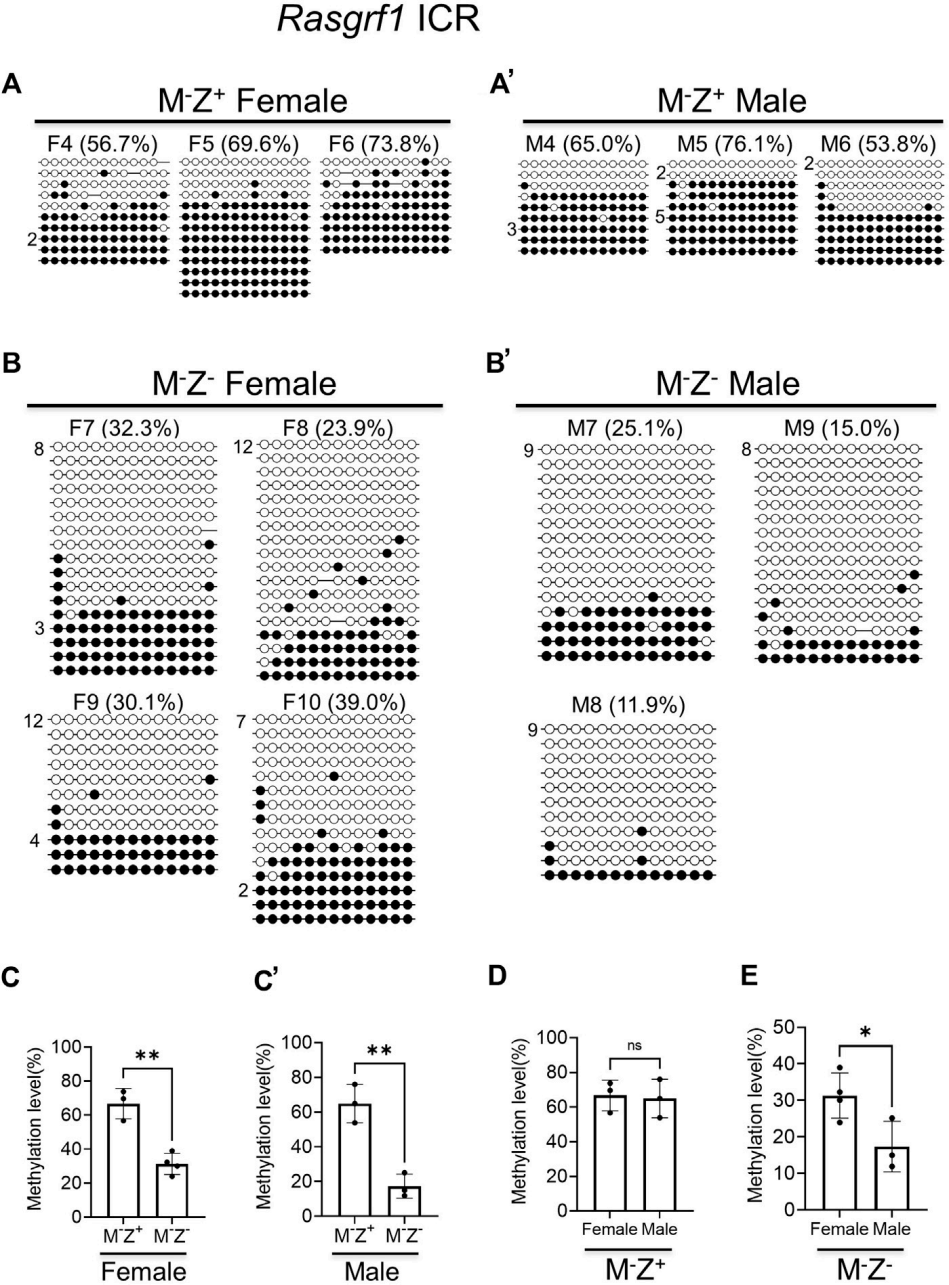
DNA methylation at the *Rasgrf1* ICR was more severely lost in the male maternal–zygotic mutant embryos than in the female ones based on bacterial colony bisulfite sequencing analysis. Genomic DNA samples were isolated from at least three female or male *Zfp57* ^*−/+*^ (M^−^Z^+^) and *Zfp57* ^*−/−*^ (M^−^Z^-^) E13.5 embryos derived from the timed mating between *Zfp57* homozygous mutant female mice and heterozygous male mice. They were subjected to bisulfite mutagenesis followed by bacterial colony sequencing of the 321-bp bisulfite PCR product of the *Rasgrf1* ICR region. The unconverted cytosine **(C)** residues were used to determine the unique clones for the bisulfite colony sequencing. Each row represents a unique clone, with the filled black circles for methylated CpG sites and unfilled white circles for unmethylated CpG sites. The number in front of a unique clone shows the number of sequenced non-unique clones containing the same sequence that cannot be distinguished by unconverted **(C)** residues. The percentage of DNA methylation in **(A,B’)** was calculated based on the number of methylated CpG sites divided by the total number of CpG sites for the sequenced unique clones of the bisulfite PCR product. Two-tailed Student’s *t* test was used for statistical analysis of DNA methylation level differences between two different genotypes of the same gender **(C–C’)** or within the same genotype between males and females **(D,E)**. Statistical significance: *, *p* < 0.05; **, *p* < 0.01; and ***, *p* < 0.001. **(A)** Three M^−^Z^+^ female embryos (F4–F6). **(A’)** Three M^−^Z^+^ male embryos (M4–M6). **(B)** Four M^−^Z^-^ female embryos (F7–F10). **(B’)** Three M^−^Z^-^ male embryos (M7–M9). **(C)** DNA methylation at the *Rasgrf1* ICR was significantly reduced in four M^−^Z^-^ female embryos compared with three M^−^Z^+^ female embryos. **(C’)**, DNA methylation at the *Rasgrf1* ICR was significantly reduced in the three M^−^Z^-^ male embryos compared with three M^−^Z^+^ male embryos. **(D)** DNA methylation at the *Rasgrf1* ICR was similar comparing three M^−^Z^+^ female embryos with three M^−^Z^-^ male embryos. **(E)** DNA methylation at the *Rasgrf1* ICR was significantly reduced in the three M^−^Z^-^ male embryos compared with four M^−^Z^-^ female embryos.

To confirm if DNA methylation was, indeed, more severely lost at the *Rasgrf1* ICR in the male M^−^Z^−^ mutant embryos than in the female M^−^Z^−^ mutant embryos, we carried out bacterial colony bisulfite sequencing analysis of this *Rasgrf1* ICR with another set of M^−^Z^+^ and M^−^Z^−^ embryos that consisted of 3–4 female and male embryos for each genotype (Figure 3). Since DNA methylation levels were similar at the *Rasgrf1* ICR in the M^+^Z^+^ and M^−^Z^+^ embryos of the same gender based on WGBS (Figures 1C, 1C’), we think it should be sufficient to just perform bacterial colony bisulfite sequencing analysis of *Rasgrf1* ICR to compare M^−^Z^+^ embryos with M^−^Z^−^ embryos in this study. Much higher levels of DNA methylation were obtained from female and male M^−^Z^+^ embryos than with their M^−^Z^−^ counterparts (Figures 3A, 3A’, 3B, 3B’). DNA methylation at the *Rasgrf1* ICR was lower in four female M^−^Z^−^ embryos, but it was lowest in three male M^−^Z^−^ embryos (Figures 3B, 3B’). Indeed, DNA methylation was significantly reduced at the *Rasgrf1* ICR in both female and male M^−^Z^−^ embryos compared with their M^−^Z^+^ counterparts (Figures 3C, 3C’). It was similar in the female and male M^−^Z^+^ embryos (Figure 3D). However, DNA methylation was significantly reduced at the *Rasgrf1* ICR in male M^−^Z^−^ embryos compared with that of female M^−^Z^−^ embryos (Figure 3E). Taken together, DNA methylation was more severely lost at the *Rasgrf1* ICR in male M^−^Z^−^ embryos than in female M^−^Z^-^ embryos (Figure 3, Supplementary Figure S1).

**FIGURE 4:**
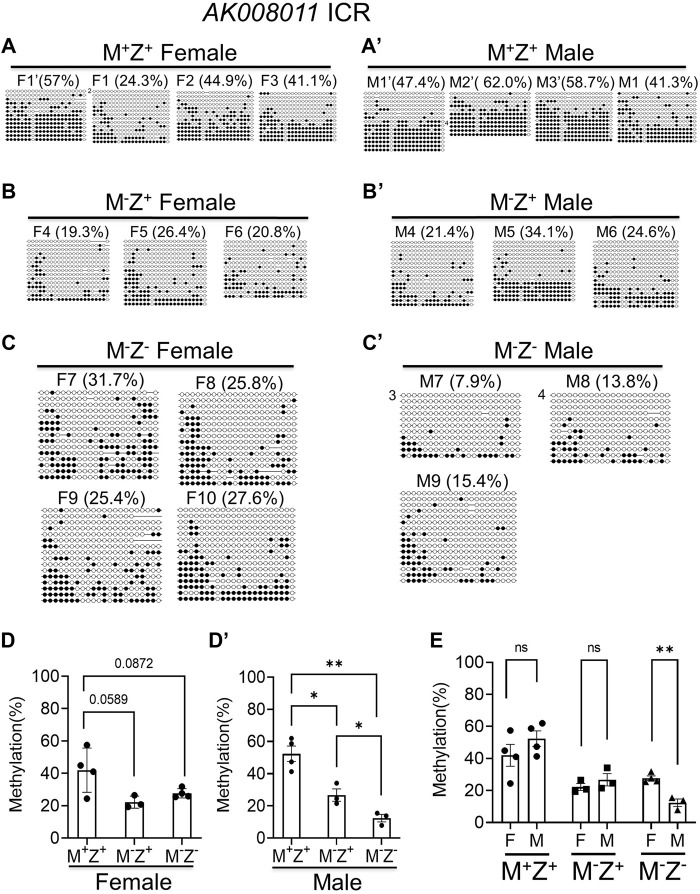
DNA methylation at the *AK008011* ICR was more significantly reduced in the male M^-^Z^-^ embryos compared with the female ones based on bacterial colony bisulfite sequencing analysis. Genomic DNA samples were isolated from at least three M^+^Z^+^, M^−^Z^+^, or M^−^Z^-^ E13.5 embryos of each gender that had been used for RNA-seq analyses in this study too. They were subjected to bisulfite mutagenesis followed by bacterial colony sequencing of the 605-bp bisulfite PCR product of the *AK008011* ICR region. The unconverted cytosine **(C)** residues were used to determine the unique clones for the bisulfite colony sequencing. Each row represents a unique clone, with the filled black circles for methylated CpG sites and unfilled white circles for unmethylated CpG sites. The number in front of a unique clone shows the number of sequenced non-unique clones containing the same sequence that cannot be distinguished by unconverted **(C)** residues. The percentage of DNA methylation in **(A,C’)** was calculated based on the number of methylated CpG sites divided by the total number of CpG sites for the sequenced unique clones of the bisulfite PCR product. Two-tailed Student’s *t* test was used for statistical analysis of DNA methylation level differences between two different genotypes of the same gender **(D,D’)** or within the same genotype between males and females **(E)**. Statistical significance: *, *p* < 0.05; **, *p* < 0.01; ns, not statistically significant. **(A’)** Four M^+^Z^+^ female embryos (F1’ and F1–F3). **(A’)** Four M^+^Z^+^ male embryos (M1’–M3’ and M1). **(B)** Three M^−^Z^+^ female embryos (F4–F6). **(B’)** Three M^−^Z^+^ male embryos (M4–M6). **(C)** Four M^−^Z^-^ female embryos (F7–F10). **(C’)** Three M^−^Z^-^ male embryos (M7–M9). **(D)** DNA methylation at the *AK008011* ICR was significantly reduced in four M^−^Z^-^ or three M^−^Z^+^ female embryos compared with four M^+^Z^+^ female embryos. **(D’)** DNA methylation at the *AK008011* ICR was significantly reduced in three M^−^Z^-^ male embryos compared with four M^+^Z^+^ or three M^−^Z^+^ male embryos. It was also reduced in three M^−^Z^+^ male embryos compared with four M^+^Z^+^ male embryos. **(E)** DNA methylation at the *AK008011* ICR was similar comparing M^+^Z^+^ or M^−^Z^+^ female embryos with their male counterparts. It was more significantly reduced in three M^−^Z^-^ male embryos than in four M^−^Z^-^ female embryos.

Similarly, we performed bacterial colony bisulfite sequencing analysis of the *AK008011* ICR in a set of M^+^Z^+^, M^−^Z^+^, and M^−^Z^−^ embryos that consisted of 3–4 female and male embryos for each genotype (Figure 4). DNA methylation was significantly reduced at the *AK008011* ICR in the male M^−^Z^−^ embryos compared with male M^+^Z^+^ or M^−^Z^+^ embryos, whereas it was close to being significantly reduced in the female M^−^Z^+^ or M^−^Z^−^ embryos compared with female M^+^Z^+^ embryos (Figures 4A, 4A’, 4B, 4B’, 4C, 4C’, 4D, 4D’). It was also significantly decreased in male M^−^Z^+^ embryos in comparison to male M^+^Z^+^ embryos (Figure 4D’). There was no significant difference in DNA methylation at the *AK008011* ICR comparing female M^+^Z^+^ or M^−^Z^+^ embryos with their male counterparts, respectively (Figures 4A, 4A’, 4B, 4B’, 4E). Interestingly, DNA methylation was more significantly reduced at the *AK008011* ICR in male M^−^Z^−^ embryos than in female M^−^Z^−^ embryos (Figure 4E). These results suggest that DNA methylation at the *AK008011* ICR was more significantly lost in the male M^−^Z^−^ embryos than in female M^−^Z^−^ embryos (Supplementary Figure S2). Therefore, *Zfp57* exhibited sexually dimorphic effect on DNA methylation at the *AK008011* ICR in M^−^Z^−^ embryos lacking both maternal and zygotic *Zfp57*.

We examined DNA methylation at few other known ICRs. As expected, DNA methylation was similarly lost at the ICRs of *Inpp5f*, *Zac1*, and IG-DMR in the female and male M^−^Z^-^ mutant embryos based on the methylation IGV plots (Supplementary Figure S2). Therefore, it seemed that loss of ZFP57 caused more severe loss of DNA methylation imprint at three ICRs in the male M^−^Z^−^ embryos than in the female M^−^Z^-^ embryos, with statistical significance observed at the *Rasgrf1* and *AK008011* ICRs.

### Loss of Maternal *Zfp57* Caused Partial Loss of DNA Methylation Imprint at a Few Imprinting Control Regions

**FIGURE 5:**
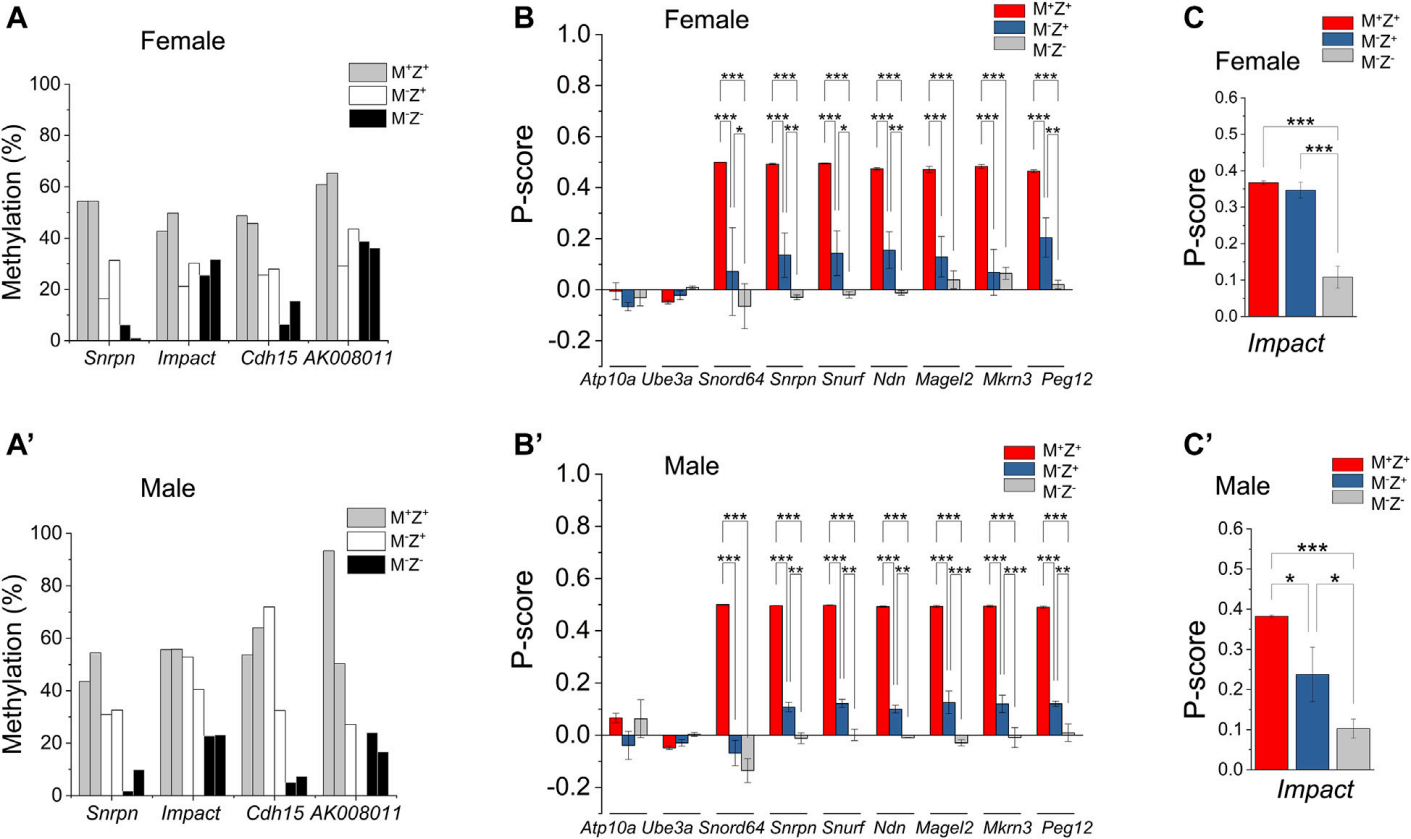
Maternal effect of *Zfp57* was observed on DNA methylation imprint in a few imprinted regions as well as on the expression of a few imprinted genes. Genomic DNA samples were isolated from the *Zfp57* ^*+/−*^ (M^+^Z^+^), *Zfp57* ^*−/+*^ (M^−^Z^+^), and *Zfp57* ^*−/−*^ (M^−^Z^-^) hybrid 129/DBA E13.5 embryos and subjected to whole-genome bisulfite sequencing (WGBS) analysis (Jiang et al., 2021). Two female embryos of each genotype and two male embryos of each genotype were used in this study for examination of DNA methylation imprint at the imprinted regions. For RNA-seq analysis, at least three RNA samples were used for each genotype (M^+^Z^+^ *,* M^−^Z^+^, and M^−^Z^-^) of the female or male embryos. **(A,A’)** DNA methylation at the *Snrpn*, *Impact*, *Cdh15*, and *AK008011* ICRs in the female embryos **(A)** or male embryos **(A’)**. **(B,B’)** P-score was calculated to measure the expression of the paternal alleles of the imprinted genes at the *Snrpn* imprinted region in the female embryos **(B)** or male embryos **(B’)**. These include the *Atp10a*, *Ube3a*, *Snord64*, *Snrpn*, *Ndn*, *Magel2*, *Mkrn3*, and *Peg12* imprinted genes at the *Snrpn* imprinted region. Two-way ANOVA was used for statistical analysis of the P-score differences of each imprinted gene in the female M^+^Z^+^ *,* M^−^Z^+^, and M^−^Z^-^ or male M^+^Z^+^ *,* M^−^Z^+^, and M^−^Z^-^ embryos. Statistical significance: *, *p* < 0.05; **, *p* < 0.01; and ***, *p* < 0.001. **(C,C’)** P-score was calculated to measure the paternal allele expression of *Impact* in the female embryos **(C)** or male embryos **(C’)**. One-way ANOVA was used for statistical analysis of the P-score differences of *Impact* in the female or male embryos. Statistical significance: * *p* < 0.05; ** *p* < 0.01; and ***, *p* < 0.001. Please refer to *Materials and Methods* for the definition of P-score and its calculation equation.

DNA methylation appeared to be partially lost at a few ICRs in female M^−^Z^+^ or male M^−^Z^+^ embryos (Supplementary Figures 5A, 5A’). This caused partial allelic expression switch of many imprinted genes at the *Snrpn* imprinted region as well as *Impact* in male M^−^Z^+^ embryos, which is described in more detail below (Figures 5B, 5B’). Indeed, DNA methylation was partially lost at the *Snrpn* and *Cdh15* ICRs in female M^−^Z^+^ embryos, although it was almost completely lost at these two ICRs in female M^−^Z^−^ embryos (Figure 5A). One female M^−^Z^+^ embryo had more severe loss of DNA methylation at the *Snrpn* ICR than the other female M^−^Z^+^ embryo. DNA methylation was similarly partially lost at the *Impact* and *AK008011* ICRs in female M^−^Z^+^ embryos as well as in female M^−^Z^−^ embryos (Figure 5A). Similar results were obtained in bacterial colony bisulfite sequencing analysis for the *AK008011* ICR (Figures 4A–4C). Indeed, these observations are confirmed by statistical analyses (Figure 4D, Supplementary Figures S1D–S1E). DNA methylation was significantly reduced at these four ICRs in the female M^−^Z^+^ embryos compared with those of the female M^+^Z^+^ embryos (Supplementary Figures S1D–S1E). Furthermore, it was more significantly or close to significantly reduced at the *Snrpn* and *Cdh15* ICRs, but not at the *Impact* and *AK008011* ICRs, while comparing the female M^−^Z^−^ embryos with the female M^−^Z^+^ embryos (Supplementary Figures S1D–S1E). These results suggest that both maternal and zygotic *Zfp57* are necessary but partially redundant for the maintenance of DNA methylation imprint at the *Snrpn* and *Cdh15* ICRs in female embryos. However, only maternal *Zfp57*, but not zygotic *Zfp57*, is required for maintaining DNA methylation imprint at the *Impact* and *AK008011* ICRs in female embryos.

DNA methylation was also partially lost at the *Snrpn* ICR in both male M^−^Z^+^ embryos (Figure 5A’). It was partially lost at the *Cdh15* ICR in one but not in the other male M^−^Z^+^ embryo (Figure 5A’). DNA methylation was partially lost at the *Impact* ICR in male M^−^Z^−^ embryos, but it was largely intact at the *Impact* ICR in male M^−^Z^+^ embryos (Figure 5A’). DNA methylation was partially lost at the *AK008011* ICR in one male M^−^Z^+^ embryo, which was similar to loss of DNA methylation at the *AK008011* ICR in both male M^−^Z^−^ embryos (Figure 5A’). Unfortunately, there were no sequence reads at the *AK008011* ICR in the other male M^−^Z^+^ embryo and, therefore, we could not determine if DNA methylation was similarly lost at the *AK008011* ICR in that male M^−^Z^+^ embryo based on WGBS. These results were also confirmed by statistical analyses (Supplementary Figures S1D’–S1E’). DNA methylation was significantly reduced at the *AK008011* ICR in male M^−^Z^+^ embryos compared with male M^+^Z^+^ embryos in bacterial colony bisulfite sequencing analysis, and it was even more significantly reduced in male M^−^Z^−^ embryos than in male M^−^Z^+^ embryos (Figures 4A’–D’). DNA methylation was close to significantly reduced at the *Snrpn* ICR in the male M^−^Z^+^ embryos compared with the male M^+^Z^+^ embryos, but even more significantly reduced in the male M^−^Z^−^ embryos than in the male M^+^Z^+^ or M^−^Z^+^ embryos (Supplementary Figure S1E’). DNA methylation was only significantly reduced at the *Cdh15* ICR in the male M^−^Z^-^ embryos versus male M^+^Z^+^ embryos, whereas it was significantly reduced at the *Impact* ICR in the male M^−^Z^−^ embryos compared with that in the male M^+^Z^+^ or M^−^Z^+^ embryos (Supplementary Figure S1E’).

Actually, DNA methylation was more severely lost at the *AK008011* ICR in male M^−^Z^−^ embryos compared with that in female M^−^Z^−^ embryos (Figures 4E, 5A, 5A’, Supplementary Figure S1C). It seems that maternal *Zfp57* is required for maintaining DNA methylation imprint at the *AK008011* ICR in both female and male embryos, whereas zygotic *Zfp57* seemed to contribute to maintenance of DNA methylation at the *AK008011* ICR in male but not female embryos. Both maternal and zygotic *Zfp57* play partially redundant roles in maintaining DNA methylation at the *Impact* ICR in male embryos, although only maternal but not zygotic *Zfp57* is involved in maintaining DNA methylation at the *Impact* ICR in female embryos (Figure 5A, 5A’, Supplementary Figure S1C). These are also caused by the gender-specific effect of *Zfp57* on ICR DNA methylation.

**FIGURE 6:**
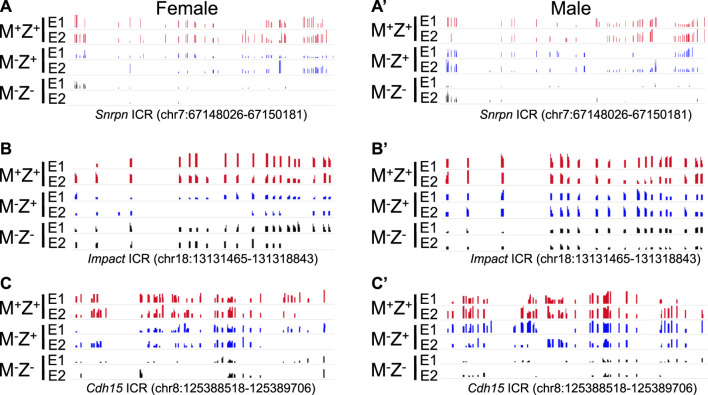
DNA methylation was partially lost at the *Snrpn*, *Impact*, and *Cdh15* ICRs in the female or male embryos lacking just maternal *Zfp57* on the methylation IGV plot. After WGBS analysis of the genomic DNA samples isolated from *Zfp57* ^*+/+*^ (M^+^Z^+^), *Zfp57* ^*−/+*^ (M^−^Z^+^), and *Zfp57* ^*−/−*^ (M^−^Z^-^) hybrid 129/DBA embryos, the methylation IGV plots with the scale of 0–1 are shown for the *Snrpn*, *Impact*, and *Cdh15* ICRs in two female embryos (E1 and E2) or two male embryos (E1 and E2) for each of the M^+^Z^+^, M^−^Z^+^, and M^−^Z^-^ genotypes. DNA methylation imprint was partially lost at these three imprinted regions in the M^−^Z^+^ embryos, although more severe loss of DNA methylation was observed in the M^−^Z^-^ embryos. **(A,A’)** Methylation IGV plot of the *Snrpn* ICR (mm9, chr7:67,148,026–67,150,181) in the female **(A)** or male **(A’)** embryos. **(B,B’)** Methylation IGV plot of the *Impact* ICR (mm9, chr18: 13,131,465–1,31,318,843) in the female **(B)** or male **(B’)** embryos. **(C,C’)** Methylation IGV plot of the *Cdh15* ICR (mm9, chr8:125,388,518–125,389,706) in the female **(C)** or male **(C’)** embryos.

Loss of DNA methylation at these ICRs in female M^−^Z^+^ and M^−^Z^−^ embryos or male M^−^Z^+^ and M^−^Z^−^ embryos is also clearly seen on the methylation IGV plots (Figures 2, 6). DNA methylation was similarly partially lost at the *AK008011* ICR in female and male M^−^Z^+^ embryos as well as in female M^−^Z^−^ embryos, with more severe loss observed in the male M^−^Z^−^ embryos than in the female ones (Figures 2C, 2C’). Partial loss of DNA methylation was observed at the *Snrpn* ICR in female M^−^Z^+^ and male M^−^Z^+^ embryos on the methylation IGV plots, although DNA methylation was almost completely missing at the *Snrpn* ICR in female M^−^Z^−^ and male M^−^Z^−^ embryos (Figures 6A, 6A’). Partial loss of DNA methylation was observed at the *Impact* ICR in male M^−^Z^−^, but not male M^−^Z^+^ embryos, whereas DNA methylation was partially lost at the *Impact* ICR in female M^−^Z^+^ as well as in female M^−^Z^−^ embryos on the methylation IGV plots (Figure 6B, 6B’). Germline-derived DNA methylation imprint at the *Snrpn* and *Impact* ICRs was present on the maternal chromosomes in M^+^Z^+^ embryos as shown in the allelic methylation IGV plots (Supplementary Figure S3, Supplementary Table S1, S2). Indeed, loss of DNA methylation imprint occurred at the maternal *Snrpn* ICR in M^−^Z^+^ and M^−^Z^−^ embryos based on the allelic methylation IGV plots (Supplementary Figure S3A-S3A′). DNA methylation was also lost at the maternal *Impact* ICR in the female M^−^Z^+^ and M^−^Z^−^ embryos and in male M^−^Z^−^ embryos based on the allelic methylation IGV plots (Supplementary Figure S3B-S3B′). The allelic methylation results further confirm what has been observed on the methylation IGV plots of the *Snrpn* and *Impact* ICRs (Figure 6, Supplementary Figure S3A). Partial loss of DNA methylation was observed at the *Cdh15* ICR in both female M^−^Z^+^ embryos and one male M^−^Z^+^ embryo (Figures 6C, 6C’). DNA methylation was almost completely lost at the *Cdh15* ICR in female M^−^Z^−^ and male M^−^Z^−^ embryos compared with M^+^Z^+^ embryos on the methylation IGV plots (Figures 6C, 6C’).

### Loss of Just Maternal *Zfp57* Causes Allelic Expression Switch of Some Imprinted Genes

**FIGURE 7:**
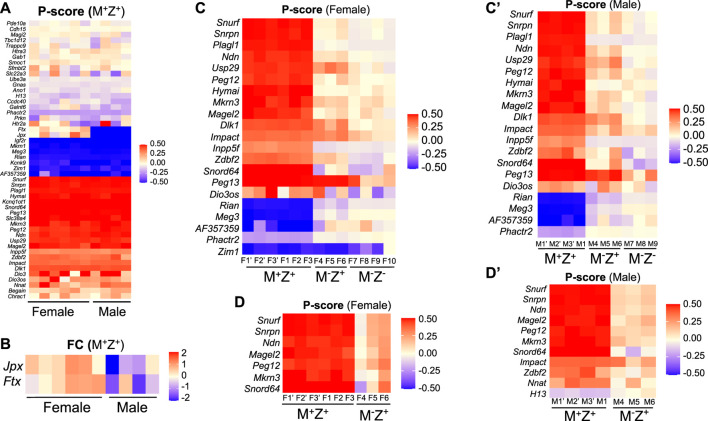
Allelic expression of some imprinted genes was primarily regulated by maternal *Zfp57*, and it may be affected differently in the female or male embryos lacking *Zfp57*. RNA-seq analysis was performed for 3–4 female 129/DBA hybrid embryos of the M^+^Z^+^, M^−^Z^+^, and M^−^Z^-^ genotypes and three male 129/DBA hybrid embryos of the M^+^Z^+^, M^−^Z^+^, and M^−^Z^-^ genotypes. P-score was calculated for assessing the proportion of the transcripts derived from the paternal allele of each imprinted gene carrying an SNP, and the intensity difference heatmaps were plotted accordingly ranging from 0.5 (red, paternal allele only) to −0.5 (blue, maternal allele only) Panel **(A,C,C’,D,D’)**. Please refer to *Materials and Methods* for the P-score calculation and generation of intensity difference heatmaps. The Kruskal–Wallis test was used for statistical significance comparisons of the P-score differences of each imprinted gene across three different genotypes of the same gender, and then intensity difference heatmaps were generated for the imprinted genes containing the P-score difference above the threshold (∆P-Score≥ 0.1, *p* < 0.05) between M^−^Z^-^ and M^+^Z^+^ embryos **(C,C’)** or between M^−^Z^+^ and M^+^Z^+^ embryos **(D,D’)**. **(A)** P-score was generated and compared for the transcripts of 48 known imprinted genes with an exonic SNP between six female M^+^Z^+^ and four male M^+^Z^+^ embryos on an intensity difference heatmap. Red, paternal allele expression only with a P-score of 0.5; blue, maternal allele expression only with a P-score of −0.5. Most imprinted genes on the top of the panel are singleton ones with a P-score close to zero indicating biallelic expression. *Jpx* and *Ftx* located on the X chromosome exhibited almost biallelic expression in the female M^+^Z^+^ embryos and exclusively maternal allele expression in the male M^+^Z^+^ embryos as expected. They are good internal positive controls for our data analyses. **(B)** DESeq2 was used to analyze the RNA-seq data to sort out 105 imprinted genes with the TPM values above the threshold (TPM>1) in at least one embryo, out of 148 known mouse imprinted genes listed on the geneimprint website (www.geneimprint.com). Among 105 imprinted genes, only *Jpx* and *Ftx* showed significant differences, with |log_2_FC |> 0.3 (*p* < 0.05) as the threshold of significant difference, in their expression level comparison of six female M^+^Z^+^ embryos with four male M^+^Z^+^ embryos. The intensity difference heatmaps were generated for these two imprinted genes afterward. **(C)** Intensity difference heatmaps were shown for 21 imprinted genes in six M^+^Z^+^ (F1’–F3’, F1–F3), three M^−^Z^+^ (F4–F6), and four M^−^Z^-^ (F7–F10) female embryos with a statistically different P-score (∆P-Score≥ 0.1, *p* < 0.05) comparing M^−^Z^-^ with M^+^Z^+^ embryos. **(C’)** Intensity difference heatmaps were shown for 20 imprinted genes in four M^+^Z^+^(M1’–M3’, M1), three M^−^Z^+^ (M4–M6), and three M^−^Z^-^ (M7–M9) male embryos with a statistically different P-score (∆P-Score≥ 0.1, *p* < 0.05) comparing M^−^Z^-^ with M^+^Z^+^ embryos. **(D)** Allelic expression was significantly different (∆P-Score≥ 0.1, *p* < 0.05) for seven imprinted genes in the comparison of three female M^−^Z^+^ (F4–F6) with six female M^+^Z^+^ (F1’–F3’, F1–F3) embryos. **(D’)** Eleven imprinted genes exhibited statistically significantly different P-scores (∆P-Score≥ 0.1, *p* < 0.05) in the comparison of three male M^−^Z^+^ (M4–M6) with four male M^+^Z^+^ (M1’–M3’, M1) embryos.

Since DNA methylation imprint was partially lost at a few ICRs in M^−^Z^+^ embryos, we wonder if this may have any effect on the expression of the corresponding imprinted genes at these ICRs. RNA-seq analyses were carried out with another set of mouse embryos to examine expression of imprinted genes in the female M^+^Z^+^, M^−^Z^+^, and M^−^Z^−^ 129/DBA hybrid E13.5 embryos and male M^+^Z^+^, M^−^Z^+^, and M^−^Z^-^ 129/DBA hybrid E13.5 embryos, with at least three embryos in each group. These mouse embryos were different from the ones used for WGBS in the previous study. We used P-score for measuring the proportion of the transcripts expressed from the paternal allele of each imprinted gene (Figure 7, Supplementary Table S3–S4, *Materials and Methods*). Since there are no exonic SNPs on many imprinted genes, we could not analyze their P-score differences in these embryos. *Ftx* and *Jpx* were only two out of 48 known imprinted genes with an exonic SNP that showed significant P-score difference in statistical analysis (∆P-Score≥ 0.1, *p* < 0.05) comparing six female M^+^Z^+^ embryos with four male ones (Figure 7A). As expected, X-linked *Ftx* and *Jpx*, which are involved in X chromosome inactivation in the female embryos, along with *Xist* and *Tsix*, exhibited almost biallelic expression in the female M^+^Z^+^ embryos, whereas they were exclusively expressed from the maternal allele on the X chromosome in the male M^+^Z^+^ embryos (Figure 7A) (Collombet et al., 2020; Patrat et al., 2020). Thus, *Ftx* and *Jpx* serve as good internal positive controls for our data analyses. They were also the only two imprinted genes that displayed significant differences in the expression levels comparing six female M^+^Z^+^ embryos with four male ones that will be discussed further below (Figure 7B).

The intensity difference heatmaps were generated for 21 imprinted genes with statistically different P-score (∆P-Score≥ 0.1, *p* < 0.05) by comparing the female M^−^Z^−^ embryos with the female M^+^Z^+^ embryos and for 20 imprinted genes (∆P-Score≥ 0.1, *p* < 0.05) when the male M^−^Z^−^ embryos were compared with the male M^+^Z^+^ embryos (Table 1, Figure 7C, 7C’). *Zim1* at the *Peg3* imprinted region was maternally expressed in female and male M^+^Z^+^ embryos or male M^−^Z^−^ embryos, but it became partially biallelic in female M^−^Z^−^ embryos (Supplementary Figure S4A).

**TABLE 1:** Allelic expression switch was observed for some imprinted genes lacking *Zfp57*.

Imprinted gene	Female M^+^Z^+^	Female M^−^Z^-^	Male M^+^Z^+^	Male M^−^Z^-^
*Snurf*	Paternal (P)	Bi-allelic	Paternal (P)	Bi-allelic
*Snrpn*	Paternal (P)	Bi-allelic	Paternal (P)	Bi-allelic
*Zac1* (*Plagl1*)	Paternal (P)	Bi-allelic	Paternal (P)	Bi-allelic
*Ndn*	Paternal (P)	Bi-allelic	Paternal (P)	Bi-allelic
*Usp29*	Paternal (P)	Bi-allelic	Paternal (P)	Bi-allelic
*Peg12*	Paternal (P)	Bi-allelic	Paternal (P)	Bi-allelic
*Hymai*	Paternal (P)	Bi-allelic	Paternal (P)	Bi-allelic
*Mkrn3*	Paternal (P)	Bi-allelic	Paternal (P)	Bi-allelic
*Magel2*	Paternal (P)	Bi-allelic	Paternal (P)	Bi-allelic
*Dlk1*	Paternal (P)	Bi-allelic	Paternal (P)	Bi-allelic
*Impact*	Preferential Paternal (P)	Slightly Paternal (P)	Preferential Paternal (P)	Slightly Paternal (P)
*Inpp5f*	Preferential Paternal (P)	Slightly Maternal (M)	Preferential Paternal (P)	Slightly Maternal (M)
*Zdbf2*	Preferential Paternal (P)	Slightly Maternal (M)	Preferential Paternal (P)	Slightly Maternal (M)
*Snord64*	Paternal (P)	Slightly Maternal (M)	Paternal (P)	Preferential Maternal (M)
*Peg13*	Paternal (P)	Slightly Paternal (P)	Paternal (P)	Slightly Paternal (P)
*Dio3os*	Preferential Paternal (P)	Preferential Maternal (M)	Preferential Paternal (P)	Bi-allelic
*Rian*	Maternal (M)	Bi-allelic	Maternal (M)	Bi-allelic
*Meg3*	Maternal (M)	Bi-allelic	Maternal (M)	Bi-allelic
*AF357359*	Maternal (M)	Bi-allelic	Maternal (M)	Bi-allelic
*Phactr2*	Preferential Maternal (M)	Bi-allelic	Preferential Maternal (M)	Bi-allelic
*Zim1*	Maternal (M)	Preferential Maternal (M)	Maternal (M)	^a^Maternal (M) (ΔP-score = 0.0468, p = 0.480)

Note: The Kruskal–Wallis test was used for statistical significance comparisons of the P-score differences of each imprinted gene across three different genotypes of the same gender.

a P-score of *Zim1* in the male M^−^Z^−^ embryos was compared with that in the male M^+^Z^+^ embryos to generate the P-score difference (∆P-score = 0.0468, *p* = 0.480).

Similarly, the intensity difference heatmaps (∆P-Score≥ 0.1, *p* < 0.05) were generated for seven imprinted genes by comparing three female M^−^Z^+^ embryos with six female M^+^Z^+^ embryos and for 11 imprinted genes when three male M^−^Z^+^ embryos were compared with four male M^+^Z^+^ embryos (Figures 7D, 7D’). Indeed, allelic expression of the imprinted genes at the *Snrpn* imprinted region was mostly affected in female or male M^−^Z^+^ embryos as well as in female or male M^−^Z^−^ embryos compared with female or male M^+^Z^+^ embryos of the same gender (Figures 5B, 5B’, 7, Supplementary Table S3–S4). *Snurf*, *Snrpn*, *Ndn*, *Magel2*, *Peg12*, *Mkrn3*, and *Snord64* at the *Snrpn* imprinted region were all paternally expressed in female and male M^+^Z^+^ embryos. They became biallelic in female or male M^−^Z^−^ embryos, except that *Snord64* was slightly preferentially maternally expressed in male M^−^Z^−^ embryos (Figures 5B, 5B’, 7, Supplementary Table S3–S4). They were largely biallelic in female or male M^−^Z^+^ embryos at variable levels. Consistent with their tissue-specific or species-specific imprinting phenomena, as well as our previous results in mouse embryos, *Atp10a* and *Ube3a* at the *Snrpn* imprinted region were almost biallelic in the female M^+^Z^+^, M^−^Z^+^, and M^−^Z^−^ embryos or male M^+^Z^+^, M^−^Z^+^, and M^−^Z^−^ embryos (Figures 5B, 5B’, Supplementary Table S3-S4) (DuBose et al., 2010; Hsiao et al., 2019; Jiang et al., 2021).

*Impact* was preferentially paternally expressed in female and male M^+^Z^+^ embryos (Figure 5C-5C’, Figure 7C-7C’, Supplementary Table S3–S4). It became almost biallelic in female or male M^−^Z^−^ embryos and partially biallelic in male M^−^Z^+^ embryos, whereas it remained preferentially paternally expressed in female M^−^Z^+^ embryos (Figure 5C-5C’, Figure 7C-7C’, 7D’). *Zdbf2* and *Nnat* (*Peg5*) were preferentially paternally expressed in female or male M^+^Z^+^ embryos (Supplementary Figure S4, Supplementary Table S3-S4). *Zdbf2* was partially biallelic in female M^−^Z^+^ embryos and almost completely biallelic in male M^−^Z^+^ embryos (Supplementary Figure S4B-S4B'). *Nnat* (*Peg5*) remained preferentially paternally expressed in female M^−^Z^+^ embryos, but it was almost completely biallelic in male M^−^Z^+^ embryos (Supplementary Figure S4C-S4C'). Slightly preferential maternal expression was observed for *H13* in female and male M^+^Z^+^ embryos or female M^−^Z^+^ embryos (Supplementary Figure S4D-S4D'). *H13* was biallelic in male M^−^Z^+^ embryos (Supplementary Figure S4D'). Taken together, loss of maternal *Zfp57* caused variable allelic expression switch of *Impact*, *Zdbf2*, *Nnat* (*Peg5*), and *H13* in the male M^−^Z^+^ embryos, whereas it only had a minor effect on allelic expression of *Zdbf2* in female M^−^Z^+^ embryos. The gender effects on allelic expression of these imprinted genes will be further discussed below.

### Expression Levels of Some Imprinted Genes Were Regulated by Maternal *Zfp57*

We also analyzed expression levels of some imprinted genes based on RNA-seq results. Among 105 analyzed imprinted genes with the TPM values above the threshold (see *Materials and Methods*), only two imprinted genes (*Jpx* and *Ftx*) had statistically significant differences (|log_2_FC |> 0.3, *p* < 0.05) in gene expression levels comparing the female M^+^Z^+^ embryos with the male ones (Figure 7B). Since they are involved in X chromosome inactivation only in the females (Furlan et al., 2018; Collombet et al., 2020; Patrat et al., 2020), it is expected that the expression of *Jpx* and *Ftx* was higher in the female M^+^Z^+^ embryos than in the male ones (Figure 7B).

**FIGURE 8:**
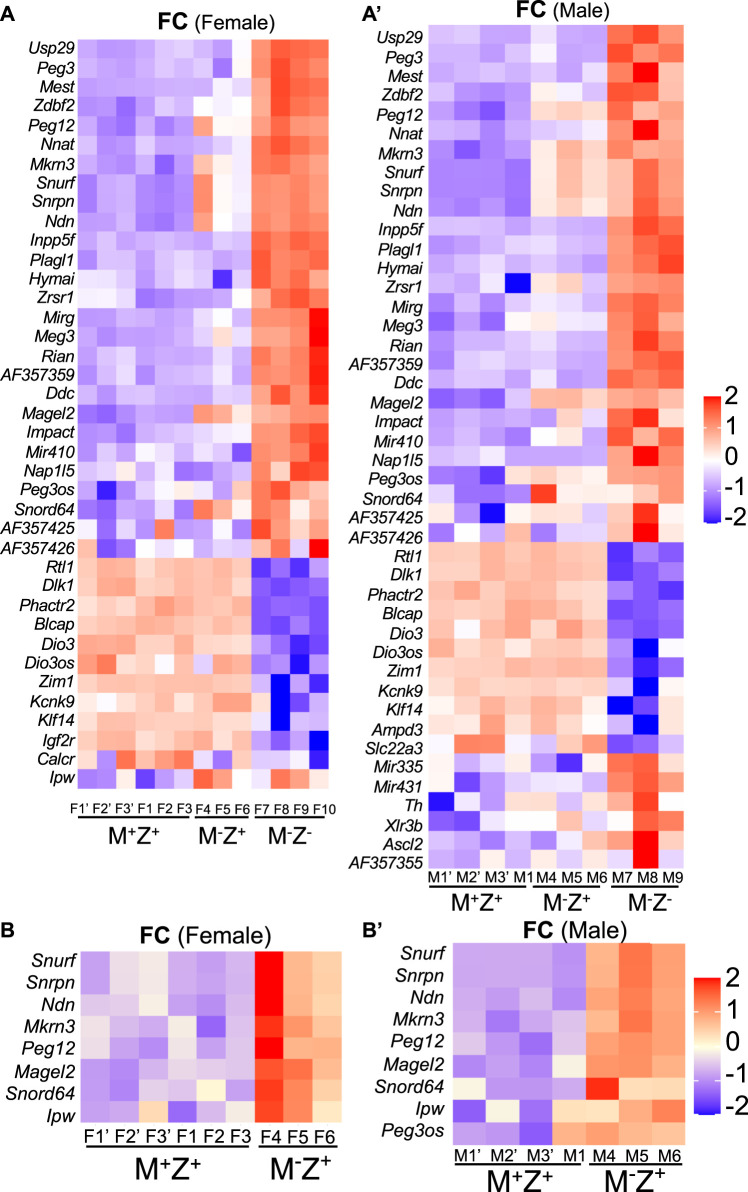
Some imprinted genes displayed sexually dimorphic effects in their expression in mouse embryos upon loss of *Zfp57* or just maternal *Zfp57*. RNA-seq analysis was performed for female and male 129/DBA hybrid embryos of the M^+^Z^+^, M^−^Z^+^, and M^−^Z^-^ genotypes. The log_2_FC values were used for quantification of the fold change (FC) of gene expression based on the ratios of TPM values for the imprinted gene transcripts in the M^+^Z^+^, M^−^Z^+^, and M^−^Z^-^ embryos of the same gender. The imprinted genes with significant difference in their expression levels above the threshold (|log_2_FC |> 0.5, *p* < 0.05) were first identified with DESeq2 by comparing the RNA-seq data of female or male M^−^Z^-^ embryos with those of the M^+^Z^+^ embryos of the same gender. Then, these imprinted genes were used for making the intensity difference heatmaps in the female M^+^Z^+^, M^−^Z^+^, and M^−^Z^-^ embryos or male M^+^Z^+^, M^−^Z^+^, and M^−^Z^-^ embryos. **(A)** Intensity difference heatmaps were generated for 39 imprinted genes in six M^+^Z^+^(F1’–F3’ and F1–F3), three M^−^Z^+^ (F4–F6) and four M^−^Z^-^ (F7–F10) female embryos that displayed significantly different expression levels between the female M^−^Z^-^ and female M^+^Z^+^ embryos. **(A’)** Intensity difference heatmaps were generated for 44 imprinted genes in four M^+^Z^+^(M1’–M3’ and M1), three M^−^Z^+^ (M4–M6), and three M^−^Z^-^ (M7–M9) male embryos that displayed significantly different expression levels between the male M^−^Z^-^ and male M^+^Z^+^ embryos. **(B)** Intensity difference heatmaps were shown for eight imprinted genes with significantly different expression levels in the comparison of three female M^−^Z^+^ (F4–F6) with six female M^+^Z^+^ (F1’–F3’ and F1–F3) embryos. **(B’)** Intensity difference heatmaps were shown for nine imprinted genes with significantly different expression levels in the comparison of three male M^−^Z^+^ (M4–M6) with four male M^+^Z^+^ (M1’–M3’ and M1) embryos.

The intensity difference heatmaps were generated for 39 imprinted genes with statistically significant changes (|log_2_FC |> 0.5, *p* < 0.05) in their expression levels when the female M^−^Z^−^ embryos were compared with the female M^+^Z^+^ embryos and for 44 imprinted genes when the male M^−^Z^−^ embryos were compared with the male M^+^Z^+^ embryos (Table 2; Figure 8A, 8A’). Similarly, the intensity difference heatmaps were generated for eight imprinted genes in the comparison of the female M^−^Z^+^ embryos with the female M^+^Z^+^ embryos and for nine imprinted genes when the male M^−^Z^+^ embryos were compared with the male M^+^Z^+^ embryos (Table 3, Figure 8B, 8B’). Eight imprinted genes at the *Snrpn* imprinted region were differentially expressed either in the comparison of the female M^−^Z^+^ embryos with the female M^+^Z^+^ embryos or in the comparison of the male M^−^Z^+^ embryos with the male M^+^Z^+^ embryos (Figure 8B, 8B’).

**TABLE 2:** Expression levels of these imprinted genes were significantly different in either the female or male M^−^Z^-^ embryos compared with the M^+^Z^+^ embryos of the same gender.

Imprinted gene	^a^Female M^−^Z^-^ vs. M^+^Z^+^	^b^ Male M^−^Z^-^ vs. M^+^Z^+^
*Usp29*	Increased	Increased
*Peg3*	Increased	Increased
*Peg1 (Mest)*	Increased	Increased
*Zdbf2*	Increased	Increased
*Peg12*	Increased	Increased
*Nnat (Peg5)*	Increased	Increased
*Mkrn3*	Increased	Increased
*Snurf*	Increased	Increased
*Snrpn*	Increased	Increased
*Ndn*	Increased	Increased
*Inpp5f*	Increased	Increased
*Zac1* (*Plagl1*)	Increased	Increased
*Hymai*	Increased	Increased
*Zrsr1*	Increased	Increased
*Mirg*	Increased	Increased
*Meg3*	Increased	Increased
*Rian*	Increased	Increased
*AF357359*	Increased	Increased
*Ddc*	Increased	Increased
*Magel2*	Increased	Increased
*Impact*	Increased	Increased
*Mir410*	Increased	Increased
*Nap1l5*	Increased	Increased
*Peg3os*	Increased	Increased
*Snord64*	Increased	Increased
*AF357425*	Increased	Increased
*AF357426*	Increased	Increased
*Rtl1*	Decreased	Decreased
*Dlk1*	Decreased	Decreased
*Phactr2*	Decreased	Decreased
*Blcap*	Decreased	Decreased
*Dio3*	Decreased	Decreased
*Dio3os*	Decreased	Decreased
*Zim1*	Decreased	Decreased
*Kcnk9*	Decreased	Decreased
*Klf14*	Decreased	Decreased
*Igf2r*	Decreased	Log_2_FC= - 0.307 p=0.011
*Calcr*	Decreased	Log_2_FC= -0.235 p=0.494
*Ipw*	Increased	Log_2_FC =0.877 p=0.064
*Ampd3*	Log_2_FC = -0.447 p=0.013	Decreased
*Slc22a3*	Log_2_FC= -0.096 p=0.640	Decreased
*Mir335*	Log_2_FC= 0.522 p=0.137	Increased
*Mir431*	Log_2_FC= 0.077 p=0.863	Increased
*Th*	Log_2_FC= 0.066 p=0.631	Increased
*Xlr3b*	Log_2_FC = 0.127 p=0.840	Increased
*Ascl2*	Log_2_FC =0.299 p= 0.642	Increased
*AF357355*	Log_2_FC = 0.433 p=0.260	Increased

Note: The imprinted genes with significant difference in their expression levels above the threshold (|log_2_FC |> 0.5, *p* < 0.05) were identified with DESeq2 by comparing their expression levels in the female or male M^−^Z^-^ embryos with those of the M^+^Z^+^ embryos of the same gender. FC, fold change of the expression levels of the imprinted gene.

a Expression levels of the imprinted genes in the female M^−^Z^-^ embryos were compared with those in the female M^+^Z^+^ embryos.

b Expression levels of the imprinted genes in the male M^−^Z^-^ embryos were compared with those in the male M^+^Z^+^ embryos.

**TABLE 3:** These imprinted genes showed significant difference in their expression levels in the female or male M^−^Z^+^ embryos compared with those in the female or male M^+^Z^+^ embryos of the same gender.

Imprinted gene	^a^ Female M^−^Z^+^ vs. M^+^Z^+^	^b^ Male M^−^Z^+^ vs. M^+^Z^+^
*Snurf*	Increased	Increased
*Snrpn*	Increased	Increased
*Ndn*	Increased	Increased
*Mkrn3*	Increased	Increased
*Peg12*	Increased	Increased
*Magel2*	Increased	Increased
*Snord64*	Increased	Increased
*Ipw*	Increased	Increased
*Peg3os*	Log_2_FC = 0.311 *p* = 0.234	Increased

Note: The imprinted genes with significant difference in their expression levels above the threshold (|log_2_FC |> 0.5, *p* < 0.05) were identified with DESeq2 by comparing their expression levels in the female or male M^−^Z^+^ embryos with those of the M^+^Z^+^ embryos of the same gender. FC, fold change of the expression levels of the imprinted gene.

a Expression levels of the imprinted genes in the female M^−^Z^+^ embryos were compared with those in the female M^+^Z^+^ embryos.

b Expression levels of the imprinted genes in the male M^−^Z^+^ embryos were compared with those in the male M^+^Z^+^ embryos.

*Peg3os* was significantly increased in male M^−^Z^+^ embryos compared with male M^+^Z^+^ embryos (Figure 8B’). *Impact* was significantly increased in female M^−^Z^+^ embryos compared with female M^+^Z^+^ embryos (Supplementary Figure S5B-S6B', Supplementary Table S5-S6). There was not much change in the expression levels of *Cdh15* in female M^−^Z^+^ and M^−^Z^-^ embryos or male M^−^Z^+^ and M^−^Z^−^ embryos compared with M^+^Z^+^ embryos (Supplementary Figure S5C-S6C', Supplementary Table S5-S6). *Zrsr1* was also significantly increased in male M^−^Z^+^ embryos compared with male M^+^Z^+^ embryos (Supplementary Figure S8A-S8A', see below).

Taken together, loss of maternal *Zfp57* caused increased expression of eight imprinted genes at the *Snrpn* imprinted region in both female and male M^−^Z^+^ embryos. It also resulted in increased expression of *Peg3os* and *Zrsr1* in male M^−^Z^+^ embryos, as well as increased expression of *Impact* in female M^−^Z^+^ embryos.

### Gender Effect on Allelic Expression of the Imprinted Genes Upon Loss of *Zfp57*

**FIGURE 9:**
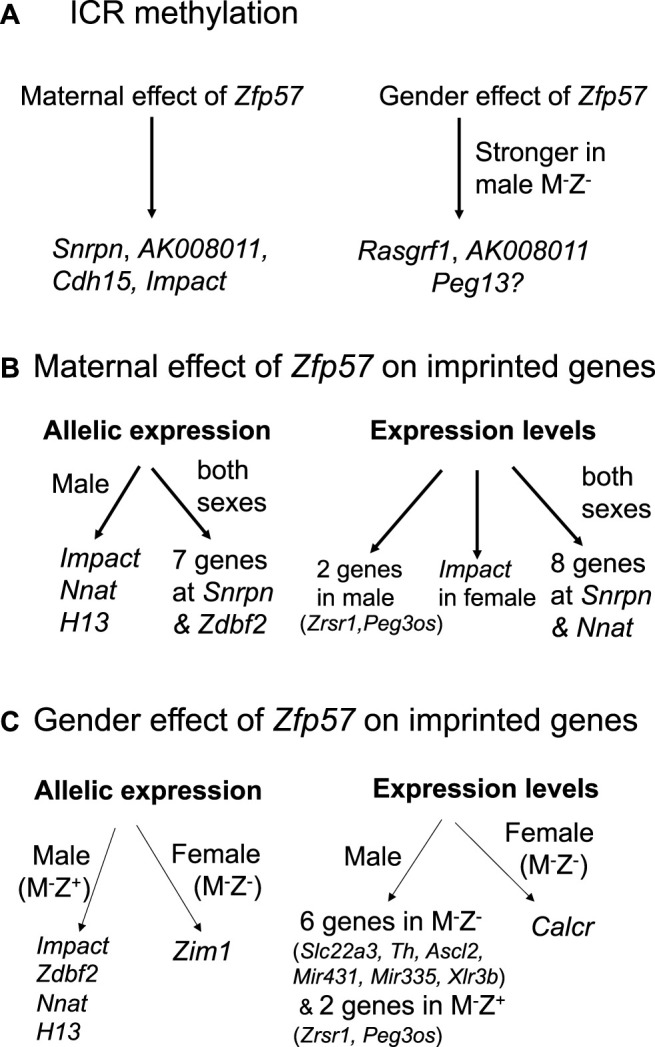
*Zfp57* exhibits maternal effect and gender-specific effect in the maintenance of DNA methylation imprint and expression of the imprinted genes. The diagrams are shown for the maternal effect and gender-specific effect of *Zfp57* in genomic imprinting. **(A)** Maternal *Zfp57* is required for maintaining DNA methylation at the *Snrpn*, *AK008011*, *Cdh15*, and *Impact* ICRs, whereas there is gender effect of *Zfp57* which is stronger in the male M^−^Z^-^ embryos than female M^−^Z^-^ embryos in the maintenance of DNA methylation at the *Rasgrf1* and *AK008011* ICRs, and possibly at the *Peg13* ICR as well. **(B)** Maternal effect of *Zfp57* is manifested in the allelic expression and expression levels of a few imprinted genes. Maternal *Zfp57* is essential for the allelic expression of seven imprinted genes at the *Snrpn* imprinted region and *Zdbf2* in both female and male embryos. Maternal *Zfp57* is also required for the allelic expression of *Impact*, *Nnat* (*Peg5*), and *H13* in male embryos. The expression levels of eight imprinted genes at the *Snrpn* imprinted region are regulated by maternal *Zfp57* in both female and male embryos, whereas maternal *Zfp57* regulates the expression levels of *Impact* in the female embryos and *Zrsr1* and *Peg3os* in the male embryos, respectively. **(C)** Gender effect of *Zfp57* is observed in allelic expression switch and change of expression levels of some imprinted genes in M^−^Z^+^ or M^−^Z^-^ embryos. Allelic expression switch occurs to *Zim1* in the female M^−^Z^-^ embryos, whereas allelic expression of *Impact*, *Zdbf2, Nnat* (*Peg5*), and *H13* is compromised in the male M^−^Z^+^ embryos. Expression of *Calcr* is only significantly decreased in female M^−^Z^-^ embryos compared with female M^+^Z^+^ embryos. In contrast, the expression levels of six imprinted genes (*Slc22a3*, *Th, Ascl2*, *Mir43*, *Mir335*, and *Xlr3b*) were significantly deregulated in the male M^−^Z^-^ embryos. The expression levels of *Zrsr1* and *Peg3os* were only deregulated in the male M^−^Z^+^ embryos.

Allelic expression switch occurs to some imprinted genes in mouse embryos when *Zfp57* is lost (Jiang et al., 2021). Since DNA methylation imprint might be different at a few ICRs in the male M^−^Z^−^ embryos compared with the female ones (Figure 9A), we wonder if allelic expression of the imprinted genes could also be differentially affected in the male M^−^Z^−^ embryos compared with the female M^−^Z^−^ embryos. Unfortunately, no exonic SNP was present in *Rasgrf1* in the hybrid 129/DBA embryos, and its expression level was lower than the threshold of our RNA-seq TPM expression analysis (see *Materials and Methods*). There is no known imprinted gene located at the *AK008011* imprinted region. Therefore, we could not determine in this study if there is any gender-specific effect on allelic expression switch and expression level differences for the corresponding imprinted genes at the *Rasgrf1* and *AK008011* imprinted regions in female or male M^−^Z^−^ mutant embryos compared with the control M^+^Z^+^ embryos. This will be tested in a future study.

*Peg13* was paternally expressed in female M^+^Z^+^ and M^−^Z^+^ embryos or male M^+^Z^+^ and M^−^Z^+^ embryos (Supplementary Figure S6A-S6A', Supplementary Table S3-S4). It became partially biallelic in female M^−^Z^−^ and male M^−^Z^−^ embryos (Supplementary Figure S6A-S6A', Supplementary Table S3-S4). By contrast, *Kcnk9* at the *Peg13* imprinted region was maternally expressed and *Trappc9* at the *Peg13* imprinted region was biallelic in female M^+^Z^+^, M^−^Z^+^, and M^−^Z^−^ embryos or male M^+^Z^+^, M^−^Z^+^, and M^−^Z^−^ embryos (Supplementary Figure S6A-S6A', Supplementary Table S3-S4). Thus, partial loss of DNA methylation at *Peg13* ICR caused *Peg13* to be partially biallelic in female M^−^Z^-^ or male M^−^Z^−^ embryos. However, it had no apparent effect on allelic expression of *Kcnk9* and *Trappc9* at the *Peg13* imprinted region in female M^−^Z^−^ or male M^−^Z^−^ embryos. The residual DNA methylation at the *Peg13* ICR may be sufficient to maintain allelic expression of *Kcnk9* at the *Peg13* imprinted region in female M^−^Z^−^ or male M^−^Z^−^ embryos. Despite this, expression of *Kcnk9* was decreased in M^−^Z^−^ embryos compared with M^+^Z^+^ and M^−^Z^+^ embryos, and it was more reduced in male M^−^Z^−^ embryos than in female M^−^Z^−^ embryos, although it was not statistically significant (Supplementary Figure S6B-S6B', Supplementary Figure S6C, Table S5-S6). This is consistent with DNA methylation at the *Peg13* ICR in these embryos (Figure 1, Supplementary Figure S1).

*Usp29* at the *Peg3* imprinted region was paternally expressed in female M^+^Z^+^ and M^−^Z^+^ or male M^+^Z^+^ and M^−^Z^+^ embryos, but it was biallelic in female M^−^Z^−^ or male M^−^Z^−^ embryos (Supplementary Figure S4A, Supplementary Figure S4A', Supplementary Table S3-S4). *Zim1* at the *Peg3* imprinted region was maternally expressed in female M^+^Z^+^ and M^−^Z^+^ or male M^+^Z^+^ and M^−^Z^+^ embryos (Supplementary Figure S4A, Supplementary Figure S4A', Supplementary Table S3-S4). Although it was still maternally expressed in male M^−^Z^−^ embryos, *Zim1* was partially biallelic in female M^−^Z^−^ embryos (Table 1, Supplementary Figure S4A, Supplementary Figure S4A', Supplementary Table S3-S4). Thus, *Zim1* appeared to be affected differently in female M^−^Z^−^ or male M^−^Z^−^ embryos lacking ZFP57. Consistent with this, *Zim1* is the only imprinted gene that displayed gender-specific effect of ZFP57 with significant P-score difference (∆P-Score≥ 0.1) in the comparisons of the female and male M^−^Z^−^ embryos with their counterpart M^+^Z^+^ embryos (Figures 7C–7C’).

*Impact* was partially biallelic in male M^−^Z^+^ embryos, although it remained preferentially paternally expressed in female M^−^Z^+^ embryos (Figures 5C–5C’). *Zdbf2* was biallelic in male M^−^Z^+^ embryos and partially biallelic in female M^−^Z^+^ embryos (Supplementary Figure S4B-S4B'). *Nnat (Peg5)* became almost biallelic in male M^−^Z^+^ embryos, whereas it was preferentially paternally expressed in female M^−^Z^+^ embryos (Supplementary Figure S4C-S4C'). *H13* was slightly preferentially maternally expressed in female M^−^Z^+^ embryos, but it was biallelic in male M^−^Z^+^ embryos (Supplementary Figure S4D-S4D'). These four imprinted genes displayed gender-specific differences in allelic expression switch upon loss of maternal *Zfp57*.

We also examined allelic expression of the imprinted genes at the *Inpp5f*, *Zac1*, and *Dlk1–Dio3* imprinted regions (Supplementary Figure S7, Supplementary Table S3-S4). For the tested imprinted genes at these three imprinted regions, they all became biallelic in female or male M^−^Z^−^ embryos. Their allelic expression did not change upon loss of maternal *Zfp57* in either female or male M^−^Z^+^ embryos. Therefore, allelic expression was only affected in M^−^Z^-^ embryos for the imprinted genes at these three examined imprinted regions, and there was no difference in their allelic expression comparing male embryos with female embryos with or without ZFP57.

### Gender-specific Effect on Expression Levels of Some Imprinted Genes in the Absence of *Zfp57*

Loss of maternal *Zfp57* resulted in mis-expression of eight imprinted genes at the *Snrpn* imprinted region in both female M^−^Z^+^ embryos and male M^−^Z^+^ embryos (Figures 8B, 8B’). Two other imprinted genes (*Zrsr1* and *Peg3os*) were deregulated in the male M^−^Z^+^ embryos compared with male M^+^Z^+^ embryos, whereas the *Impact* expression level was significantly affected in the female M^−^Z^+^ embryos compared with female M^+^Z^+^ embryos (Figures 8B–8B’, 9B, Supplementary Figure S5B-S5B', Supplementary Figure S8A-S8A', Supplementary Figure S10A-10A').

We also observed some gender-specific differences in the expression levels of some imprinted genes in the female or male M^−^Z^-^ embryos compared with their counterpart female or male M^+^Z^+^ embryos (Figure 9C, Supplementary Figure S8). Expression levels of *Slc22a3*, *Ascl2*, and *Th* were significantly affected in male M^−^Z^−^ embryos compared with male M^+^Z^+^ embryos but not in female M^−^Z^−^ embryos compared with female M^+^Z^+^ embryos (Figures 8A, 8A’, Supplementary Figure S8B-S8B', Supplementary Figure S8C-S8C'). By contrast, *Calcr* was only significantly reduced in female M^−^Z^−^ embryos compared with female M^+^Z^+^ embryos (Figures 8A, 8A’, Supplementary Figure S8D-S8D').

Many imprinted genes showed similarly significant differences in their expression in both female and male M^−^Z^−^ embryos compared with the M^+^Z^+^ embryos of the same gender (Table 2, Figure 8A–8A’). Indeed, most imprinted genes at the *Dlk1–Dio3* imprinted region appeared to be similarly affected upon loss of ZFP57 comparing M^−^Z^−^ embryos with M^+^Z^+^ embryos of the same gender (Table 2, Figure 8A–8A’, Supplementary Figure S9, Supplementary Table S5-S6). *Begain*, *Dio3*, *Dio3os*, *Dlk1*, and *Rtl1* were reduced in the female M^−^Z^−^ embryos compared with female M^+^Z^+^ embryos, whereas *Rian*, *Meg3*, *Mirg*, *AF357359*, *AF357355*, *AF357425*, and *Mir410* displayed increased expression in the female M^−^Z^−^ embryos compared with female M^+^Z^+^ embryos (Table 2, Supplementary Figure S9A). These genes behaved similarly in the male M^−^Z^-^ embryos compared with male M^+^Z^+^ embryos (Table 2, Supplementary Figure S9A'). Expression of *Mir431* and *Mir335* was increased in male but not in female M^−^Z^−^ embryos compared with M^+^Z^+^ embryos of the same gender (Table 2, Figures 8A, 8A’, Supplementary Figure S9). Therefore, *Mir431* and *Mir335* were more affected in the male embryos upon loss of *Zfp57*. *Xlr3b* was significantly increased in male M^−^Z^−^ embryos compared with male M^+^Z^+^ embryos but not in the similar comparison of the female embryos (see Supplementary Figure S11B-S11B').

### No Gender Effect Was Observed on Expression Levels of Some Other Imprinted Genes Upon Loss of *Zfp57*

We also examined expression of the imprinted genes at the *Peg3*, *Inpp5f*, *Zac1*, and *Peg5* imprinted regions in the female or male embryos (Table 2, Figures 8A, 8A’, Supplementary Figure S10, Supplementary Table S5-S6). For the genes at the *Peg3* imprinted region, decreased expression of *Zim1* and increased expression of *Usp29*, *Peg3*, and *Peg3os* were similarly observed comparing female M^−^Z^−^ embryos with female M^+^Z^+^ embryos or comparing male M^−^Z^−^ embryos with male M^+^Z^+^ embryos (Table 2, Supplementary Figure S10A-S10A'). Expression of *Inpp5f* was increased in M^−^Z^−^ embryos compared with M^+^Z^+^ embryos, regardless of the gender (Table 2, Supplementary Figure S10B-S10B'). We observed increased expression of *Zac1* (*Plagl1*) and *Hymai* and decreased expression of *Phactr2* at the *Zac1* imprinted region in M^−^Z^−^ embryos compared with M^+^Z^+^ embryos, regardless of whether they are females or males (Table 2, Supplementary Figure S10C-S10C'). Increased *Nnat* (*Peg5*) expression and decreased *Blcap* expression were similarly observed at the *Peg5* imprinted region in M^−^Z^−^ embryos compared with M^+^Z^+^ embryos of the same gender (Table 2, Supplementary Figures S10D–S10D’). Decreased expression of *Ampd3* was also similarly observed in both female and male M^−^Z^−^ embryos compared with female and male M^+^Z^+^ embryos (Supplementary Figure S11A-S11A'). Therefore, no gender-specific effect was observed for the expression levels of these imprinted genes when *Zfp57* was absent in the female or male embryos.

## Discussion

ZFP57 maintained DNA methylation imprint at most known ICRs in both male and female embryos. Intriguingly, more loss of DNA methylation imprint was observed at the ICRs of three imprinted regions upon loss of ZFP57 in the male M^−^Z^−^ mutant embryos than in female M^−^Z^−^ mutant embryos (Figures 1C–1C’, 3, 9A, Supplementary Figure S1). DNA methylation was unusually high at the *Rasgrf1* ICR in one of two female M^−^Z^−^ embryos based on WGBS in our previous study (Figure 1C) (Jiang et al., 2021). This was probably due to relatively low number of mapped sequence reads of the *Rasgrf1* ICR in that female M^−^Z^−^ embryo (Jiang et al., 2021). Accordingly, statistical significance could not be achieved when DNA methylation at the *Rasgrf1* ICR was compared between two female M^−^Z^−^ embryos and two male M^−^Z^−^ embryos, despite that there was large difference in the DNA methylation level for the female M^−^Z^−^ embryos compared with male M^−^Z^−^ embryos (Supplementary Figure S1C). Nevertheless, DNA methylation was confirmed to be more severely reduced at the *Rasgrf1* and *AK008011* ICRs in male M^−^Z^−^ embryos than in the female M^−^Z^−^ embryos based on bisulfite bacterial colony sequencing (Figures 3E, 4E). It will be interesting to find out in the future if female hormones and their target genes may be involved in maintaining DNA methylation at these ICRs. It is also possible that some factors may be missing at these ICRs for the maintenance of DNA methylation in male M^−^Z^-^ mutant embryos.

Although there was no allelic expression switch for *Kcnk9* at the *Peg13* imprinted region in female M^−^Z^−^ and male M^−^Z^−^ mutant embryos compared with M^+^Z^+^ embryos based on RNA-seq analysis of another set of mouse embryos that were different from the ones used in the previous WGBS experiments, the expression of *Kcnk9* appeared to be more severely affected in male mutant embryos than in female mutant embryos (Supplementary Figure S6). There was partial biallelic expression of *Peg13* in female and male M^−^Z^−^ embryos compared with M^+^Z^+^ embryos (Supplementary Figure S6). This indicates that the residual DNA methylation at the *Peg13* imprinted region was sufficient to maintain parent-of-origin–dependent monoallelic expression of *Kcnk9*, but not *Peg13*, in female and male M^−^Z^−^ embryos, although two different sets of mouse embryos were used in RNA-seq and WGBS. Unfortunately, there is no SNP for the *Rasgrf1* transcript and no other known transcripts at the *Rasgrf1* and *AK008011* imprinted regions in the hybrid 129/DBA embryos. *Rasgrf1* transcripts in all embryo samples were below the TPM threshold (TPM>1) in our RNA-seq expression analysis. Therefore, we could not determine in this study if there was any gender effect on allelic expression switch and expression levels of the corresponding imprinted genes at these imprinted regions in M^−^Z^−^ mutant embryos compared with the control M^+^Z^+^ embryos. These will be tested in the future research.

*Zfp57* has both maternal and zygotic functions that are partially redundant in maintaining genomic imprinting at most ICRs (Jiang et al., 2021). In general, more severe loss of genomic imprinting is observed in *Zfp57* maternal–zygotic mutant (M^−^Z^−^) embryos (Jiang et al., 2021). Indeed, DNA methylation imprint was almost completely lost at most known ICRs in M^−^Z^−^ mutant embryos (Jiang et al., 2021). Interestingly, partial loss of DNA methylation imprint was observed at *Snrpn*, *Cdh15*, *Impact*, and *AK008011* ICRs in the female or male M^−^Z^+^ embryos (Figures 4D, 4D’, 5A, 5A’, 9A). Furthermore, only maternal *Zfp57* appeared to be necessary for maintaining DNA methylation at the *AK008011* and *Impact* ICRs in female embryos. This suggests that zygotic *Zfp57* is dispensable for the maintenance of DNA methylation at the *AK008011* and *Impact* ICRs in female embryos. It also implies that the maintenance mechanisms for DNA methylation at these two ICRs in female embryos may be different from those at other ICRs in mouse embryos. Maternal *Zfp57* is essential for the maintenance of DNA methylation imprint at these two ICRs in female embryos.

Maternal *Zfp57* is also required for maintaining parent-of-origin–dependent monoallelic expression of some imprinted genes (Figure 9B). Upon partial loss of DNA methylation at the *Snrpn* ICR in the female or male M^−^Z^+^ embryos, loss of maternal *Zfp57* caused partial allelic expression switch of seven imprinted genes at the *Snrpn* imprinted region as expected, although two different sets of mouse embryos were used in RNA-seq and WGBS (Figures 5B, 5B’, 9B). Complete loss of DNA methylation at the *Snrpn* ICR in the M^−^Z^−^ embryos resulted in biallelic expression of almost all imprinted genes at the *Snrpn* imprinted region, that is, complete allelic expression switch of the corresponding imprinted genes (Figure 5B, 5B’). The expression levels of these imprinted genes changed accordingly, with almost 2-fold differences for most of them in the M^−^Z^−^ embryos (Supplementary Figure S5A, S5A'). There were variable differences in the expression of these imprinted genes ranging from less than 2-fold–2-fold in the M^−^Z^+^ embryos (Figure 9B, Supplementary Figure S5A, S5A'). These are also consistent with the allelic switch models for the target imprinted genes proposed in our recent article, with partial or complete allelic switch in the M^−^Z^+^ or M^−^Z^−^ embryos, respectively (Jiang et al., 2021).

In contrast with that in the female M^−^Z^−^ embryos, partial loss of DNA methylation at the *Impact* ICR in female M^−^Z^+^ embryos did not cause allelic expression switch of the *Impact* imprinted gene according to the results obtained with two different sets of mouse embryos used in RNA-seq and WGBS (Figure 5). Its expression level was significantly increased in female M^−^Z^+^ embryos, albeit not as much as in female M^−^Z^−^ embryos, compared with female M^+^Z^+^ embryos (Supplementary Figure S5B-S5B'). Intriguingly, partial allelic expression switch occurred to *Impact* in the female M^−^Z^-^ or male M^−^Z^−^ embryos even though there was still only partial loss of DNA methylation at the *Impact* ICR, with similar loss of DNA methylation to female M^−^Z^+^ embryos (Figure 5). Furthermore, partial allelic expression switch also occurred to *Impact* in the male M^−^Z^+^ embryos without significant loss of DNA methylation at its ICR (Figure 5). We suspect that there might be some other ZFP57-dependent factors besides DNA methylation at the known *Impact* ICR that is required for maintaining parent-of-origin–dependent expression of the *Impact* imprinted gene.

Since there is no known imprinted gene at the *AK008011* imprinted region, we could not test the maternal effect of *Zfp57* on expression of any imprinted gene in response to partial loss of DNA methylation at the *AK008011* ICR in the M^−^Z^+^ embryos. Loss of maternal *Zfp57* caused allelic expression switch of *Zdbf2* in both female and male M^−^Z^+^ embryos, whereas it resulted in allelic expression switch of the three imprinted genes only in the male M^−^Z^+^ embryos (Figure 9B). *Nnat* (*Peg5*) expression level was affected in both female and male M^−^Z^+^ embryos, whereas loss of maternal *Zfp57* caused deregulation of the expression levels of *Zrsr1* and *Peg3os* in the male M^−^Z^+^ embryos and *Impact* in the female M^−^Z^+^ embryos, respectively. These will be further discussed below, together with the gender effect of ZFP57 on their expression. Taken together, the maternal effect of *Zfp57* is manifested in the maintenance of DNA methylation imprint at a few imprinted regions, in particular *Snrpn*, as well as the parent-of-origin–dependent expression of some corresponding imprinted genes in mouse embryos. It may also result in deregulation of a few other imprinted genes without concomitant loss of DNA methylation imprint.

DNA methylation imprint was similarly lost at most known imprinted regions in M^−^Z^-^ embryos compared with M^+^Z^+^ embryos of the same gender (Figure 1A, 1A’). Consequently, loss of ZFP57 caused similar allelic expression switch and expression level differences of most imprinted genes in M^−^Z^-^ embryos compared with M^+^Z^+^ embryos of the same gender that we examined, although two different sets of mouse embryos were used in RNA-seq and WGBS (Tables 1, 2). Interestingly, we observed a sexually dimorphic effect of ZFP57 on the expression of some imprinted genes as discussed below (Figure 9C).

There was not much gender-specific difference in the allelic expression of 48 imprinted genes with an exonic SNP in the wild-type female and male mouse embryos that we examined in this study, except for two X-linked *Jpx* and *Ftx* genes (Figure 7A). Expression levels were mostly similar for 105 known imprinted genes in the wild-type female and male embryos, with only *Jpx* and *Ftx* showing significant differences in their expression levels (Figure 7B).

In this relatively comprehensive study, gender effect has been observed on allelic expression as well as expression levels of a few mouse imprinted genes in response to loss of *Zfp57* or loss of just maternal *Zfp57*. *Zim1* was switched from maternal allele–specific expression to become partially biallelic only in the female M^−^Z^−^ embryos (Table 1, Figure 7C, 7C’). *Zdbf2* was partially biallelic in the female M^−^Z^+^ embryos and biallelic in male M^−^Z^+^ embryos. Allelic expression of *Impact*, *Nnat* (*Peg5*), and *H13* was also compromised in the male M^−^Z^+^ embryos, but not in the female ones (Figure 9B).

The sexually dimorphic effect of ZFP57 occurred to the expression levels of *a* few imprinted genes upon loss of *Zfp57* (Figure 9C). *Calcr* was only significantly decreased in female M^−^Z^-^ embryos compared with female M^+^Z^+^ embryos (Supplementary Figure S8). Expression levels of *Slc22a3*, *Ascl2*, and *Th* were significantly affected in male M^−^Z^−^ embryos compared with male M^+^Z^+^ embryos, but not in the similar comparisons of female embryos (Supplementary Figure S8). *Mir431* and *Mir335* at the *Dlk1–Dio3* imprinted region were also more severely affected in male M^−^Z^−^ embryos (Table 2, Figures 8A, 8A’, Supplementary Figure S9). *Xlr3b* was only significantly increased in male M^−^Z^−^ embryos compared with male M^+^Z^+^ embryos (Supplementary Figure S11B-S11B'). Furthermore, the expression levels of *Zrsr1* and *Peg3os* were only deregulated in the male (but not female) M^−^Z^+^ embryos compared with male M^+^Z^+^ embryos. These results suggest that ZFP57 exerts a sexually dimorphic effect in the expression of some imprinted genes. It also means that we may need to analyze gender effect on some phenotypes caused by the loss of *Zfp57* in the future.

In our current study, no imprinted genes exhibit gender-specific difference in their expression levels in the wild-type female and male embryos, except for two X-linked genes as expected. *Zfp57* displays maternal–zygotic effect in maintaining genomic imprinting at most imprinted regions in mouse embryos. It also exerts maternal and sexual dimorphic effects on DNA methylation at a subset of imprinted regions. These effects of *Zfp57* are manifested in allelic expression switch and expression level changes of a number of known imprinted genes.

## Materials and Methods

### Mouse Timed Pregnancy Mating for 129/DBA Hybrid Embryos

The mice carrying the *Zfp57* deleted mutant allele on the 129S6/SvEvTac genetic background were generated in the original study, and they were called the 129 mice in this study (Li et al., 2008). We also performed 12 backcrosses for the 129 mice carrying the *Zfp57* deleted mutant allele with the wild-type DBA/2J mice to obtain *Zfp57* heterozygous mice mainly on the DBA/2J background that was named *Zfp57* ^*+/−*^ (DBA*) in a recent study (Jiang et al., 2021). Then, timed pregnancy mating was set up between *Zfp57* ^*−/−*^ homozygous 129 female mice and *Zfp57* ^*+/−*^ (DBA*) male mice to obtain 129/DBA hybrid E13.5 embryos. The mouse vaginal plug was checked daily. The female mice were presumed to be potentially pregnant for 0.5 days when their vaginal plug was found around the noon of the day. The pregnant female mice were dissected for 129/DBA hybrid E13.5 embryos used for RNA-seq analysis as well as whole-genome bisulfite sequencing (WGBS) analysis.

### Polymerase Chain Reaction (PCR) Genotyping for Determining the Gender of the Female and Male Embryos

To determine the gender of these 129/DBA hybrid E13.5 embryos, SRY genotyping was carried out with the primers SRY-F1 and SRY-R1 for a PCR product of 266 bp present in the male embryos but absent in the female embryos. The sequence for SRY-F1 is 5’- CCA​CTC​CTC​TGT​GAC​ACT, whereas the sequence for SRY-R1 is 5’- GAG​AGC​ATG​GAG​GGC​CAT.

### Total RNA Samples Purified From the Female and Male Embryos for RNA-seq Analysis

Total RNA samples were obtained from the 129/DBA hybrid E13.5 embryos from the timed pregnancy mating. At least three female embryos of each genotype of M^+^Z^+^, M^−^Z^+^, and M^−^Z^-^ were used for RNA purification and sent out for RNA-seq analysis. Similarly, total RNA samples were purified from at least three male embryos of each genotype of M^+^Z^+^, M^−^Z^+^, and M^−^Z^-^ and subjected to RNA-seq analysis. The data analyses were performed as described below.

### RNA-Seq Analysis of the Expression Levels of the Imprinted Genes in the Female and Male Embryos

RNA-seq analysis was performed for the RNA samples obtained from the mouse embryos that were different from the ones used in WGBS of the previous study. At least three embryos were analyzed for each genotype of female M^+^Z^+^, M^−^Z^+^, and M^−^Z^−^ 129/DBA hybrid E13.5 embryos and male M^+^Z^+^, M^−^Z^+^, and M^−^Z^−^ 129/DBA hybrid E13.5 embryos.

First, the quality of the sequence reads obtained from RNA-seq was assessed by FastQC (v0.11.9) downloaded from the website (www.bioinformatics.babraham.ac.uk/projects/fastqc/). Then, the adapter sequences were trimmed from the sequence reads by using Trim_Galore (v0.6.6) (https://github.com/FelixKrueger/TrimGalore). Only good quality sequence reads were aligned to the mouse reference genome (mm9) on the UCSC website using STAR (v2.7.8a), with the setting of “–outFilterMultimapNmax 1 –alignEndsType EndToEnd–outSAMattributes NH HI NM MD” plus other default parameters (Dobin et al., 2013). The sequence reads were mapped to the annotated genes (UCSC mm9. gtf) with the featureCounts function (-O–s 2 option) of Subread (v.2.0.1) (Liao et al., 2014). Out of 148 mouse imprinted genes listed on the website of the geneimprint database: www.geneimprint.com, 139 had been mapped to the mouse reference genome (mm9) and then used for expression analyses in this study.

Transcripts per million (TPM) values were calculated for 139 mapped imprinted genes with more than 10 sequence reads. 105 imprinted genes with the TPM value of more than 1.0 in at least one embryo sample were selected for further analysis of their expression differences across different genotypes or genders. The log_2_FC values were used to quantify the fold change (FC) of gene expression based on the ratios of TPM for this imprinted gene in three genotypes of M^+^Z^+^, M^−^Z^+^, and M^−^Z^−^. The differentially expressed genes in the RNA-seq data of different genotypes were first identified by DESeq2 before they were used for constructing the intensity difference heatmaps (Love et al., 2014). The cutoff threshold was set for |log_2_FC |> 0.3 (*p* < 0.05) for the intensity difference heatmap when the expression levels of the imprinted genes were compared between six female M^+^Z^+^ embryos and four male M^+^Z^+^ embryos (Figure 7B). It was set for |log_2_FC |> 0.5 (*p* < 0.05) in other intensity difference heatmaps comparing their expression levels in the M^+^Z^+^, M^−^Z^+^, and M^−^Z^−^ embryos of the same gender (Figure 8).

### Allelic Expression Analyses of the Imprinted Genes in the Female and Male Embryos by RNA-Seq

The SNPs present in the 129/DBA hybrid embryos were determined by using SNP calling with the Genome Analysis Toolkit (GATK) software package (McKenna et al., 2010; Van der Auwera et al., 2013). These SNPs were used for creating N-masked genomes from the reference genome (mm9) using BEDTools with incorporation of “N” at the position of an SNP (Quinlan and Hall, 2010). Mapping of the sequence reads to the N-masked genomes was performed using STAR (v2.5.4a), with the setting of “–outFilterMultimapNmax 1 –alignEndsType EndToEnd–outSAMattributes NH HI NM MD” and other default parameters (Dobin et al., 2013). The mapped sequence reads were assigned to the maternal (129) or paternal (DBA) genome using SNPsplit (v0.4.0), and then a file was generated with the SNPs (Krueger and Andrews, 2016). The allele-specific sequence reads were assigned to the maternal and paternal alleles of the imprinted genes using featureCounts (Liao et al., 2014). Further analyses in this study were limited to the genes with more than 10 counts based on the sum of the mapped reads of maternal and paternal alleles.

P-score was calculated for assessing the proportion of the transcripts derived from the paternal allele of each imprinted gene based on the following equation: P-score = P/(M + P)-0.5. P stands for the number of the paternal (P) allele transcript reads of an imprinted gene, whereas M stands for the number of the maternal (M) allele transcript reads of an imprinted gene. This was adapted from the calculation method for allelic expression of X-linked genes in a recent study (Yu et al., 2021). The imprinted genes with statistically significant allelic expression difference (∆P-Score≥ 0.1, *p* < 0.05) used for intensity difference heatmap analyses were identified by comparing their P-score values among different genotypes by using the Kruskal–Wallis test (Figure 7). Similar results were obtained by using the binomial test used in a recent study (Yu et al., 2021).

### Whole-Genome Bisulfite Sequencing Analysis of 129/DBA Hybrid Female and Male Embryos

The WGBS data were based on the previous study (Jiang et al., 2021). The gender of these female or male embryos used for WGBS was determined by PCR genotyping of the *Sry* gene as described above. Please refer to the previous article for the WGBS sequence depth and genome coverage (Jiang et al., 2021). The WGBS sequence reads of these embryo samples were mapped to the abovementioned N-masked reference genome. The CpG sites present in at least three unique reads were used for quantification of DNA methylation of an ICR in the subsequent analyses. The methylated and unmethylated C residues were identified at each CpG site of an ICR. Then, the DNA methylation level was quantified for each CpG site of this ICR based on the number of methylated C residues divided by the total number of C residues for this CpG site. The percentage (%) of DNA methylation for the ICR was the average of DNA methylation levels of all CpG sites at this ICR.

SNPsplit (v0.4.0) was used to separate the allelic DNA methylation reads of the ICRs with the default parameters (Krueger and Andrews, 2016). Only CpG sites covered by at least one unique sequence read were subject to measurement of DNA methylation levels of the maternal or paternal ICR. Allelic DNA methylation was determined for only a few ICRs with SNPs.

### Bisulfite Bacterial Colony Sequencing

Genomic DNA samples purified from the mouse embryos were subjected to bisulfite mutagenesis with the EZ DNA Methylation-Gold™ Kit (Zymo Research). Then, bisulfite PCR was carried out for a *Rasgrf1* ICR region with two rounds of nested PCR reactions (Zuo et al., 2012; Takikawa et al., 2013a). The primers used for the first round of bisulfite PCR were Ras-Bis-OF with the sequence of 5’- GTT​ATT​ATT​ATG​TGT​TAT​GTG​TAG​TAA​G and Ras-Bis-OR1 with the sequence of 5’- TAA​TAC​AAC​AAC​AAC​AAT​AAC​AAT​C. The primers used for the second round of nested bisulfite PCR were Ras-Bis-IF with the sequence of 5’- GGT​GTA​GAA​TAT​GGG​GTT​G and Ras-Bis-IR with the sequence of 5’- ATA​CAA​CAA​CAA​CAA​TAA​C. Two specific PCR products of 321 bp and 408 bp were obtained after the second round of nested bisulfite PCR, and they had completely overlapping 321-bp long sequences at one end. Both specific PCR products were cloned into the pUCm-T vector (Sangon, cat# B522213), and the resultant bacterial colonies were sequenced to determine the methylation status of the CpG sites within the 321-bp region of the *Rasgrf1* ICR. The obtained sequences were analyzed with the web-based program called QUMA (http://quma.cdb.riken.jp/). Then, DNA methylation status was determined for the CpG sites in the *Rasgrf1* ICR as shown in Figure 3.

Bacterial colony bisulfite sequencing was similarly carried out for the *AK008011* ICR with the genomic DNA samples derived from 3–4 M^+^Z^+^, M^−^Z^+^, or M^−^Z^−^ E13.5 embryos of each gender that had also been used for RNA-seq analyses in this study (Figures 4, 7). Two rounds of nested PCR reactions were performed for the bisulfite genomic DNA product. The primers used for the first round of bisulfite PCR were AK-Bis-OFn1 with the sequence of 5’- GGT​TTA​GTT​AGG​GAA​AGG​GT and AK-Bis-ORn1 with the sequence of 5’- CAC​ACA​CCT​AAA​TCC​TAA​CAC​T, with the PCR product of 765 bp. The primers used for the second round of nested bisulfite PCR were AK-Bis-OFn2 with the sequence of 5’- GTG​GTT​ATA​TAT​TGT​AGG​GTA​GG and AK-Bis-ORn2 with the sequence of 5’- CCT​ACA​TAA​TTA​AAA​CCT​ACC​TC. The obtained PCR product of 605 bp was cloned into the pUCm-T vector (Sangon, cat# B522213), and the resultant bacterial colonies were sequenced to determine the methylation status of the CpG sites of the *AK008011* ICR. The obtained sequences were analyzed with the web-based program called QUMA (http://quma.cdb.riken.jp/). Then, DNA methylation status was determined for the CpG sites in the *AK008011* ICR as shown in Figure 4.

### Statistical Analysis

Except for statistical analyses used to generate the intensity difference heatmaps, ANOVA (Fisher LSD) was used for statistical analysis of imprinted gene expression (FC value and P-score) differences among the RNA-seq samples of different genotypes of the same gender. And, two-tailed Student’s t-test was used for analyzing the statistical significance when expression of the imprinted genes was compared between the female samples and the male samples within the same genotype.

ANOVA (Fisher LSD) was similarly utilized in analyzing the statistical significance of DNA methylation at the ICRs of different genotypes of the same gender based on the WGBS data. And, two-tailed Student’s t-test was used to compare DNA methylation at the ICRs of the same genotype between the female samples and male samples. Calculation of the *p*-value for all statistical analyses was carried out by using the open source R software (https://www.R-project.org).

## Data Availability

The datasets presented in this study can be found in online repositories. The names of the repository/repositories and accession number(s) can be found below: https://www.ncbi.nlm.nih.gov/geo/, GSE165079; https://www.ncbi.nlm.nih.gov/geo/, GSE189761.
