# Supplementary material for: Zfp57 Exerts Maternal and Sexually Dimorphic Effects on Genomic Imprinting
Source: Front Cell Dev Biol. 2022 Feb 2;10:784128. doi: 10.3389/fcell.2022.784128 (PMC8895500; doi:10.3389/fcell.2022.784128)
Supplement: Supplementary file 1 [file DataSheet1.pdf]

Zfp57 exerts maternal and sexually dimorphic effects on genomic imprinting

Zhen Xu[1,*], Jiajia Shi[1,*], Yu Zhang[2,*], Yuhan Liu[1], Junzheng Zhao[1], Qian Chen[1], Chenglin Song[1], Shuhui Geng[1], Wei Xie[2], Feizhen Wu[3], Yun Bai[1], Yang Yang[1], Xiajun Li[1,$]

[1]School of Life Science and Technology, ShanghaiTech University, Shanghai 201210, China

[2]Center for Stem Cell Biology and Regenerative Medicine, MOE Key Laboratory of Bioinformatics, Tsinghua-Peking Center for Life Sciences, School of Life Sciences, Tsinghua University, Beijing 100084, China

[3]Institutes of Biomedical Sciences, Shanghai Medical College of Fudan University, Shanghai 200032, China

Supplemental Figure Legends

Figure S1. Statistical analysis was performed for DNA methylation at the imprinted regions comparing male embryos with female ones. Genomic DNA samples were isolated from the *Zfp57*$^{+/-}$ (M^+Z^+), *Zfp57*$^{-/+}$ (M^-Z^+) and *Zfp57*$^{-/-}$ (M^-Z^-) hybrid E13.5 embryos from the timed mating between *Zfp57*$^{+/-}$ (or *Zfp57*$^{-/-}$) 129 female mice and *Zfp57*$^{+/-}$ (DBA*) male mice mainly on the DBA/2J genetic background as reported in the previous study (12). Two female embryos of each genotype and two male embryos of each genotype were used for whole-genome bisulfite sequencing (WGBS) analysis. Two-tailed Student's t-test was used to compare DNA methylation at all known 24 ICRs of the same genotype (M^+Z^+, M^-Z^+, M^-Z^-) between the female (light grey) samples and male (dark grey) samples (A-C). One-way ANOVA (Fisher LSD) was used for statistical analysis of DNA methylation at each ICR of different genotypes in the same gender (D-D', E-E'). The DNA methylation data were plotted as mean ± SEM in the bar graphs. Statistical significance: *, $p< 0.05$. **, $p< 0.01$.

(A) DNA methylation was compared at all known 24 ICRs between female and male M^+Z^+ embryos.

(B) DNA methylation was compared at all known 24 ICRs between female and male M^-Z^+ embryos.

(C) DNA methylation was compared at all known 24 ICRs between female and male M^-Z^- embryos.

(D) Statistical analysis was carried out for DNA methylation at the *Peg13, Rasgrf1* and *AK008011* ICRs in the female M^+Z^+, M^-Z^+ and M^-Z^- embryos. DNA methylation was significantly reduced at the *AK008011* ICR in the female M^-Z^+ and M^-Z^- embryos compared with the female M^+Z^+ embryos. DNA methylation was close to significantly reduced ($p= 0.05$) at the *Peg13* ICR in the female M^-Z^- embryos compared with the female M^+Z^+ embryos.

(D') Statistical analysis was carried out for DNA methylation at the *Peg13, Rasgrf1* and *AK008011* ICRs in the male M^+Z^+, M^-Z^+ and M^-Z^- embryos. DNA methylation was

significantly reduced at the *Peg13* and *Rasgrf1* ICRs in the male M^-Z^- embryos compared with the male M^+Z^+ and M^-Z^+ embryos. DNA methylation was close to significantly reduced at the *AK008011* ICR in the male M^-Z^+ (p= 0.09) and M^-Z^- embryos (p= 0.06) compared with the male M^+Z^+ embryos.

(*E*) Statistical analysis results were shown for DNA methylation at the *Snrpn*, *Impact* and *Cdh15* ICRs in the female M^+Z^+, M^-Z^+ and M^-Z^- embryos. DNA methylation was significantly reduced at these three ICRs in the female M^-Z^+ and M^-Z^- embryos compared with the female M^+Z^+ embryos. It was significantly reduced at the *Cdh15* ICR and close to significantly reduced (p= 0.05) at the *Snrpn* ICR in the female M^-Z^- embryos compared with the female M^-Z^+ embryos.

(*E'*) Statistical analysis results were shown for DNA methylation at the *Snrpn*, *Impact* and *Cdh15* ICRs in the male M^+Z^+, M^-Z^+ and M^-Z^- embryos. DNA methylation was significantly reduced at these three ICRs in the male M^-Z^- embryos compared with the male M^+Z^+ embryos. It was significantly reduced at the *Snrpn* and *Impact* ICRs in the male M^-Z^- embryos compared with the male M^-Z^+ embryos. It was also close to significantly reduced (p= 0.05) at the *Snrpn* ICR in the male M^-Z^+ embryos compared with the male M^+Z^+ embryos.

Figure S2. DNA methylation imprint was similarly lost in female and male *Zfp57* maternal-zygotic mutant embryos at most imprinted regions as exemplified by three ICRs here. The methylation IGV plot, with the same scale (0-1), is shown for the *Inpp5f, Zac1* and IG-DMR ICRs in two female embryos (E1, E2) or two male embryos (E1, E2) for each of the M^+Z^+, M^-Z^+ and M^-Z^- genotypes.

(*A*) The methylation IGV plot of *Inpp5f* ICR (mm9, chr7:135831788-135832156) in the female embryos.

(*A'*) The methylation IGV plot of *Inpp5f* ICR (mm9, chr7:135831788-135832156) in the male embryos.

(*B*) The methylation IGV plot of *Zac1* ICR (mm9, chr10:12810119-12811967) in the female embryos.

(*B'*) The methylation IGV plot of *Zac1* ICR (mm9, chr10:12810119-12811967) in the male embryos.

(*C*) The methylation IGV plot of the IG-DMR (mm9, chr12:110764950-110766944) of the *Dlk1-Dio3* imprinted region in the female embryos.

(*C'*) The methylation IGV plot of the IG-DMR (mm9, chr12:110764950-110766944) of the *Dlk1-Dio3* imprinted region in the male embryos.

Figure S3. Maternal effect of *Zfp57* was observed on maintenance of ICR methylation at the *Snrpn* and *Impact* imprinted regions on the allelic methylation IGV plot. After WGBS analysis of the *Zfp57*$^{+/+}$ (M^+Z^+), *Zfp57*$^{-/+}$ (M^-Z^+) and *Zfp57*$^{-/-}$ (M^-Z^-) hybrid 129/DBA embryos, the allelic methylation IGV plots are generated for the *Snrpn* and *Impact* ICRs on the paternal (P) or maternal (M) chromosome in two female embryos (E1, E2) or two male embryos (E1, E2) of each genotype. DNA methylation imprint on the maternal chromosome for these two ICRs was partially lost in the M^-Z^+ embryos, although more severe loss of ICR methylation was observed in the M^-Z^- embryos. The scale (0-1) is the same for all IGV plots in the figure.

(A, A') The allelic methylation IGV plot of the *Snrpn* ICR (mm9, chr7:67148026-67150181) in the female (A) or male (A') embryos.

(*B, B'*) The allelic methylation IGV plot of the *Impact* ICR (mm9, chr18:13131377-13131664) in the female (B) or male (B') embryos.

Figure S4. Parent-of-origin-dependent expression of a few imprinted genes was differentially affected in the female or male embryos lacking maternal *Zfp57*. Total RNA samples were isolated from 129/DBA hybrid female M^+Z^+, M^-Z^+ and M^-Z^- or male M^+Z^+, M^-Z^+ and M^-Z^- E13.5 embryos derived from the timed mating between *Zfp57*$^{+/-}$ (or *Zfp57*$^{-/-}$) 129 female mice and *Zfp57*$^{+/-}$ DBA* male mice, similar to the previous study (12). Then RNA-seq analysis was performed for at least three female embryos of each genotype and three male embryos of each genotype (M^+Z^+, M^-Z^+ and M^-Z^-). The SNPs present in the exons of

the imprinted genes were employed to determine the allelic expression of the corresponding imprinted genes. P-score was used to calculate expression of the paternal alleles of the imprinted genes (Table S3-S4), which is described in the Materials and Methods section. Then the data plotted as mean ± SEM were analyzed by two-way ANOVA (A-A') or one-way ANOVA (B-B', C-C', D-D') to test the statistical significance: *, $p< 0.05$. **, $p< 0.01$. ***, $p< 0.001$.

(*A, A'*) Expression of the paternal alleles of *Zim1* and *Usp29* at the *Peg3* imprinted region in the female (A) or male (A') embryos.

(*B, B'*) Expression of the paternal allele of the *Zdbf2* imprinted gene at the *Gpr1-Zdbf2* imprinted region in the female (B) or male (B') embryos.

(*C, C'*) Expression of the paternal allele of *Nnat (Peg5)* at the *Peg5/Nnat* imprinted region in the female (C) or male (C') embryos.

(*D, D'*) Expression of the paternal allele of the *H13* imprinted gene at the *Mcts2* imprinted region in the female (D) or male (D') embryos.

Figure S5. Maternal effect of *Zfp57* was observed on expression of the imprinted genes at the *Snrpn* imprinted region. RNA-seq analysis was carried out for at least three female embryos and three male embryos of each genotype (M^+Z^+, M^-Z^+ and M^-Z^-). The fold change (FC) in the Log_2 scale (Log_2FC) was used to measure relative expression levels of the imprinted genes in the M^+Z^+, M^-Z^+ and M^-Z^- embryos based on the TPM values (Table S5-S6). Then the expression level differences were analyzed by two-way (A-A') or one-way (B-B', C-C') ANOVA to test the statistical significance. The Log_2FC data were plotted as mean ± SEM in the bar graphs. Statistical significance: *, $p< 0.05$. **, $p< 0.01$. ***, $p< 0.001$.

(*A, A'*) Expression of the imprinted genes at the *Snrpn* imprinted region in the female embryos (A) or male embryos (A'). These include the *Atp10a*, *Ube3a*, *Snord64*, *Snrpn*, *Snurf*, *Ndn*, *Ipw*, *Magel2*, *Mkrn3* and *Peg12* imprinted genes at the *Snrpn* imprinted region.

(*B, B'*) Expression of *Impact* in the female embryos (B) or male embryos (B').

(*C, C'*) Expression of *Cdh15* in the female embryos (C) or male embryos (C').

Figure S6. Expression of the imprinted genes at the *Peg13* imprinted region. Total RNA samples were isolated from M^+Z^+, M^-Z^+ and M^-Z^- hybrid E13.5 embryos derived from the timed mating between *Zfp57*$^{+/-}$ (or *Zfp57*$^{-/-}$) 129 female mice and *Zfp57*$^{+/-}$ DBA* male mice. RNA-seq analysis was performed for at least three female embryos or three male embryos of each genotype (M^+Z^+, M^-Z^+ and M^-Z^-). The SNPs present in the exons of the imprinted genes were employed to determine the allelic expression of the corresponding imprinted genes. P-score described in Materials and Methods was used to calculate expression of the paternal alleles of the *Kcnk9*, *Peg13* and *Trappc9* imprinted genes at the *Peg13* imprinted region in the M^+Z^+, M^-Z^+ and M^-Z^- embryos of the same gender (Table S3-S4), whereas Log_2FC was used to measure relative expression levels of these imprinted genes in the M^+Z^+, M^-Z^+ and M^-Z^- embryos of the same gender based on the TPM values (Table S5-S6). Then the data were analyzed by two-way ANOVA (A-A', B-B') or two-tailed Student's t-test (C) to test the statistical significance. The P-score and Log_2FC data were plotted as mean ± SEM in the bar graphs. Statistical significance: *, $p< 0.05$. **, $p< 0.01$. ***, $p< 0.001$.

(*A, A'*) Expression of the paternal alleles of *Kcnk9*, *Peg13* and *Trappc9* in the female (A) or male (A') embryos.

(*B, B'*) Expression of *Kcnk9*, *Peg13* and *Trappc9* in the female (B) or male (B') embryos.

(*C*) Two-tailed Student's t-test was carried out to compare expression of *Kcnk9* in the female M^+Z^+, M^-Z^+ and M^-Z^- embryos with its expression in the male M^+Z^+, M^-Z^+ and M^-Z^- embryos of the same genotype. f, female embryos. m, male embryos.

Figure S7. Parent-of-origin-dependent expression was similarly lost for the imprinted genes at three imprinted regions in female or male *Zfp57* maternal-zygotic mutant embryos. RNA-seq analysis was performed for female and male M^+Z^+, M^-Z^+ and M^-Z^- hybrid E13.5 embryos derived from the timed mating between *Zfp57*$^{+/-}$ (or *Zfp57*$^{-/-}$) 129 female mice and *Zfp57*$^{+/-}$ DBA* male mice. At least three female embryos and three male embryos of

each genotype (M^+Z^+, M^-Z^+ and M^-Z^-) were used for RNA-seq analysis to examine allelic expression of the imprinted genes at the *Inpp5f*, *Zac1* and *Dlk1-Dio3* imprinted regions. P-score was used to calculate expression of the paternal alleles of the imprinted genes (Table S3-S4). Then the data plotted as mean ± SEM were analyzed by one-way ANOVA (A-A') or two-way ANOVA (B-B', C-C') to test the statistical significance: *, $p < 0.05$. **, $p < 0.01$. ***, $p < 0.001$.

(*A, A'*) Expression of the paternal allele of *Inpp5f* at the *Inpp5f* imprinted region in the female (A) or male (A') embryos.

(*B, B'*) Expression of the paternal alleles of *Zac1 (Plagl1), Hymai* and *Phactr2* at the *Zac1* imprinted region in the female (B) or male (B') embryos.

(*C, C'*) Expression of the paternal alleles of *Begain, Dio3os, Dlk1, Rian,* and *Meg3* at the *Dlk1-Dio3* imprinted region in the female (C) or male (C') embryos.

Figure S8. Sexually dimorphic effect was observed on expression of some imprinted genes in the absence of *Zfp57* or without just maternal *Zfp57* in mouse embryos. Total RNA samples were isolated from M^+Z^+, M^-Z^+ and M^-Z^- hybrid E13.5 embryos derived from the timed mating between $Zfp57^{+/-}$ (or $Zfp57^{-/-}$) 129 female mice and $Zfp57^{+/-}$ DBA* male mice. RNA-seq analysis was performed for at least three female embryos or three male embryos of each genotype (M^+Z^+, M^-Z^+ and M^-Z^-). Log_2FC described in Materials and Methods was used to measure relative expression levels of the imprinted genes in the M^+Z^+, M^-Z^+ and M^-Z^- embryos of the same gender (A-A', B-B') or between the female and male embryos of the same genotype (C-D) based on the TPM values (Table S5-S6). Then the Log_2FC data plotted as mean ± SEM in the bar graphs were analyzed by two-way ANOVA (A-A', B-B', C-C') or one-way ANOVA (D-D') to test the statistical significance: *, $p < 0.05$. **, $p < 0.01$. ***, $p < 0.001$.

(*A, A'*) Expression of *Zrsr1* and *Commd1* at the *Zrsr1* imprinted region in the female (A) or male (A') embryos.

(*B, B'*) Expression of *Igf2r* and *Slc22a3* at the *Igf2r* imprinted region in the female (B) or male (B') embryos.

(*C, C'*) Expression of *Ascl2* and *Th* at the *Kcnq1ot1* imprinted region in the female (C) or male (C') embryos.

(*D, D'*) Expression of *Calcr* at the *Peg10* imprinted region in the female (D) or male (D') embryos.

Figure S9. Expression of the imprinted genes at the *Dlk1-Dio3* imprinted region. Total RNA samples were isolated from M^+Z^+, M^-Z^+ and M^-Z^- hybrid E13.5 embryos derived from the timed mating between $Zfp57^{+/-}$ (or $Zfp57^{-/-}$) 129 female mice and $Zfp57^{+/-}$ DBA* male mice. RNA-seq analysis was performed for at least three female embryos or three male embryos of each genotype (M^+Z^+, M^-Z^+ and M^-Z^-). Log_2FC described in Materials and Methods was used to measure relative expression levels of the imprinted genes at the *Dlk1-Dio3* imprinted region in the M^+Z^+, M^-Z^+ and M^-Z^- embryos of the same gender based on the TPM values (Table S5-S6). Then the data plotted as mean ± SEM in the bar graphs were analyzed by two-way ANOVA to test the statistical significance: *, $p< 0.05$. **, $p< 0.01$. ***, $p< 0.001$.

(*A, A'*) Expression of *Begain, Dio3, Dio3os, Dlk1, Rian, Rtl1, AF357359, Meg3, Mirg, AF357355, AF357425, Mir410, Mir431* and *Mir335* at the *Dlk1-Dio3* imprinted region in the female (A) or male (A') embryos.

Figure S10. Expression of most imprinted genes was similarly affected in female or male *Zfp57* maternal-zygotic mutant embryos. Total RNA samples were isolated from M^+Z^+, M^-Z^+ and M^-Z^- hybrid E13.5 embryos derived from the timed mating between $Zfp57^{+/-}$ (or $Zfp57^{-/-}$) 129 female mice and $Zfp57^{+/-}$ DBA* male mice. RNA-seq analysis was performed for at least three female embryos or three male embryos of each genotype (M^+Z^+, M^-Z^+ and M^-Z^-). Log_2FC described in Materials and Methods was used to measure relative expression levels of the imprinted genes in the M^+Z^+, M^-Z^+ and M^-Z^- embryos of the same gender based on the TPM values (Table S5-S6). Then the data plotted as mean ± SEM in the bar graphs

were analyzed by two-way ANOVA (A-A’, C-C’, D-D’) or one-way ANOVA (B-B’) to test the statistical significance: *, p< 0.05. **, p< 0.01. ***, p< 0.001.

(*A, A’*) Expression of *Zim1, Usp29, Peg3* and *Peg3os* at the *Peg3* imprinted region in the female (A) or male (A’) embryos.

(*B, B’*) Expression of *Inpp5f* at the *Inpp5f* imprinted region in the female (B) or male (B’) embryos.

(*C, C’*) Expression of *Zac1 (Plagl1), Hymai* and *Phactr2* at the *Zac1* imprinted region in the female (C) or male (C’) embryos.

(*D, D’*) Expression of *Nnat (Peg5)* and *Blcap* at the *Peg5* imprinted region in the female (D) or male (D’) embryos.

Figure S11. Expression of the *Xlr3b* imprinted gene was increased in the male *Zfp57* maternal-zygotic mutant embryos but not in the female mutant embryos. Total RNA samples were isolated from M^+Z^+, M^-Z^+ and M^-Z^- hybrid E13.5 embryos derived from the timed mating between *Zfp57*$^{+/-}$ (or *Zfp57*$^{-/-}$) 129 female mice and *Zfp57*$^{+/-}$ DBA* male mice. RNA-seq analysis was performed for at least three female embryos or three male embryos of each genotype (M^+Z^+, M^-Z^+ and M^-Z^- indicated by red, blue and grey bars, respectively). Log_2FC described in Materials and Methods was used to measure relative expression levels of the imprinted genes in the M^+Z^+, M^-Z^+ and M^-Z^- embryos of the same gender based on the TPM values (Table S5-S6). Then the data plotted as mean ± SEM in the bar graphs were analyzed by one-way ANOVA (A-A’, B-B’) to test the statistical significance: *, p< 0.05. **, p< 0.01. ***, p< 0.001.

(*A, A’*) Expression of *Ampd3* in the female (A) or male (A’) embryos.

(*B, B’*) Expression of *Xlr3b* in the female (B) or male (B’) embryos.

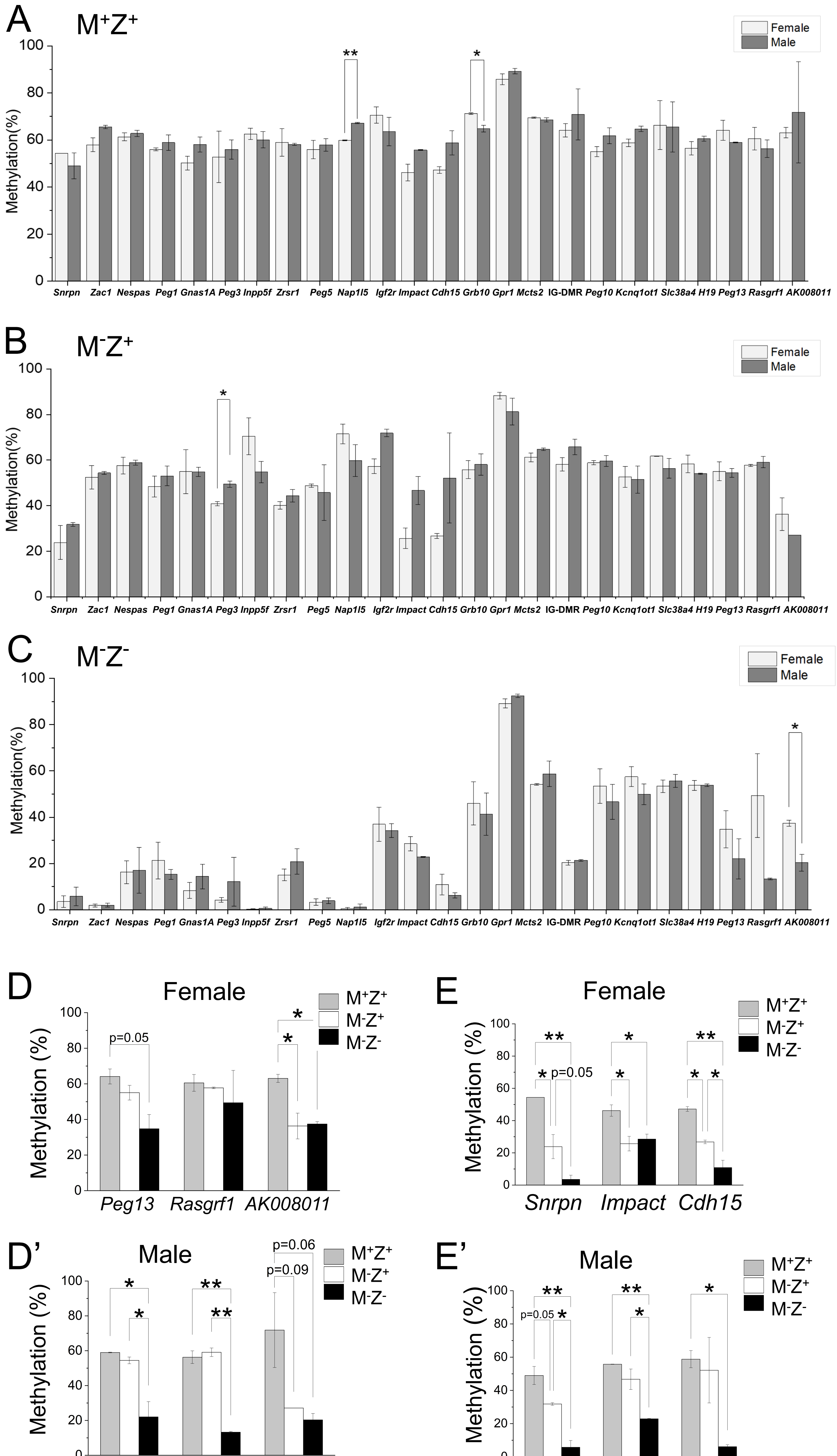
A
M+Z+
Female
Male
Methylation(%)
Snrpn Zac1 Nespas Peg1 Gnas1A Peg3 Inpp5f Zrsr1 Peg5 Nap1l5 Igf2r Impact Cdh15 Grb10 Gpr1 Mcts2 IG-DMR Peg10 Kcnq1ot1 Slc38a4 H19 Peg13 Rasgrf1 AK008011
B
M-Z+
C
M-Z-
D
Female
p=0.05
Peg13 Rasgrf1 AK008011
D'
Male
p=0.06
p=0.09
E
Snrpn Impact Cdh15
E'
p=0.05

Fig. S1

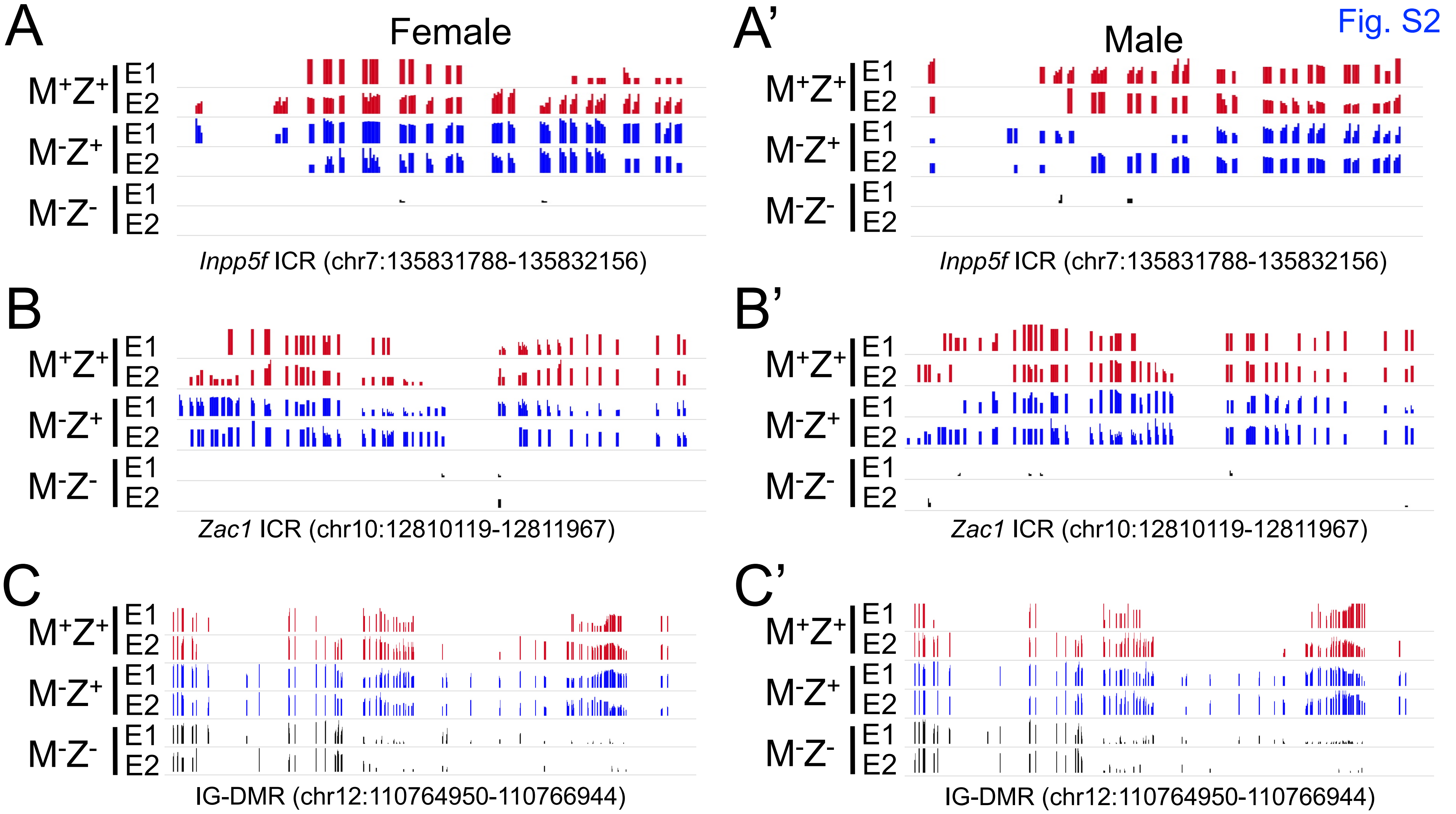

Fig. S2
A
Female
M+Z+
E1
E2
M-Z+
E1
E2
M-Z-
E1
E2
Inpp5f ICR (chr7:135831788-135832156)
A'
Male
M+Z+
E1
E2
M-Z+
E1
E2
M-Z-
E1
E2
Inpp5f ICR (chr7:135831788-135832156)
B
Zac1 ICR (chr10:12810119-12811967)
B'
Zac1 ICR (chr10:12810119-12811967)
C
IG-DMR (chr12:110764950-110766944)
C'
IG-DMR (chr12:110764950-110766944)

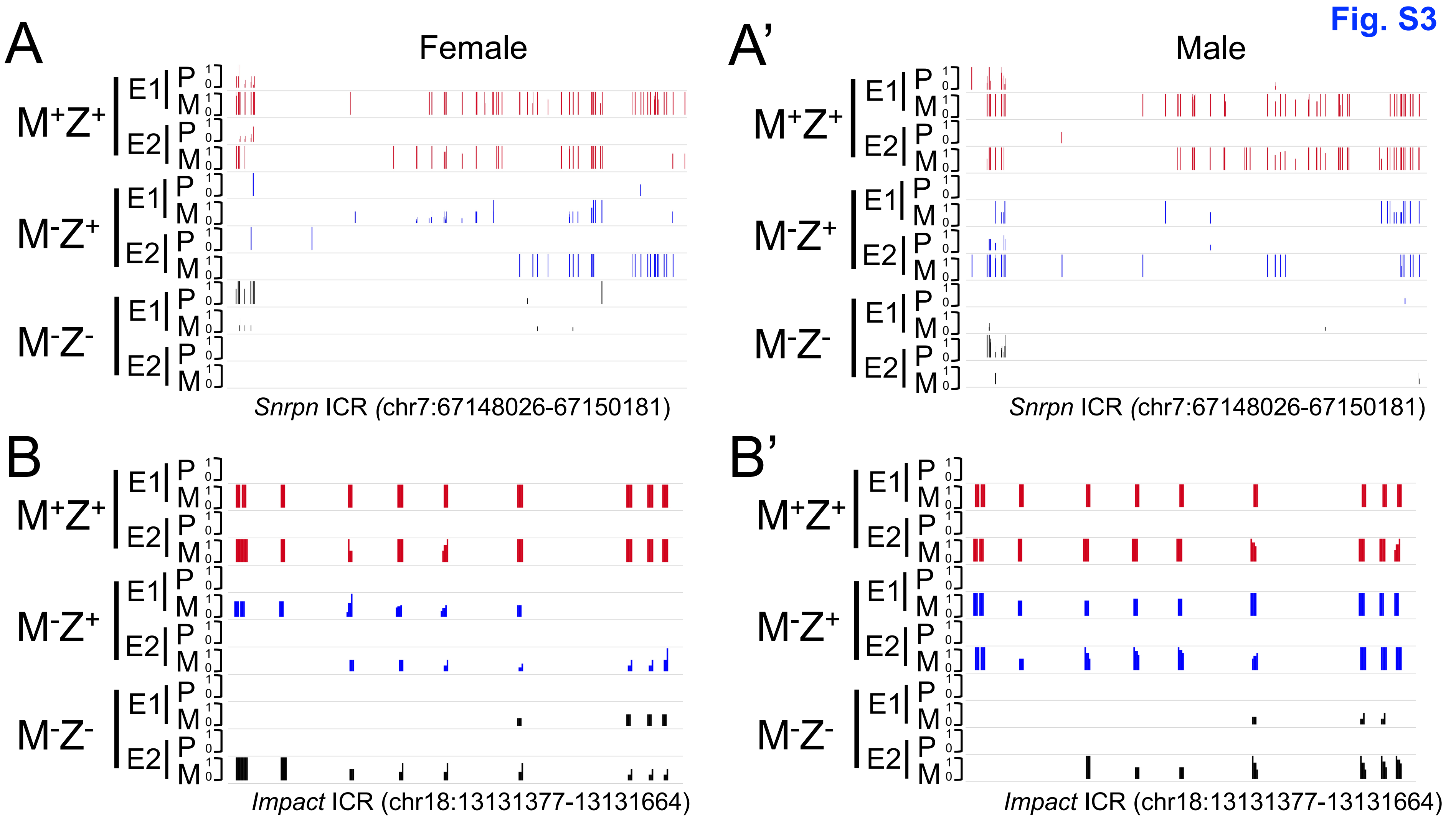

Fig. S3
A
Female
A'
Male
M+Z+
M-Z+
M-Z-
E1
E2
P
M
1
0
Snrpn ICR (chr7:67148026-67150181)
B
B'
Impact ICR (chr18:13131377-13131664)

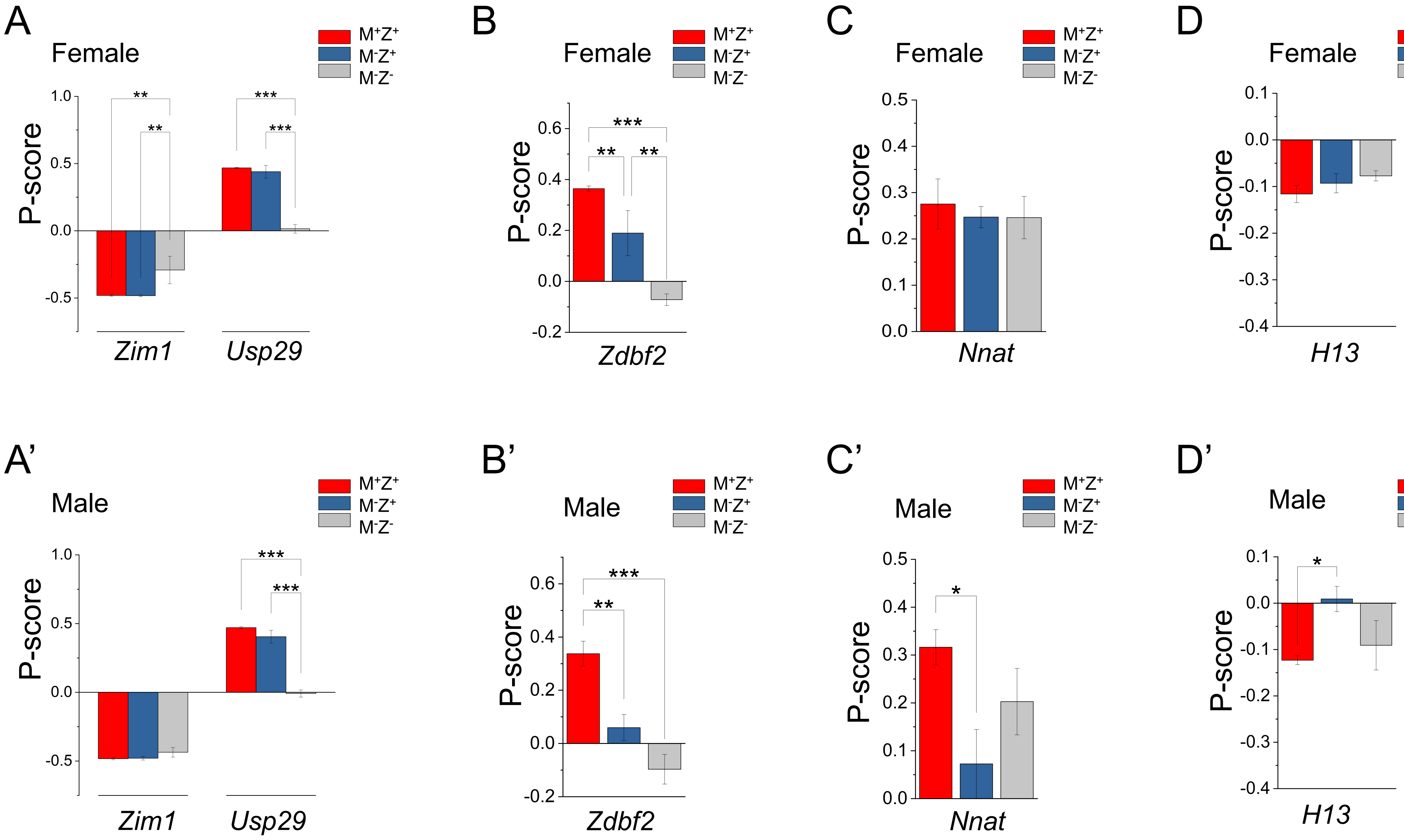
A
Female
M+Z+
M-Z+
M-Z-
P-score
1.0
0.5
0.0
-0.5
**
**
***
***
Zim1
Usp29
B
Female
P-score
0.6
0.4
0.2
0.0
-0.2
***
**
**
Zdbf2
C
Female
P-score
0.5
0.4
0.3
0.2
0.1
0.0
Nnat
D
Female
P-score
0.1
0.0
-0.1
-0.2
-0.3
-0.4
H13
A'
Male
P-score
***
***
Zim1
Usp29
B'
Male
P-score
***
**
Zdbf2
C'
Male
P-score
*
Nnat
D'
Male
P-score
*
H13

Fig. S4

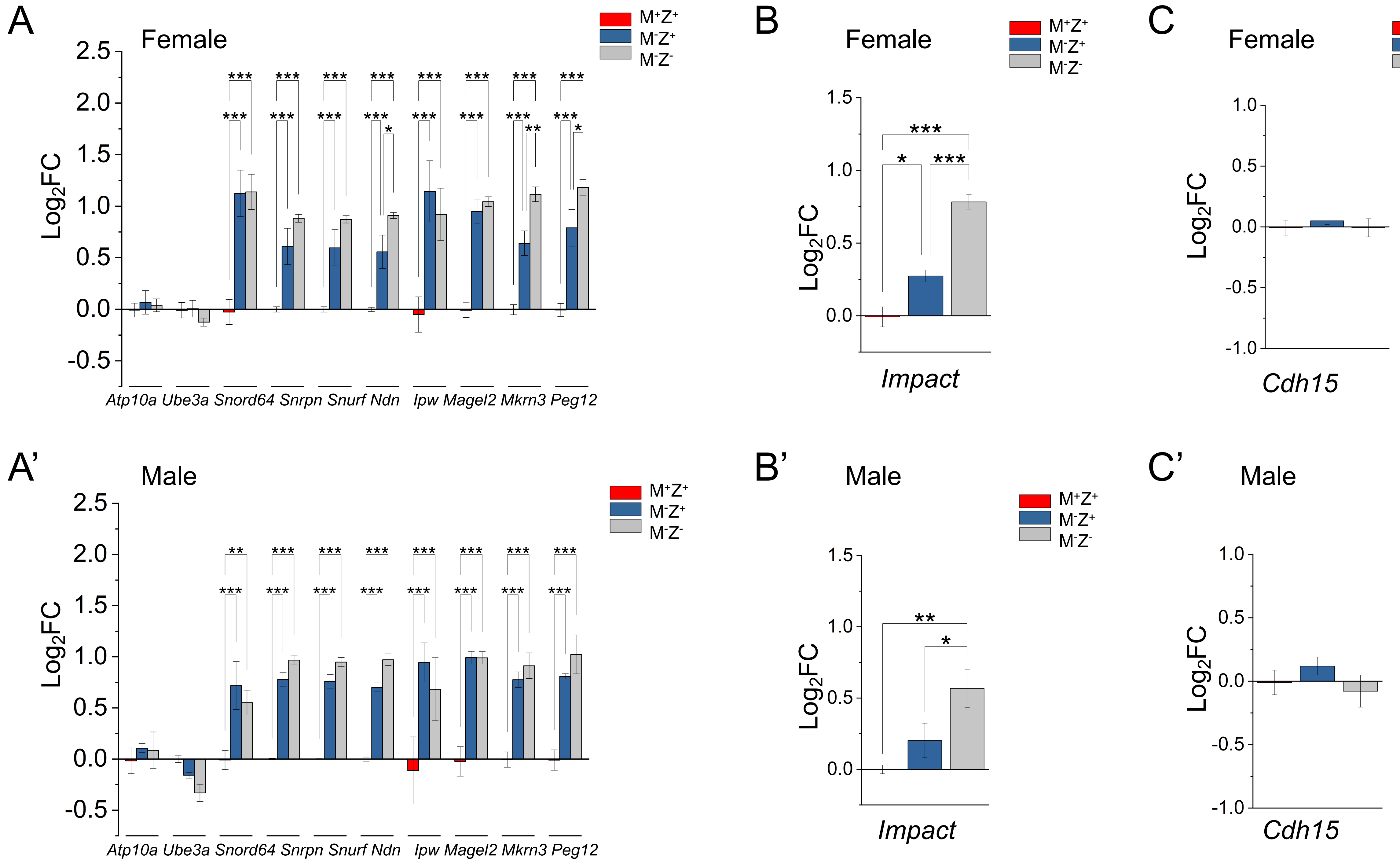
A
Female
M+Z+
M-Z+
M-Z-
Log2FC
Atp10a Ube3a Snord64 Snrpn Snurf Ndn Ipw Magel2 Mkrn3 Peg12
B
Female
Impact
C
Female
Cdh15
A'
Male
Atp10a Ube3a Snord64 Snrpn Snurf Ndn Ipw Magel2 Mkrn3 Peg12
B'
Male
Impact
C'
Male
Cdh15

Fig. S5

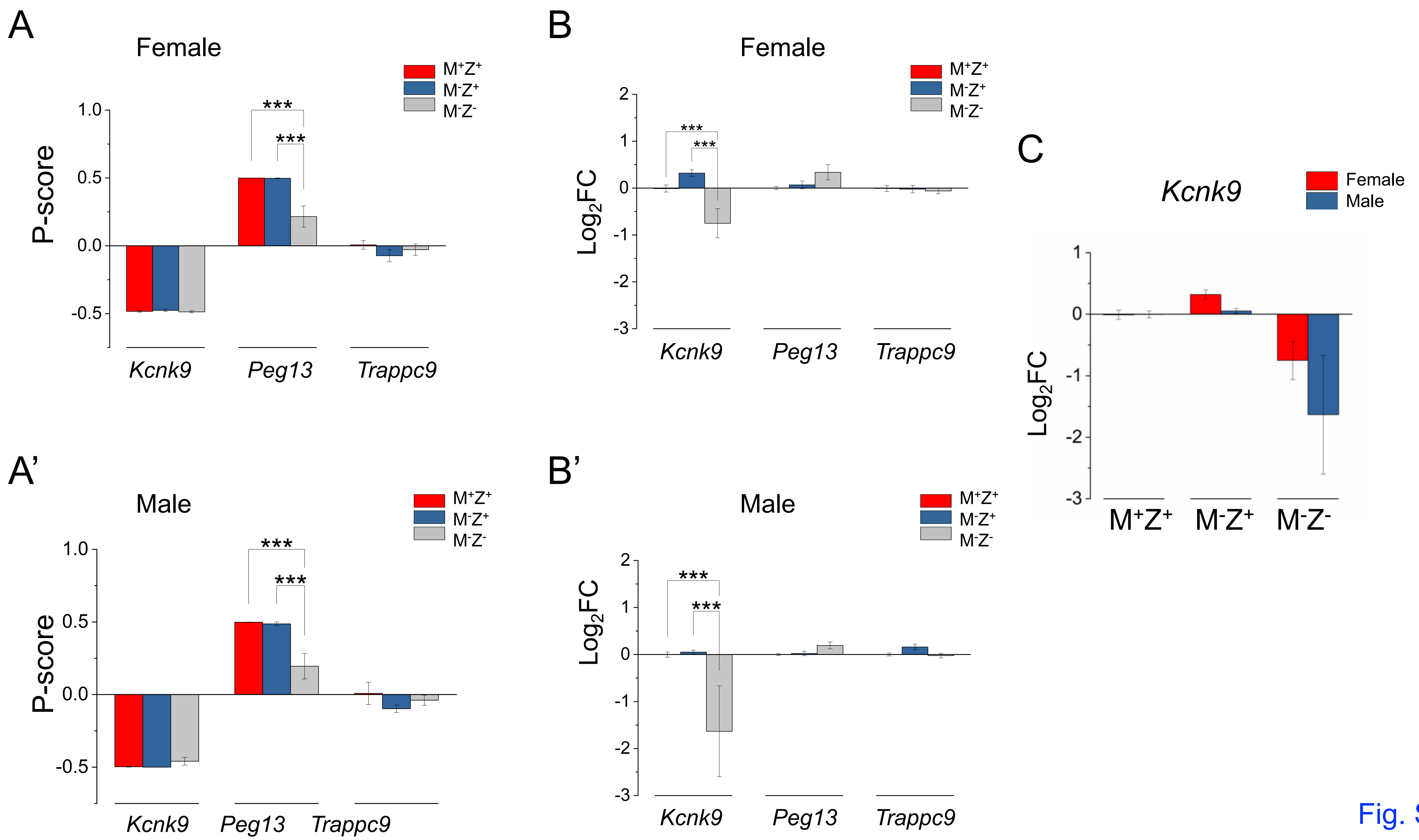

A
Female
M+Z+
M-Z+
M-Z-
P-score
1.0
0.5
0.0
-0.5
***
***
Kcnk9
Peg13
Trappc9
B
Female
Log2FC
2
1
0
-1
-2
-3
C
Kcnk9
Female
Male
M+Z+
M-Z+
M-Z-
A'
Male
B'
Male

Fig. S6

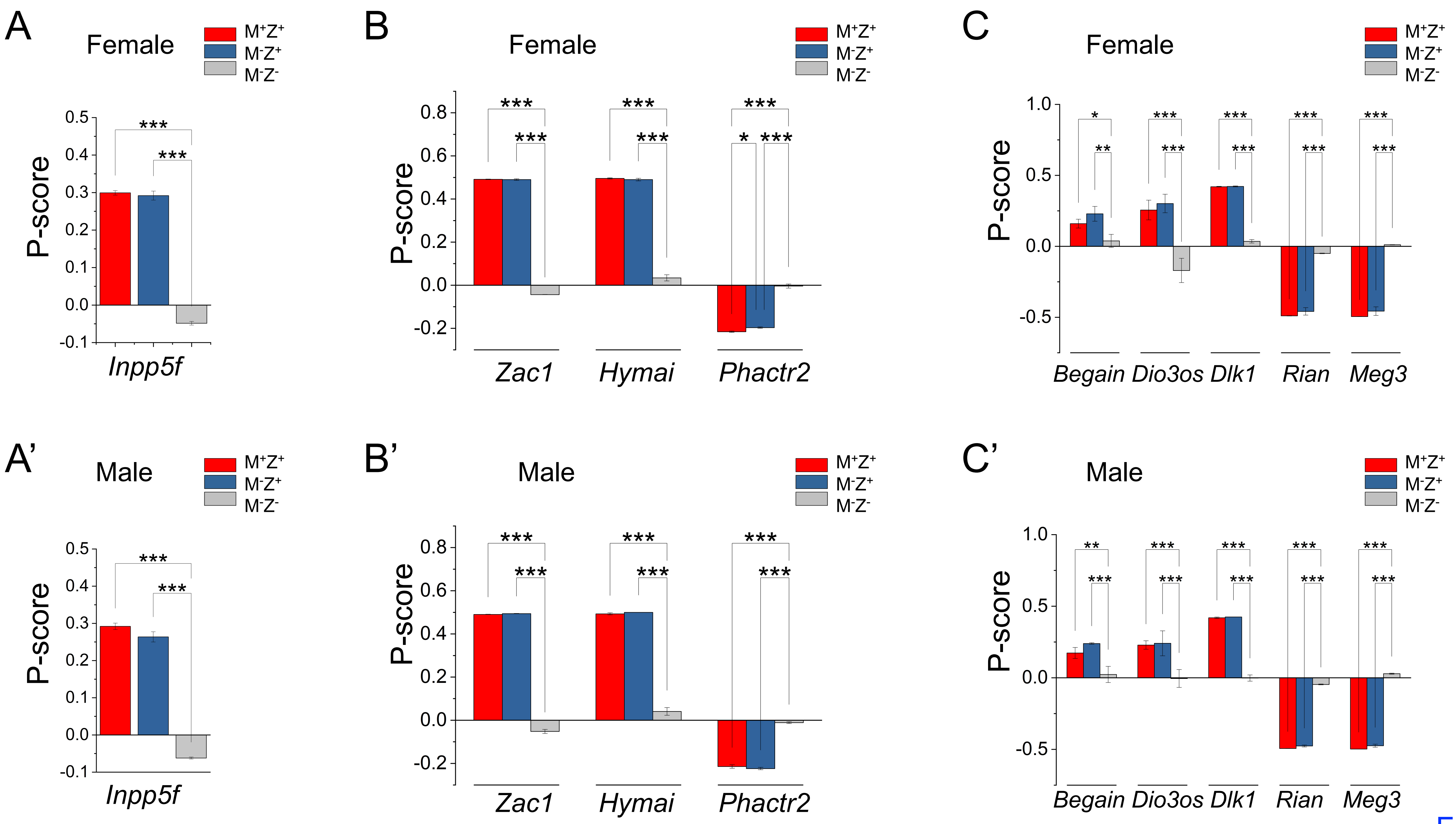
A
Female
M+Z+
M-Z+
M-Z-
P-score
0.5
0.4
0.3
0.2
0.1
0.0
-0.1
***
***
Inpp5f
B
Female
M+Z+
M-Z+
M-Z-
P-score
0.8
0.6
0.4
0.2
0.0
-0.2
***
*
Zac1
Hymai
Phactr2
C
Female
M+Z+
M-Z+
M-Z-
P-score
1.0
0.5
0.0
-0.5
**
Begain
Dio3os
Dlk1
Rian
Meg3
A'
Male
B'
Male
C'
Male

Fig. S7

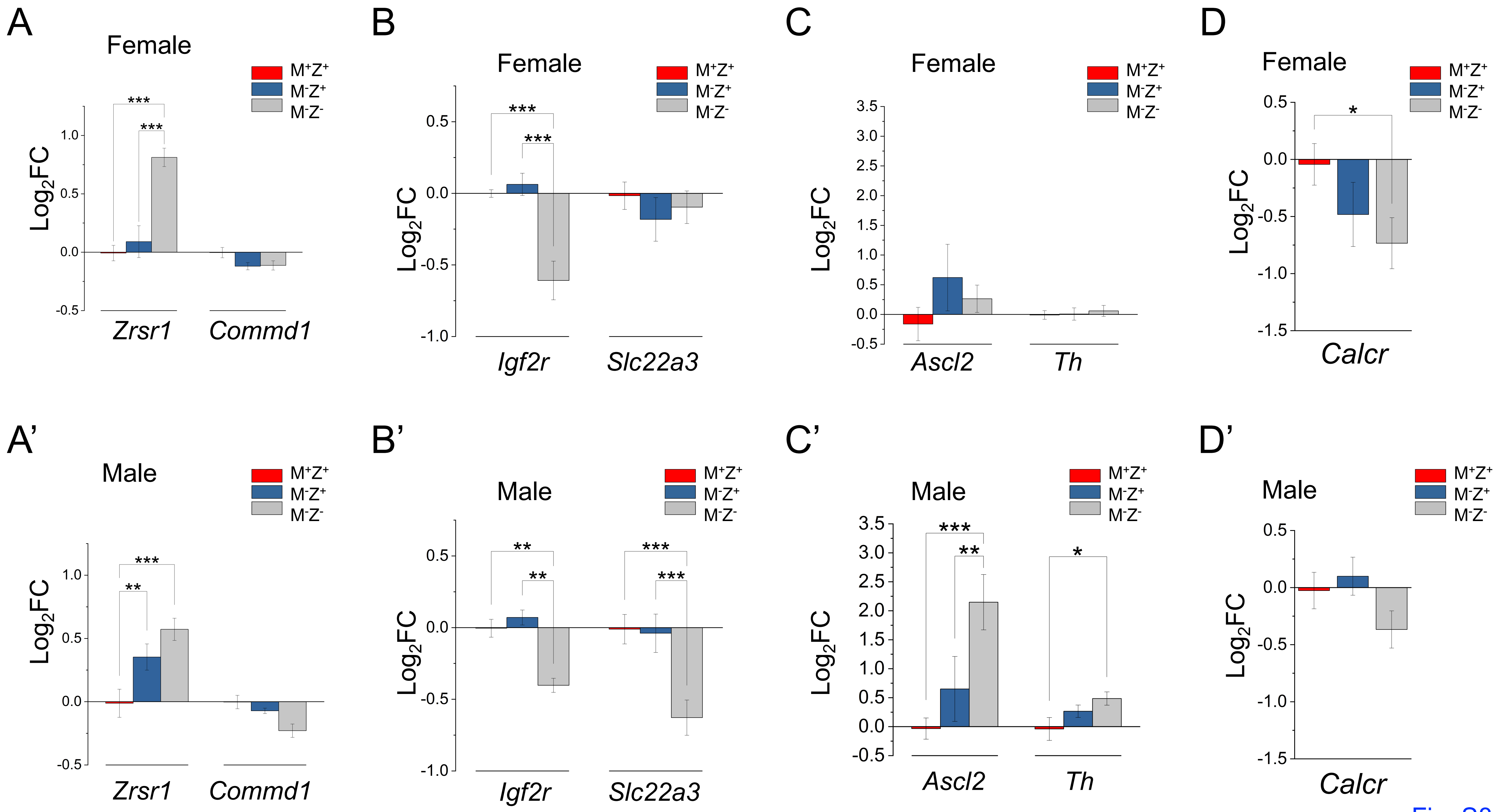

A
Female
M+Z+
M-Z+
M-Z-
Log2FC
Zrsr1
Commd1
B
Female
Igf2r
Slc22a3
C
Female
Ascl2
Th
D
Female
Calcr
A'
Male
B'
Male
C'
Male
D'
Male

Fig. S8

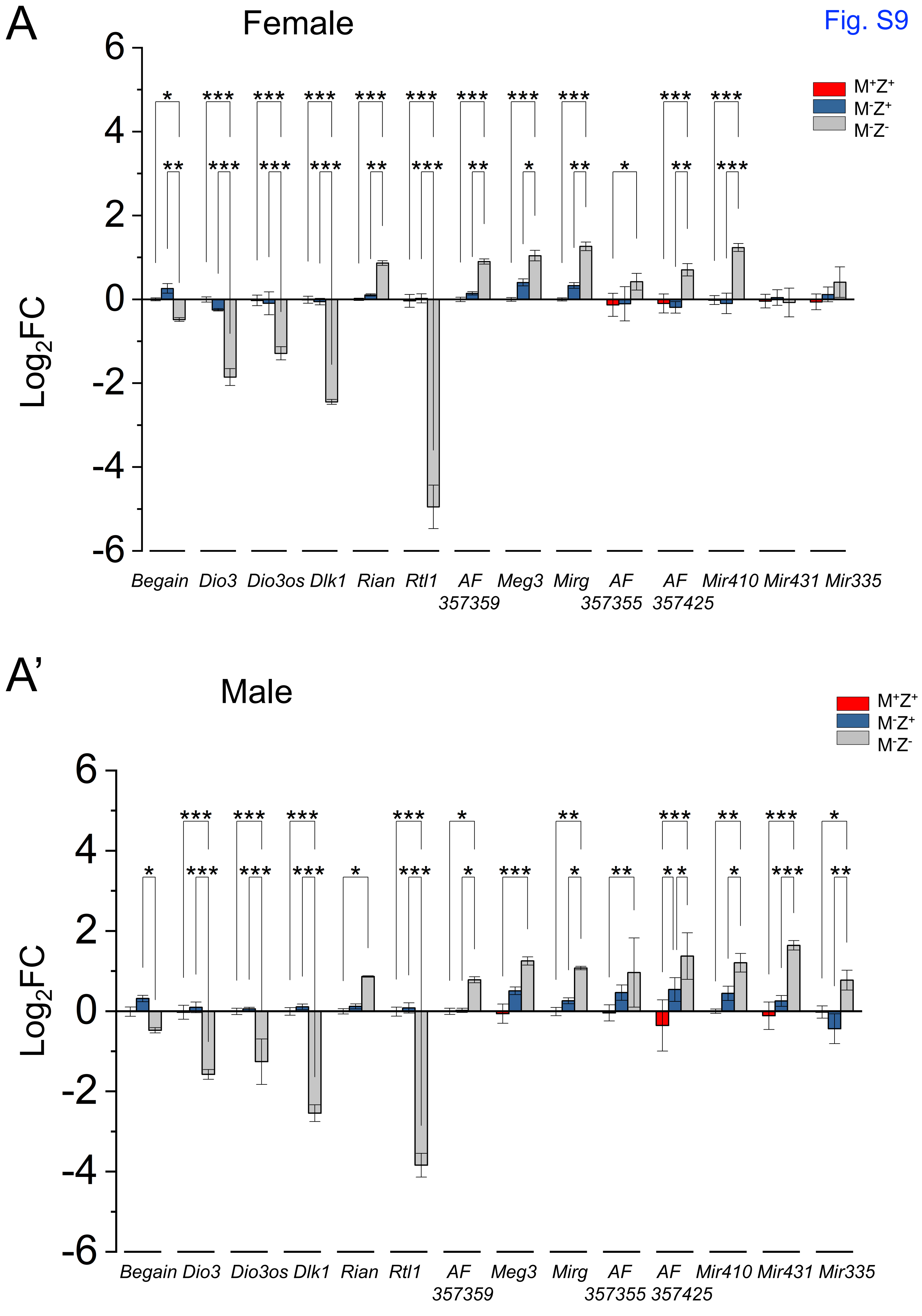

Fig. S9
A
Female
M+Z+
M-Z+
M-Z-
Log2FC
6
4
2
0
-2
-4
-6
Begain Dio3 Dio3os Dlk1 Rian Rtl1 AF 357359 Meg3 Mirg AF 357355 AF 357425 Mir410 Mir431 Mir335
A'
Male
M+Z+
M-Z+
M-Z-
Log2FC
6
4
2
0
-2
-4
-6
Begain Dio3 Dio3os Dlk1 Rian Rtl1 AF 357359 Meg3 Mirg AF 357355 AF 357425 Mir410 Mir431 Mir335

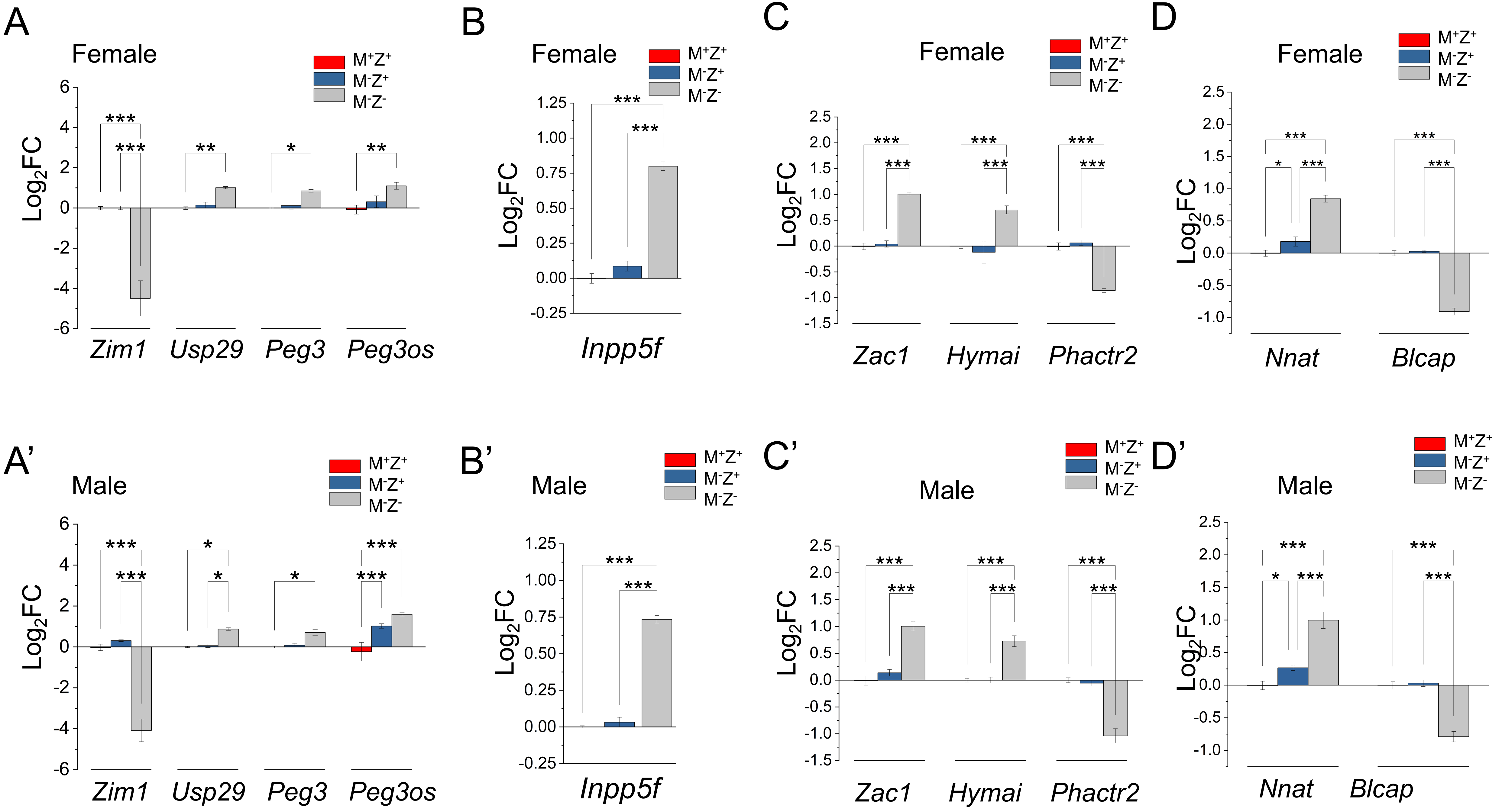
A
Female
M+Z+
M-Z+
M-Z-
Log2FC
Zim1
Usp29
Peg3
Peg3os
B
Female
Inpp5f
C
Female
Zac1
Hymai
Phactr2
D
Female
Nnat
Blcap
A'
Male
Zim1
Usp29
Peg3
Peg3os
B'
Male
Inpp5f
C'
Male
Zac1
Hymai
Phactr2
D'
Male
Nnat
Blcap

Fig. S10

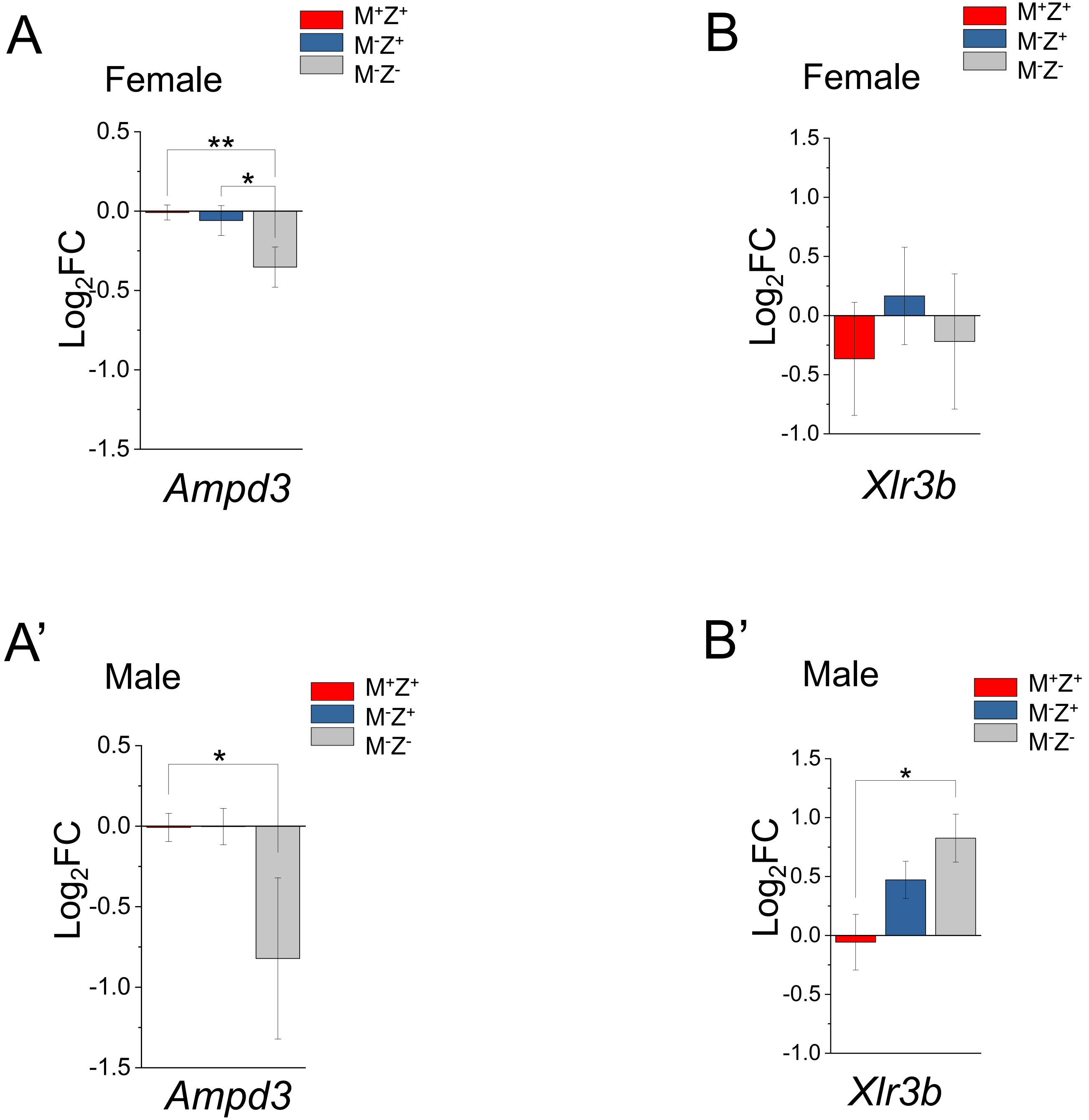
A
Female
M+Z+
M-Z+
M-Z-
Log2FC
0.5
0.0
-0.5
-1.0
-1.5
**
*
Ampd3
B
Female
M+Z+
M-Z+
M-Z-
Log2FC
1.5
1.0
0.5
0.0
-0.5
-1.0
Xlr3b
A'
Male
M+Z+
M-Z+
M-Z-
Log2FC
0.5
0.0
-0.5
-1.0
-1.5
*
Ampd3
B'
Male
M+Z+
M-Z+
M-Z-
Log2FC
1.5
1.0
0.5
0.0
-0.5
-1.0
*
Xlr3b

Fig. S11

Table S1. Allelic ICR methylation percentage is shown for *Snrpn* and *Impact* imprinted regions in the female embryos.

% of ICR methylation on the paternal (P) or maternal (M) chromosome in the female embryos

	$M^{+}Z^{+}$				$M^{-}Z^{+}$				$M^{-}Z^{-}$			
	Emb1		Emb2		Emb1		Emb2		Emb1		Emb2	
	P	M	P	M	P	M	P	M	P	M	P	M
Snrpn	5.32%	91.55%	2.54%	94.17%	4.55%	17.44%	9.09%	58.54%	16.49%	2.96%	0.00%	0.00%
Impact	0.00%	100.00%	0.00%	92.11%	0.00%	39.03%	NA	31.94%	0.00%	36.67%	0.00%	53.95%

Note: NA, not applicable. NA means here that methylation can not be calculated because there are no reads with the SNP for the ICR of this sample.

Reads of the ICRs on the paternal (P) or maternal (M) chromosome in the female embryos

	$M^{+}Z^{+}$				$M^{-}Z^{+}$				$M^{-}Z^{-}$			
	Emb1		Emb2		Emb1		Emb2		Emb1		Emb2	
	P	M	P	M	P	M	P	M	P	M	P	M
Snrpn	58	69	59	30	55	72	22	41	46	63	41	48
Impact	17	15	14	19	18	12	NA	12	16	5	4	19

Note: NA, not applicable. NA means here that there are no reads with the SNP for the ICR of the either allele in a particular sample.

Table S2. Allelic ICR methylation percentage is shown for *Snrpn* and *Impact* imprinted regions in the male embryos.

% of ICR methylation on the paternal (P) or maternal (M) chromosome in the male embryos

	$M^{+}Z^{+}$				$M^{-}Z^{+}$				$M^{-}Z^{-}$			
	Emb1		Emb2		Emb1		Emb2		Emb1		Emb2	
	P	M	P	M	P	M	P	M	P	M	P	M
Snrpn	12.88%	95.58%	3.45%	93.37%	0.00%	41.67%	5.98%	92.22%	0.53%	0.86%	20.73%	2.13%
Impact	0.00%	100.00%	0.00%	95.10%	0.00%	83.33%	0.00%	86.27%	3.33%	13.33%	0.00%	72.42%

Reads of the ICRs on the paternal (P) or maternal (M) chromosome in the male embryos

	$M^{+}Z^{+}$				$M^{-}Z^{+}$				$M^{-}Z^{-}$			
	Emb1		Emb2		Emb1		Emb2		Emb1		Emb2	
	P	M	P	M	P	M	P	M	P	M	P	M
Snrpn	62	63	29	52	42	36	46	30	47	58	41	47
Impact	14	10	6	17	11	13	12	17	15	10	7	11

Table S3. Allelic expression of some imprinted genes is shown with the calculated P-score of the imprinted gene based on the sequence reads of the paternal (P) and maternal (M) alleles in the female embryos.

Reads of the imprinted genes on the paternal (P) or maternal (M) chromosome in the female embryos

		M^+Z^+												M^-Z^+						M^-Z^-							
		Emb 1		Emb 2		Emb 3		Emb 4		Emb 5		Emb 6		Emb 1		Emb 2		Emb 3		Emb 1		Emb 2		Emb 3		Emb 4	
**ICR**	Gene	P	M	P	M	P	M	P	M	P	M	P	M	P	M	P	M	P	M	P	M	P	M	P	M	P	M
	Kcnk9	1	65	2	63	1	60	3	79	0	92	0	85	1	91	2	92	3	74	2	89	0	24	1	32	0	35
Peg13	*Peg13*	404	0	367	0	363	0	359	0	397	0	341	1	366	0	361	0	409	3	363	21	338	219	351	208	328	153
	Trappc9	28	20	26	27	26	16	21	31	26	29	25	28	26	31	16	31	28	30	41	37	16	28	16	20	27	22
	Atp10a	36	35	32	28	37	23	23	38	39	50	39	40	35	40	25	35	31	43	39	55	29	40	37	39	37	30
	Ube3a	594	662	519	710	495	611	565	706	733	832	656	790	640	613	518	620	643	731	707	657	683	642	542	526	626	637
	Snord64	7	0	6	0	9	0	10	0	25	0	8	0	10	27	15	11	13	2	10	20	11	23	7	11	16	7
Snrpn	*Snrpn*	684	1	693	2	674	8	712	5	724	15	676	2	609	706	636	265	718	254	701	829	656	744	673	681	632	751
	Ndn	841	31	804	9	775	28	795	30	875	14	895	20	785	732	663	301	879	276	939	983	842	803	742	811	785	876
	Magel2	79	0	77	1	74	1	92	3	102	3	84	8	89	91	101	61	87	26	88	84	82	50	97	75	81	95
	Mkrn3	90	0	59	0	46	2	77	2	64	0	60	2	55	63	51	53	74	25	92	86	97	69	83	51	77	67
	Peg12	134	8	117	3	98	4	127	6	112	2	129	4	116	88	99	41	143	28	187	142	168	169	127	128	128	120
Impact	*Impact*	489	69	492	67	393	63	465	75	552	79	499	90	533	76	534	87	525	128	641	294	590	354	555	430	610	483
Peg3	*Zim1*	1	311	10	392	10	338	2	319	4	377	14	365	12	390	5	328	2	312	5	64	1	4	2	31	3	3
	Usp29	205	8	166	5	150	4	172	8	262	8	223	6	200	3	170	2	212	39	212	156	172	187	201	156	186	235
Inpp5f	*Inpp5f*	685	162	633	167	555	163	613	144	702	165	651	160	567	171	706	176	710	170	748	889	614	748	610	782	706	829
Zdbf2	*Zdbf2*	53	8	52	7	34	4	47	7	70	13	76	16	72	12	40	32	59	31	55	74	64	94	49	77	62	64
	Begain	17	11	20	13	20	11	21	8	25	7	19	13	37	11	25	15	34	9	17	18	15	10	14	18	17	10
	Dio3os	17	7	20	5	15	0	9	10	11	3	13	4	13	1	16	5	15	6	2	7	6	5	1	5	5	8
**IG-DMR**	*Dlk1*	1595	143	2152	208	2120	181	1504	113	1648	152	1737	143	1660	133	1864	152	1417	131	230	198	152	152	202	162	248	204
	Rian	23	3219	53	3417	39	3216	41	3350	29	3877	25	3619	50	3342	58	3711	345	3336	3106	3705	2484	3049	2450	3047	3371	4139
	Meg3	4	4358	19	3811	28	3789	20	3772	33	4336	25	4235	58	4293	68	5155	502	4352	4117	3875	3764	3649	3679	3539	5745	5486
	Phactr2	334	836	334	888	338	806	388	960	444	1156	394	992	408	912	378	891	440	1017	362	401	375	389	370	342	377	379
Zac1	*Hymai*	230	0	210	1	209	1	174	3	262	0	258	0	224	2	140	3	242	0	225	195	165	163	182	136	149	135
	Plagl1	5292	47	5833	56	5583	90	5419	39	6600	36	6810	43	6028	31	5246	94	6166	45	7056	8471	5711	6780	5304	6301	6412	7625
Peg5	*Nnat*	16	4	12	0	9	2	7	3	11	7	13	5	18	6	12	5	22	6	34	7	27	11	19	11	30	7
Mcts2	*H13*	148	195	132	205	111	186	108	232	144	262	159	207	137	215	132	212	159	196	167	202	146	199	143	213	156	221

P-scores of the imprinted genes in the female embryos

		M^+Z^+						M^-Z^+			M^-Z^-			
		Emb 1	Emb 2	Emb 3	Emb 4	Emb 5	Emb 6	Emb 1	Emb 2	Emb 3	Emb 1	Emb 2	Emb 3	Emb 4
**ICR**	Gene	P-score	P-score	P-score	P-score	P-score	P-score	P-score	P-score	P-score	P-score	P-score	P-score	P-score
	Kcnk9	-0.48	-0.47	-0.48	-0.46	-0.5	-0.5	-0.49	-0.48	-0.46	-0.48	-0.5	-0.47	-0.5
Peg13	*Peg13*	0.5	0.5	0.5	0.5	0.5	0.5	0.5	0.5	0.49	0.45	0.11	0.13	0.18
	Trappc9	0.08	-0.01	0.12	-0.1	-0.03	-0.03	-0.04	-0.16	-0.02	0.03	-0.14	-0.06	0.05
	Atp10a	0.01	0.03	0.12	-0.12	-0.06	-0.01	-0.03	-0.08	-0.08	-0.09	-0.08	-0.01	0.05
	Ube3a	-0.03	-0.08	-0.05	-0.06	-0.03	-0.05	0.01	-0.04	-0.03	0.02	0.02	0.01	-0
	Snord64	0.5	0.5	0.5	0.5	0.5	0.5	-0.23	0.08	0.37	-0.17	-0.18	-0.11	0.2
Snrpn	*Snrpn*	0.5	0.5	0.49	0.49	0.48	0.5	-0.04	0.21	0.24	-0.04	-0.03	-0	-0.04
	Ndn	0.46	0.49	0.47	0.46	0.48	0.48	0.02	0.19	0.26	-0.01	0.01	-0.02	-0.03
	Magel2	0.5	0.49	0.49	0.47	0.47	0.41	-0.01	0.12	0.27	0.01	0.12	0.06	-0.04
	Mkrn3	0.5	0.5	0.46	0.47	0.5	0.47	-0.03	-0.01	0.25	0.02	0.08	0.12	0.03
	Peg12	0.44	0.48	0.46	0.45	0.48	0.47	0.07	0.21	0.34	0.07	-0	-0	0.02
Impact	*Impact*	0.38	0.38	0.36	0.36	0.37	0.35	0.38	0.36	0.3	0.19	0.13	0.06	0.06
Peg3	*Zim1*	-0.5	-0.48	-0.47	-0.49	-0.49	-0.46	-0.47	-0.48	-0.49	-0.43	-0.3	-0.44	0
	Usp29	0.46	0.47	0.47	0.46	0.47	0.47	0.49	0.49	0.34	0.08	-0.02	0.06	-0.06
Inpp5f	*Inpp5f*	0.31	0.29	0.27	0.31	0.31	0.3	0.27	0.3	0.31	-0.04	-0.05	-0.06	-0.04
Zdbf2	*Zdbf2*	0.37	0.38	0.39	0.37	0.34	0.33	0.36	0.06	0.16	-0.07	-0.09	-0.11	-0.01
	Begain	0.11	0.11	0.15	0.22	0.28	0.09	0.27	0.13	0.29	-0.01	0.1	-0.06	0.13
	Dio3os	0.21	0.3	0.5	-0.03	0.29	0.26	0.43	0.26	0.21	-0.28	0.05	-0.33	-0.12
**IG-DMR**	*Dlk1*	0.42	0.41	0.42	0.43	0.42	0.42	0.43	0.42	0.42	0.04	0	0.05	0.05
	Rian	-0.49	-0.48	-0.49	-0.49	-0.49	-0.49	-0.49	-0.48	-0.41	-0.04	-0.05	-0.05	-0.05
	Meg3	-0.5	-0.5	-0.49	-0.49	-0.49	-0.49	-0.49	-0.49	-0.4	0.02	0.01	0.01	0.01
	Phactr2	-0.21	-0.23	-0.2	-0.21	-0.22	-0.22	-0.19	-0.2	-0.2	-0.03	-0.01	0.02	-0
Zac1	*Hymai*	0.5	0.5	0.5	0.48	0.5	0.5	0.49	0.48	0.5	0.04	0	0.07	0.02
	Plagl1	0.49	0.49	0.48	0.49	0.49	0.49	0.49	0.48	0.49	-0.05	-0.04	-0.04	-0.04
Peg5	*Nnat*	0.3	0.5	0.32	0.2	0.11	0.22	0.25	0.21	0.29	0.33	0.21	0.13	0.31
Mcts2	*H13*	-0.07	-0.11	-0.13	-0.18	-0.15	-0.07	-0.11	-0.12	-0.05	-0.05	-0.08	-0.1	-0.09

Table S4. Allelic expression of some imprinted genes is shown with the calculated P-score of the imprinted gene based on the sequence reads of the paternal (P) and maternal (M) alleles in the male embryos.

Reads of the imprinted genes on the paternal (P) or maternal (M) chromosome in the male embryos

		M^+Z^+								M^-Z^+						M^-Z^-					
		Emb 1		Emb 2		Emb 3		Emb 4		Emb 1		Emb 2		Emb 3		Emb 1		Emb 2		Emb 3	
**ICR**	Gene	P	M	P	M	P	M	P	M	P	M	P	M	P	M	P	M	P	M	P	M
**Peg13**	*Kcnk9*	0	71	0	46	1	80	0	68	0	74	0	63	0	64	1	32	0	6	4	40
	Peg13	507	0	313	1	377	1	368	0	371	15	374	0	377	0	301	118	227	199	344	68
	Trappc9	21	52	25	15	26	18	28	25	15	22	15	27	32	40	32	30	15	23	25	28
**Snrpn**	*Atp10a*	51	32	30	25	35	31	43	32	20	36	28	24	36	38	31	13	34	39	41	38
	Ube3a	788	987	558	631	520	635	470	596	460	483	404	502	454	496	482	448	400	408	393	399
	Snord64	20	0	14	0	15	0	9	0	14	15	5	10	11	12	8	10	6	15	4	7
	Snrpn	911	5	678	4	734	1	637	3	567	365	636	469	670	380	595	540	671	816	583	607
	Ndn	1147	5	691	4	925	14	795	4	781	502	799	600	796	489	662	687	878	908	857	898
	Magel2	106	0	71	1	70	1	128	0	85	69	99	60	99	42	57	68	72	74	82	95
	Mkrn3	99	1	58	0	76	0	68	1	63	29	65	43	59	44	76	76	53	43	53	73
	Peg12	170	3	90	0	103	1	137	2	114	70	125	82	118	67	129	118	97	76	109	136
Impact	*Impact*	646	87	474	63	507	63	467	66	396	69	382	237	439	151	471	324	434	330	386	210
**Peg3**	*Zim1*	2	367	11	269	2	333	7	399	15	316	1	370	5	382	2	26	0	6	3	22
	Usp29	259	10	173	2	165	4	148	7	116	27	158	9	154	7	114	142	145	149	122	106
Inpp5f	*Inpp5f*	833	223	553	165	633	147	599	152	530	154	510	144	497	178	477	622	525	678	545	685
Zdbf2	*Zdbf2*	79	14	37	6	41	3	46	19	38	29	32	36	41	23	26	62	54	58	38	50
**IG-DMR**	*Begain*	18	13	16	9	23	8	27	10	36	13	22	8	30	10	8	7	16	10	8	11
	Dio3os	17	6	15	4	17	6	11	6	13	5	18	2	12	8	4	4	3	2	5	8
	Dlk1	2411	182	1734	181	2312	194	1932	172	1890	152	1722	139	1765	145	137	119	150	151	205	240
	Rian	25	4000	14	3154	21	3183	30	3445	122	3410	31	2948	81	3082	2243	2675	2577	3065	2282	2818
	Meg3	4	4426	6	3881	6	3264	23	5643	197	5177	18	4318	172	4660	4663	4200	4634	4207	3916	3392
**Zac1**	*Phactr2*	514	1240	367	943	329	910	390	895	324	823	278	773	317	811	317	343	264	270	287	297
	Hymai	276	2	188	4	211	0	175	0	227	0	191	0	201	0	133	98	144	134	188	168
	Plagl1	7235	63	5131	64	5957	59	6560	51	6173	41	5418	22	6065	41	4418	5877	5173	6156	6206	7345
Peg5	*Nnat*	15	6	16	2	10	2	29	6	13	14	20	8	12	11	30	23	42	14	19	5
Mcts2	*H13*	187	308	136	201	119	212	110	190	119	134	147	150	182	142	137	160	82	189	114	132

P-scores of the imprinted genes in the male embryos

		M^+Z^+				M^-Z^+			M^-Z^-		
		Emb 1	Emb 2	Emb 3	Emb 4	Emb 1	Emb 2	Emb 3	Emb 1	Emb 2	Emb 3
**ICR**	Gene	P-score	P-score	P-score	P-score	P-score	P-scor	P-score	P-score	P-score	P-score
**Peg13**	*Kcnk9*	-0.5	-0.5	-0.49	-0.5	-0.5	-0.5	-0.5	-0.47	-0.5	-0.41
	Peg13	0.5	0.497	0.497	0.5	0.461	0.5	0.5	0.218	0.033	0.335
	Trappc9	-0.21	0.125	0.091	0.028	-0.09	-0.14	-0.06	0.016	-0.11	-0.03
**Snrpn**	*Atp10a*	0.114	0.045	0.03	0.073	-0.14	0.038	-0.01	0.205	-0.03	0.019
	Ube3a	-0.06	-0.03	-0.05	-0.06	-0.01	-0.05	-0.02	0.018	-0	-0
	Snord64	0.5	0.5	0.5	0.5	-0.02	-0.17	-0.02	-0.06	-0.21	-0.14
	Snrpn	0.495	0.494	0.499	0.495	0.108	0.076	0.138	0.024	-0.05	-0.01
	Ndn	0.496	0.494	0.485	0.495	0.109	0.071	0.119	-0.01	-0.01	-0.01
	Magel2	0.5	0.486	0.486	0.5	0.052	0.123	0.202	-0.04	-0.01	-0.04
	Mkrn3	0.49	0.5	0.5	0.486	0.185	0.102	0.073	0	0.052	-0.08
	Peg12	0.483	0.5	0.49	0.486	0.12	0.104	0.138	0.022	0.061	-0.06
Impact	*Impact*	0.381	0.383	0.389	0.376	0.352	0.117	0.244	0.092	0.068	0.148
**Peg3**	*Zim1*	-0.49	-0.46	-0.49	-0.48	-0.45	-0.5	-0.49	-0.43	-0.5	-0.38
	Usp29	0.463	0.489	0.476	0.455	0.311	0.446	0.457	-0.05	-0.01	0.035
Inpp5f	*Inpp5f*	0.289	0.27	0.312	0.298	0.275	0.28	0.236	-0.07	-0.06	-0.06
Zdbf2	*Zdbf2*	0.349	0.36	0.432	0.208	0.067	-0.03	0.141	-0.2	-0.02	-0.07
**IG-DMR**	*Begain*	0.081	0.14	0.242	0.23	0.235	0.233	0.25	0.033	0.115	-0.08
	Dio3os	0.239	0.289	0.239	0.147	0.222	0.4	0.1	0	0.1	-0.12
	Dlk1	0.43	0.405	0.423	0.418	0.426	0.425	0.424	0.035	-0	-0.04
	Rian	-0.49	-0.5	-0.49	-0.49	-0.47	-0.49	-0.47	-0.04	-0.04	-0.05
	Meg3	-0.5	-0.5	-0.5	-0.5	-0.46	-0.5	-0.46	0.026	0.024	0.036
**Zac1**	*Phactr2*	-0.21	-0.22	-0.23	-0.2	-0.22	-0.24	-0.22	-0.02	-0.01	-0.01
	Hymai	0.493	0.479	0.5	0.5	0.5	0.5	0.5	0.076	0.018	0.028
	Plagl1	0.491	0.488	0.49	0.492	0.493	0.496	0.493	-0.07	-0.04	-0.04
Peg5	*Nnat*	0.214	0.389	0.333	0.329	-0.02	0.214	0.022	0.066	0.25	0.292
Mcts2	*H13*	-0.12	-0.1	-0.14	-0.13	-0.03	-0.01	0.062	-0.04	-0.2	-0.04

Table S5. Transcript per million (TPM) values are used to measure the expression levels of some imprinted genes in the female embryos.

TPMs of the imprinted genes in the female embryos

		M^+Z^+						M^-Z^+			M^-Z^-			
ICR	Gene	Emb 1	Emb 2	Emb 3	Emb 4	Emb 5	Emb 6	Emb 1	Emb 2	Emb 3	Emb 1	Emb 2	Emb 3	Emb 4
**Peg13**	*Kcnk9*	2.96791	2.69637	2.99391	3.40285	3.88433	3.25281	3.65612	4.0269	4.32981	3.22365	1.1695	1.6153	2.1479
	Peg13	46.1575	45.7904	44.4469	44.4767	51.7617	46.207	48.9431	44.0311	53.6505	46.0617	70.9372	71.5135	50.9483
	Trappc9	9.82761	9.88802	9.4506	12.1726	11.7162	11.6736	10.3651	9.79158	11.8268	11.6606	10.3514	9.81384	9.66456
**Snrpn**	*Atp10a*	2.83774	2.7107	3.13966	2.90228	3.58672	3.4739	3.3257	2.81155	3.6871	3.35651	3.40703	3.22829	2.81545
	Ube3a	53.1838	54.4557	48.456	59.0664	67.6303	65.1971	61.5554	52.1088	61.483	55.2146	56.219	51.3116	50.2816
	Snord64	44.8086	40.6234	53.3255	53.915	73.7038	48.9098	151.428	112.336	88.2243	144.897	135.022	86.2196	106.191
	Snrpn	46.6289	51.5885	52.0448	49.0813	51.0578	51.8816	97.9578	67.2621	68.7032	93.9082	93.5313	98.155	86.3677
	Snurf	92.8559	102.746	103.539	97.853	100.704	103.582	193.109	132.774	135.549	186.185	185.018	192.351	171.01
	Ndn	294.488	293.45	307.081	281.658	290.061	308.58	544.496	387.649	390.914	563.274	545.269	584.752	532.692
	Ipw	1.48668	1.52753	2.63836	1.1826	1.81462	2.21375	5.62374	4.12106	2.76452	2.38183	5.04458	4.18328	2.74882
	Magel2	11.5993	10.9293	12.1848	14.1507	14.6226	14.1293	28.7208	25.1693	21.5179	24.6798	25.9535	28.7053	27.5158
	Mkrn3	8.85325	8.12363	7.76337	9.2967	7.56485	8.93146	15.4632	12.3034	11.885	19.1568	20.0082	18.1842	15.9224
	Peg12	23.1471	19.8193	18.4364	24.1433	21.6944	23.4833	47.9821	31.9863	34.8281	46.9348	57.4591	49.0432	45.1714
**Impact**	*Impact*	30.9197	31.0878	28.3161	34.2393	37.565	37.8329	39.2125	39.0681	42.6155	53.9927	54.1054	61.12	60.5502
**Cdh15**	*Cdh15*	19.6179	21.5591	19.5268	18.6683	19.8472	24.972	20.7807	22.3433	21.1891	19.4473	18.3596	22.0956	22.8219
**Peg3**	*Zim1*	55.2035	71.2386	71.3925	67.8288	81.0691	79.7219	81.1328	66.5278	67.1605	10.4866	0.92115	7.63759	1.33459
	Usp29	8.0595	7.52738	7.47615	8.26809	9.56301	9.64219	9.06548	7.89811	11.2143	15.4843	18.3552	17.7391	16.2538
	Peg3	287.266	273.437	263.879	291.965	328.377	312.14	327.394	252.071	386.652	499.6	592.687	543.257	483.519
	Peg3os	6.58798	3.88858	5.67162	8.02803	9.93876	10.8374	9.24585	6.55677	13.1954	17.811	20.9888	14.6427	12.0309
**Inpp5f**	*Inpp5f*	39.144	41.1691	37.7447	41.0036	44.5342	42.7468	41.8218	43.424	45.5292	73.3496	70.0503	74.608	67.9648
**Zdbf2**	*Zdbf2*	20.2171	20.3171	17.7901	21.3192	25.9547	24.3547	29.1928	26.7662	29.8895	36.7966	48.3575	43.9814	39.8783
**Zrsr1**	*Zrsr1*	9.31215	9.26296	8.651	6.98422	7.70932	8.01371	8.12677	7.99647	10.7003	12.7704	15.4754	16.3768	14.0895
	Commd1	99.4365	97.2291	87.2762	90.185	88.6472	81.1116	83.4597	86.5428	80.3372	77.987	88.138	86.6123	82.8783
**Peg10**	*Calcr*	1.02436	0.63788	1.45432	1.17465	1.42599	1.568	0.89167	0.61308	1.20134	0.81124	0.82564	0.91712	0.46217
**Zac1**	*Plagl1*	211.592	247.804	243.267	230.244	274.55	284.649	270.019	234.558	264.213	538.802	477.368	492.276	491.064
	Hymai	17.0503	16.7304	15.5762	14.4459	16.9557	17.8983	17.2469	11.2924	17.8093	29.3264	26.7659	28.4421	22.8722
	Phactr2	21.6426	22.6093	21.4174	24.9051	28.9388	26.7899	25.5221	23.8216	27.1402	13.3048	14.0288	13.8947	12.5193
**IG-DMR**	*Begain*	15.0731	16.4077	16.6719	14.6192	16.3754	14.6955	16.4506	21.62	18.4143	11.7936	10.7433	11.7952	10.4724
	Dio3	7.26962	7.00254	7.1878	5.77537	6.15796	7.49415	5.93872	5.53261	5.70294	2.69071	2.04613	1.43828	1.59173
	Dio3os	2.74915	3.1169	1.91788	2.13299	1.79876	2.44315	1.51284	2.66064	2.67725	1.02517	1.12065	0.70021	1.07935
	Dlk1	251.919	335.904	347.676	252.468	281.332	321.429	301.157	304.702	257.599	55.9618	48.9092	55.5527	59.0662
	Rian	473.334	512.411	527.923	488.603	533.396	519.419	529.35	564.29	548.253	918.154	853.686	910.763	1032.48
	Rtl1	3.75678	5.81941	5.21858	2.88655	4.53259	5.22928	4.54463	5.33243	4.12901	0.08593	0.18786	0.08049	0.36744
	AF357359	5302.53	5946.55	6360.52	5402.46	6421.53	6412.07	6591.62	6906.75	6286.17	10638.9	10487.1	10921.7	12679.2
	AF357426	66.9572	29.7501	32.7577	53.6736	52.4099	55.9664	44.2817	47.0282	49.2147	70.1261	90.919	47.7383	115.701
	Meg3	165.718	149.973	157.324	164.261	177.59	183.58	202.084	245.242	214.568	305.281	306.148	332.733	440.608
	Mirg	45.6426	45.5541	49.3954	51.0719	51.3609	52.5369	62.8588	66.3652	56.7235	107.5	109.328	113.756	146.003
	AF357355	49.5234	22.8101	21.8204	29.6912	11.9379	21.1254	41.787	21.3018	16.0761	43.0909	30.2812	45.0488	25.426
	AF357425	31.6296	19.8603	28.0661	23.3632	59.665	25.3606	26.1728	32.8789	23.8941	66.5099	51.9316	40.792	47.709
	Mir410	59.875	75.3623	63.755	93.3861	91.1874	75.6122	90.7739	77.7998	51.6684	167.272	160.731	180.062	216.315
	Mir431	7.9943	6.84497	11.3498	13.6269	13.306	10.9883	8.50513	12.4651	12.5429	9.60583	6.56278	8.43552	19.8379
	Mir335	22.2698	19.0681	40.839	21.511	38.3024	25.5085	23.6929	34.7241	33.6468	18.9544	64.5965	40.4704	37.9254
**Peg5**	*Nnat*	1509.41	1318.61	1324.86	1597.86	1541.48	1517.21	1568.31	1595.26	1840.92	2618.79	2871.72	2688.82	2391.68
	Blcap	71.0916	72.4541	72.82	80.297	84.208	79.1574	77.3478	77.3672	79.9559	40.6379	39.5498	45.3976	38.2852
**Igf2r**	*Igf2r*	110.492	115.272	113.994	109.186	119.2	122.948	128.489	108.081	125.36	85.9176	75.5797	86.4435	57.9538
	Slc22a3	1.66329	1.35295	1.47589	2.02008	1.38422	1.32172	1.36405	1.62092	1.12361	1.53017	1.16063	1.68196	1.4264
**Kcnq1ot1**	*Ascl2*	0.37608	0.8501	0.24027	0.3077	0.30046	0.23261	1.20032	0.5473	0.3147	0.61005	0.59277	0.31747	0.39525
	Th	7.75218	7.54598	6.00988	6.12024	8.01347	6.72978	7.81347	6.1498	7.32626	7.35387	6.498	6.88845	8.75496
	Xlr3b	0.89979	2.77355	6.38733	2.76077	9.13566	2.47355	6.38192	5.76786	2.58821	1.62176	1.9944	5.45935	8.47164
	Ampd3	6.71817	8.57157	10.0359	7.90286	8.30661	8.95516	9.73939	9.18344	6.79544	7.22972	6.01213	4.3884	7.17711

Reads of the imprinted genes in the female embryos

		M^+Z^+						M^-Z^+			M^-Z^-			
ICR	Gene	Emb 1	Emb 2	Emb 3	Emb 4	Emb 5	Emb 6	Emb 1	Emb 2	Emb 3	Emb 1	Emb 2	Emb 3	Emb 4
**Peg13**	*Kcnk9*	190	168	180	213	249	202	220	248	265	229	76	98	157
	Peg13	1798	1736	1626	1694	2019	1746	1792	1650	1998	1991	2805	2640	2266
	Trappc9	578	566	522	700	690	666	573	554	665	761	618	547	649
**Snrpn**	*Atp10a*	128	119	133	128	162	152	141	122	159	168	156	138	145
	Ube3a	4039	4025	3456	4386	5143	4803	4394	3807	4464	4653	4334	3693	4360
	Snord64	17	15	19	20	28	18	54	41	32	61	52	31	46
	Snrpn	1576	1697	1652	1622	1728	1701	3112	2187	2220	3522	3209	3144	3333
	Snurf	1547	1666	1620	1594	1680	1674	3024	2128	2159	3442	3129	3037	3253
	Ndn	4010	3889	3927	3750	3955	4076	6969	5078	5089	8511	7537	7546	8282
	Ipw	9	9	15	7	11	13	32	24	16	16	31	24	19
	Magel2	446	409	440	532	563	527	1038	931	791	1053	1013	1046	1208
	Mkrn3	187	167	154	192	160	183	307	250	240	449	429	364	384
	Peg12	504	420	377	514	473	496	982	670	725	1134	1270	1012	1123
**Impact**	*Impact*	979	958	842	1060	1191	1162	1167	1190	1290	1897	1739	1834	2189
**Cdh15**	*Cdh15*	460	492	430	428	466	568	458	504	475	506	437	491	611
**Peg3**	*Zim1*	1594	2002	1936	1915	2344	2233	2202	1848	1854	336	27	209	44
	Usp29	978	889	852	981	1162	1135	1034	922	1301	2085	2261	2040	2252
	Peg3	15694	14539	13539	15596	17964	16542	16812	13248	20195	30287	32869	28127	30161
	Peg3os	47	27	38	56	71	75	62	45	90	141	152	99	98
**Inpp5f**	*Inpp5f*	1652	1691	1496	1692	1882	1750	1659	1763	1837	3435	3001	2984	3275
**Zdbf2**	*Zdbf2*	2189	2141	1809	2257	2814	2558	2971	2788	3094	4421	5315	4513	4930
**Zrsr1**	*Zrsr1*	345	334	301	253	286	288	283	285	379	525	582	575	596
	Commd1	848	807	699	752	757	671	669	710	655	738	763	700	807
**Peg10**	*Calcr*	33	20	44	37	46	49	27	19	37	29	27	28	17
**Zac1**	*Plagl1*	9319	10622	10062	9915	12108	12161	11178	9938	11125	26332	21342	20547	24694
	Hymai	711	679	610	589	708	724	676	453	710	1357	1133	1124	1089
	Phactr2	1614	1641	1500	1816	2161	1938	1789	1709	1935	1101	1062	982	1066
**IG-DMR**	*Begain*	387	410	402	367	421	366	397	534	452	336	280	287	307
	Dio3	112	105	104	87	95	112	86	82	84	46	32	21	28
	Dio3os	29	32	19	22	19	25	15	27	27	12	12	7	13
	Dlk1	9996	12972	12956	9795	11178	12372	11232	11631	9772	2464	1970	2089	2676
	Rian	13972	14721	14635	14102	15766	14873	14687	16024	15472	30074	25580	25478	34798
	Rtl1	197	297	257	148	238	266	224	269	207	5	10	4	22
	AF357359	1312	1432	1478	1307	1591	1539	1533	1644	1487	2921	2634	2561	3582
	AF357426	37	16	17	29	29	30	23	25	26	43	51	25	73
	Meg3	17708	15597	15788	17162	19002	19029	20297	25210	21920	36198	33208	33695	53757
	Mirg	489	475	497	535	551	546	633	684	581	1278	1189	1155	1786
	AF357355	29	13	12	17	7	12	23	12	9	28	18	25	17
	AF357425	18	11	15	13	34	14	14	18	13	42	30	22	31
	Mir410	40	49	40	61	61	49	57	50	33	124	109	114	165
	Mir431	6	5	8	10	10	8	6	9	9	8	5	6	17
	Mir335	18	15	31	17	31	20	18	27	26	17	53	31	35
**Peg5**	*Nnat*	16632	14141	13710	17215	17008	16217	16243	16910	19393	32020	32121	28078	30090
	Blcap	1236	1226	1189	1365	1466	1335	1264	1294	1329	784	698	748	760
**Igf2r**	*Igf2r*	8137	8262	7884	7862	8790	8783	8894	7657	8826	7021	5650	6033	4873
	Slc22a3	48	38	40	57	40	37	37	45	31	49	34	46	47
**Kcnq1ot1**	*Ascl2*	5	11	3	4	4	3	15	7	4	9	8	4	6
	Th	114	108	83	88	118	96	108	87	103	120	97	96	147
	Xlr3b	12	36	80	36	122	32	80	74	33	24	27	69	129
	Ampd3	273	339	383	314	338	353	372	359	264	326	248	169	333

Table S6. Transcript per million (TPM) values are used to measure the expression levels of some imprinted genes in the male embryos.

TPMs of the imprinted genes in the male embryos

		$M^{+}Z^{+}$				$M^{-}Z^{+}$			$M^{-}Z^{-}$		
ICR	Gene	Emb 1	Emb 2	Emb 3	Emb 4	Emb 1	Emb 2	Emb 3	Emb 1	Emb 2	Emb 3
	Kcnk9	2.8236	3.3372	2.984	3.2471	3.3853	3.0774	3.1755	1.6128	0.2655	2.3264
Peg13	*Peg13*	47.743	46.81	45.096	47.786	50.072	44.73	48.087	58.779	53.059	49.299
	Trappc9	10.32	9.844	10.139	10.907	11.592	10.711	12.309	10.823	9.6738	9.9506
	Atp10a	3.0626	2.6997	2.913	4.0267	3.2984	3.6353	3.3342	2.6664	3.5247	4.0691
	Ube3a	53.786	52.703	48.511	50.707	46.014	47.75	44.537	45.941	38.367	38.807
	Snord64	73.597	56.311	55.946	63.647	142.04	87.064	87.483	86.728	81.704	107.81
	Snrpn	48.568	48.197	47.757	48.706	77.95	90.609	80.462	88.859	99.7	95.141
Snrpn	*Snurf*	97.03	96.303	95.794	96.794	153.15	178.93	159.31	175.37	195.12	188.35
	Ipw	1.0924	2.1174	1.1687	2.7748	2.7092	3.5076	4.2833	1.8742	3.4686	3.6382
	Ndn	289.72	275.66	293.27	282.27	455.62	492	444.3	519.03	592.75	567.96
	Magel2	11.848	12.64	11.54	17.775	27.877	27.92	24.574	29.007	25.563	25.695
	Mkrn3	9.0448	7.7375	8.6612	9.941	14.032	16.755	14.78	19.338	14.297	16.684
	Peg12	21.699	18.888	16.897	22.903	35.025	36.354	34.202	51.432	32.56	40.654
Impact	*Impact*	30.169	31.24	30.134	32.959	31.412	41.841	34.92	52.498	48.571	38.457
Cdh15	*Cdh15*	16.327	18.04	20.777	21.94	22.632	21.181	19.109	20.42	15.352	19.368
	Zim1	50.767	55.787	56.601	83.154	72.175	80.076	76.151	6.2085	1.7315	4.4676
Peg3	*Usp29*	7.7507	7.8521	7.0382	7.5184	8.8622	7.4856	7.3165	14.867	13.876	12.731
	Peg3	279.43	276.35	258.38	303.92	337.04	277.02	279.08	531.15	382.45	466.42
	Peg3os	4.6348	4.3421	3.1168	13.097	14.778	12.402	11.34	19.403	17.099	20.582
Inpp5f	*Inpp5f*	39.453	39.206	38.486	38.609	41.637	39.385	38.486	63.932	63.403	67.124
Zdbf2	*Zdbf2*	20.149	17.59	16.896	18.956	25.566	23.349	20.624	39.265	32.332	27.762
Zrsr1	*Zrsr1*	9.282	8.8804	10.067	7.0278	11.826	12.355	9.7705	13.751	11.929	13.871
	Commd1	95.381	100.71	100.74	86.176	90.937	88.808	93.527	83.952	75.979	85.473
Peg10	*Calcr*	0.7071	1.0611	0.753	1.0754	1.1273	0.769	1.0303	0.5987	0.8691	0.6511
	Plagl1	213.42	221	238.58	279.07	284.07	255.51	247.09	450.16	446.9	542.5
Zac1	*Hymai*	14.379	15.062	16.07	15.584	16.322	15.248	14.277	24.76	22.66	28.823
	Phactr2	22.721	24.551	21.767	24.96	24.333	21.988	21.571	13.739	10.231	10.632
	Begain	14.538	13.896	15.274	19.914	22.083	18.884	18.542	11.401	12.385	10.58
	Dio3	7.2017	4.992	7.3477	8.8588	6.6152	9.0353	7.2707	2.6512	2.012	2.5188
	Dio3os	2.7863	2.2278	2.3954	2.1896	2.5542	2.5721	2.3598	1.1832	0.474	1.7896
	Dlk1	279.7	276.34	355.72	340.82	366.56	335.39	312.31	45.351	47.679	71.631
	Rian	450.53	498.45	495.95	563.7	593.51	527.65	517.47	909.67	935.48	897.22
	Rtl1	4.0496	3.85	5.4933	4.5047	5.6073	4.4993	4.1734	0.2164	0.4386	0.32
	AF35735	4882.2	5888.6	6045.7	6156.8	6254.4	5664.1	5566.8	9749	9076.6	10856
**IG-DMR**	*AF35742*	15.957	36.728	21.949	66.497	16.96	29.888	30.031	61.598	162.86	43.653
	Meg3	123.14	156.03	124.28	249.86	264.22	220.4	214.58	418.07	416	337.26
	Mirg	44.1	52.644	43.963	58.796	64.403	60.781	54.117	110.99	100.8	103.28
	AF35735	16.312	14.593	27.617	17.929	30.008	20.146	30.111	21.313	122.95	19.702
	AF35742	45.191	16.893	7.1043	44.276	51.463	49.751	27.338	61.806	158.14	40.545
	Mir410	60.494	65.557	57.492	55.004	87.677	95.356	63.655	169.84	100.28	152.28
	Mir431	12.727	4.2697	6.7334	9.7918	10.926	11.003	8.2916	27.211	29.31	22.359
	Mir335	30.909	21.145	25.01	33.772	20.291	13.136	32.081	58.958	53.196	33.737
Peg5	*Nnat*	1504.1	1439.4	1258.1	1535.8	1656.3	1700	1822.3	2866.6	3336.7	2459.7
	Blcap	68.213	66.294	67.088	78.2	75.87	71.517	67.174	42.093	36.291	43.313
Igf2r	*Igf2r*	101.87	103.83	105.06	122.92	122.44	110.11	109.53	78.052	87.523	80.736
	Slc22a3	1.324	1.7397	1.7162	1.3824	1.2584	1.5534	1.7252	0.9829	0.8662	1.163
Kcnq1ot1	*Ascl2*	0.3869	0.3214	0.2281	0.3948	0.2643	0.5324	1.0142	1.3654	2.7075	0.8677
	Th	4.2472	6.6101	5.4985	8.0674	8.0458	6.3374	7.7584	8.8729	9.5875	6.8468
	Xlr3b	4.1322	3.8446	6.4419	7.4786	7.3785	9.2879	6.3773	7.3166	11.022	11.324
	Ampd3	7.7754	7.518	9.8745	8.3709	9.5998	8.3318	7.3254	5.8356	2.4115	7.5881

Reads of the imprinted genes in the male embryos

		$M^{+}Z^{+}$				$M^{-}Z^{+}$			$M^{-}Z^{-}$		
ICR	Gene	Emb 1	Emb 2	Emb 3	Emb 4	Emb 1	Emb 2	Emb 3	Emb 1	Emb 2	Emb 3
	Kcnk9	246	200	189	198	185	167	196	91	17	142
Peg13	*Peg13*	2531	1707	1738	1773	1665	1477	1806	2018	2067	1831
	Trappc9	826	542	590	611	582	534	698	561	569	558
	Atp10a	188	114	130	173	127	139	145	106	159	175
	Ube3a	5559	3747	3645	3668	2983	3074	3261	3075	2914	2810
	Snord64	38	20	21	23	46	28	32	29	31	39
	Snrpn	2234	1525	1597	1568	2249	2596	2622	2647	3370	3066
Snrpn	*Snurf*	2200	1502	1579	1536	2178	2527	2559	2575	3251	2992
	Ipw	9	12	7	16	14	18	25	10	21	21
	Ndn	5369	3514	3951	3661	5296	5679	5833	6229	8072	7374
	Magel2	620	455	439	651	915	910	911	983	983	942
	Mkrn3	260	153	181	200	253	300	301	360	302	336
	Peg12	643	385	364	475	651	671	718	987	709	844
Impact	*Impact*	1300	926	944	994	849	1123	1066	1465	1538	1161
Cdh15	*Cdh15*	521	396	482	490	453	421	432	422	360	433
	Zim1	1995	1508	1617	2287	1779	1960	2120	158	50	123
Peg3	*Usp29*	1280	892	845	869	918	770	856	1590	1684	1473
	Peg3	20776	14134	13966	15815	15718	12829	14700	25575	20896	24296
	Peg3os	45	29	22	89	90	75	78	122	122	140
Inpp5f	*Inpp5f*	2266	1549	1607	1552	1500	1409	1566	2378	2676	2701
Zdbf2	*Zdbf2*	2969	1783	1810	1955	2363	2143	2153	3747	3501	2866
Zrsr1	*Zrsr1*	468	308	369	248	374	388	349	449	442	490
	Commd1	1107	804	850	700	662	642	769	631	648	695
Peg10	*Calcr*	31	32	24	33	31	21	32	17	28	20
	Plagl1	12792	9112	10396	11707	10680	9539	10492	17474	19684	22781
Zac1	*Hymai*	816	588	663	619	581	539	574	910	945	1146
	Phactr2	2306	1714	1606	1773	1549	1390	1551	903	763	756
	Begain	508	334	388	487	484	411	459	258	318	259
	Dio3	151	72	112	130	87	118	108	36	31	37
	Dio3os	40	22	25	22	23	23	24	11	5	18
	Dlk1	15104	10265	13965	12881	12416	11281	11948	1586	1892	2710
	Rian	18099	13774	14484	15849	14955	13203	14727	23666	27616	25252
	Rtl1	289	189	285	225	251	200	211	10	23	16
	AF35735	1644	1364	1480	1451	1321	1188	1328	2126	2246	2561
**IG-DMR**	*AF35742*	12	19	12	35	8	14	16	30	90	23
	Meg3	17907	15608	13139	25431	24101	19964	22107	39373	44456	34361
	Mirg	643	528	466	600	589	552	559	1048	1080	1055
	AF35735	13	8	16	10	15	10	17	11	72	11
	AF35742	35	9	4	24	25	24	15	31	90	22
	Mir410	55	41	38	35	50	54	41	100	67	97
	Mir431	13	3	5	7	7	7	6	18	22	16
	Mir335	34	16	20	26	14	9	25	42	43	26
Peg5	*Nnat*	22555	14848	13716	16119	15579	15879	19360	27839	36769	25842
	Blcap	1614	1079	1154	1295	1126	1054	1126	645	631	718
Igf2r	*Igf2r*	10210	7158	7655	8622	7697	6874	7777	5066	6446	5669
	Slc22a3	52	47	49	38	31	38	48	25	25	32
Kcnq1ot1	*Ascl2*	7	4	3	5	3	6	13	16	36	11
	Th	85	91	80	113	101	79	110	115	141	96
	Xlr3b	75	48	85	95	84	105	82	86	147	144
	Ampd3	430	286	397	324	333	287	287	209	98	294
